# Functional traits reveal unaccounted diversity within *Sonchus asper* (L.) Hill (Asteraceae) in the northern Mediterranean and enable the reinstatement of ancient taxa

**DOI:** 10.1093/aobpla/plag040

**Published:** 2026-09-02

**Authors:** José A Mejías, Myong-Suk Cho, Regina Berjano, Anass Terrab, Pedro Costales-Maestre, José L Silva, Seung-Chul Kim

**Affiliations:** Departamento de Biología Vegetal y Ecología, Universidad de Sevilla, Avda. Reina Mercedes s/n, Sevilla 41012, Spain; Department of Biological Sciences, Sungkyunkwan University, 16419 Suwon, South Korea; Departamento de Biología Vegetal y Ecología, Universidad de Sevilla, Avda. Reina Mercedes s/n, Sevilla 41012, Spain; Herbario de la Universidad de Sevilla (SEV). Servicio General de Herbario. Centro de Investigación, Tecnología e Innovación (CITIUS II), Avda. Reina Mercedes s/n, Sevilla 41012, Spain; Departamento de Biología Vegetal y Ecología, Universidad de Sevilla, Avda. Reina Mercedes s/n, Sevilla 41012, Spain; Departamento de Biología Vegetal y Ecología, Universidad de Sevilla, Avda. Reina Mercedes s/n, Sevilla 41012, Spain; Departamento de Biología Vegetal y Ecología, Universidad de Sevilla, Avda. Reina Mercedes s/n, Sevilla 41012, Spain; Department of Biological Sciences, Sungkyunkwan University, 16419 Suwon, South Korea

**Keywords:** AFLPs, cpDNA haplotype, cryptic herbarium species, ITS/ETS, mating system divergence, Mediterranean phylogeography, plant life cycle, rhizomatous plants, selfing syndrome

## Abstract

Functional traits express the ecological and evolutionary attributes of individuals that compose taxa, providing robust criteria to detect speciation when integrated with phylogenies. We tested this approach in *Sonchus asper* (L.) Hill, a putatively self-fertilizing annual weed that has recently been reported to exhibit extensive floral and life-form variation across the northern Mediterranean. This variability was already documented in the nineteenth century, but it is incongruent with current subspecific classifications based on leaf arrangement. We sampled 40 populations encompassing this variation, assigning each to (a) one of the four morphotypes defined by floral traits and life forms and (b) a leaf arrangement group. To assess their taxonomic relevance, we tested grouping characters as functional traits and analysed the consistency of these assignments with the phylogeny (ITS/ETS and chloroplast DNA markers) and genetic structure [amplified fragment length polymorphism (AFLP) markers]. Phylogenetic analysis using floral traits and life forms identified three well-supported clades that contradict leaf-arrangement taxonomy. The earliest-diverging clade comprises the common, annual, self-fertilizing *S. asper*. The remaining two clades consist of self-incompatible plants: one contains the rhizomatous morphotype, whereas the other groups annual and biennial plants. These life-history strategies exhibited distinct AFLP-based genetic profiles, alongside complex cpDNA phylogeographical patterns. The congruence between DNA analyses and functional value of floral and life-form traits corroborates the proposed morphotypes as distinct evolutionary lineages. This reveals unaccounted-for biodiversity and supports reinstating historical taxa. Our study shows that combining functional traits with genealogical relationships provides a powerful operational taxonomic criterion that integrates ecological and evolutionary species concepts.

## Teaser

Flower size and life form are identified as functional traits of evolutionary significance in *Sonchus asper* (Asteraceae), in contrast to the leaf-arrangement criterion commonly used for subspecific division. DNA analyses support our hypothesis and reveal unaccounted-for biodiversity in the northern Mediterranean Basin. This confirms that the current *S. asper* circumscription constitutes a species complex and justifies the reinstatement of three taxa described during the 19th century. Furthermore, we demonstrate that integrating phylogenetics with functional traits provides a robust framework for resolving evolutionary histories and preventing the persistence of ‘cryptic herbarium species’.

## Introduction

Functional traits are well-defined, measurable morphological, physiological, or phenological characteristics that strongly influence an organism's performance and ecological role within a community (McGill et al. 2006, Violle et al. 2007). Therefore, grouping individuals according to true functional traits allows the identification of their primary biological attributes and the delineation of their respective species’ niches (Violle and Jiang 2009, Albert et al. 2011, Mudrák et al. 2019). This approach aligns with the idea that every species occupies its own particular ‘adaptive zone’, the central core of the Ecological Species Concept (van Valen 1976, Andersson 1990). Moreover, if functional traits describe the adaptive mechanisms of a species, they also reflect its ‘unitary evolutionary role and tendencies’ as a lineage, linking it to the Evolutionary Species Concept (Simpson 1951), commonly recognized as the primary or ontological idea of species (Wiley 1978, Wiley and Mayden 2000, Zachos 2016). All these considerations suggest that species delimitation based on independently evolving lineages can be strengthened by incorporating functionally meaningful traits into taxonomic practice.

Traits with functional value are widely present in plant systematics. However, relatively few studies have explicitly linked taxonomically informative characters to the functional-trait framework (see Supplementary Table S1), even though the term was coined four decades ago (Calow 1987, Violle et al. 2007). The connection between these two perspectives is particularly evident, for example, in the long-recognized importance of reproductive characters in plant taxonomy (Lloyd 1965, Ornduff 1969, Anderson et al. 2002). Furthermore, understanding how these traits function globally has led to the definition of reproductive syndromes in plants, among which the selfing syndrome is one of the most widespread (Barrett 2003, Sicard and Lenhard 2011, Cutter 2019).

This latter syndrome reflects a recurrent evolutionary transition from an outbreeding to an inbreeding mating system. Selfing plants do not strictly require pollen vectors, so, compared to their outbreeding relatives, they have significantly smaller, less specialized, and less attractive flowers, with a lower incidence of herkogamy or dichogamy and reduced insect rewards and pollen content (Ornduff 1969, Stebbins 1974, Anderson et al. 2002, Goodwillie et al. 2005, Sicard and Lenhard 2011, Orsucci and Sicard 2021), as well as the loss of self-incompatibility (Lloyd 1965, Sicard and Lenhard 2011, Freyman and Höhna 2019, Orsucci and Sicard 2021). These evolutionary transitions are also associated with life-history traits. Self-incompatibility is more common in perennial than in annual species, whereas annuals tend to exhibit more complete selfing syndromes (Stebbins 1974, Vallejo-Marín and O'Brien 2007, Barrett et al. 2014, Friedman 2020, Ferrer et al. 2025).

Recently documented variation in flower size and life history within *Sonchus asper* (Asteraceae, Cichorieae, Hyoseridinae) in the Mediterranean (Mejías 2002, 2017, Mejías et al. 2012) provides a study system in which to evaluate the association between functional traits and systematics. This species is commonly considered an annual (exceptionally biennial) cosmopolitan weed with a high capacity to produce achenes by self-fertilization (Lewin 1948, Hsieh et al. 1972, Mejías 1992) and relatively inconspicuous flower heads (Mejías 1992). At present, the variability within *S. asper* is addressed by subdividing it into two subspecies: the nominate subspecies (*S. asper* subsp. *asper*) and another named either *S. asper* subsp. *glaucescens* (Jord.) Ball or *S. asper* subsp. *nymanii* (Tineo & Guss.) Hegi (Fig. 1c–e), present mainly in the Mediterranean (Boulos 1973). The delimitation of the latter relies primarily on leaf arrangement, which has been interpreted as indicative of a biennial life cycle. However, the original descriptions of the two intended basionyms for this subspecies—*S. glaucescens* Jord. and *S. nymanii* Tineo & Guss.—do not emphasize leaf arrangement but rather the size and colour of the flower heads, which were reported to be approximately twice as large as those of *S. asper* and more intensely coloured (Gussone 1844: 860; Jordan 1847: 76). These taxa were also reported to be biennial and rhizomatous perennial plants, respectively, and were described using material from southern France and Sicily. Furthermore, among the remaining *S. asper* synonyms (Boulos 1973, IPNI 2026; https://www.ipni.org, last accessed on 15 July 2026), the presence of large heads is also assumed in the protologue of *S. graecus* Reut. ex E. Weiss (Weiss 1869), a name assigned to plants from the Aegean. This is in line with the recent report of plants with large ligule florets (and therefore large flower heads in anthesis) and long anthers, supposedly associated with xenogamy, within the current circumscription of the species in the Mediterranean (Mejías 2002, Mejías et al. 2012). Collectively, these observations suggest that *S. asper* actually constitutes a complex where changes in the sexual system and life form are important drivers of diversification.

**Figure 1:**
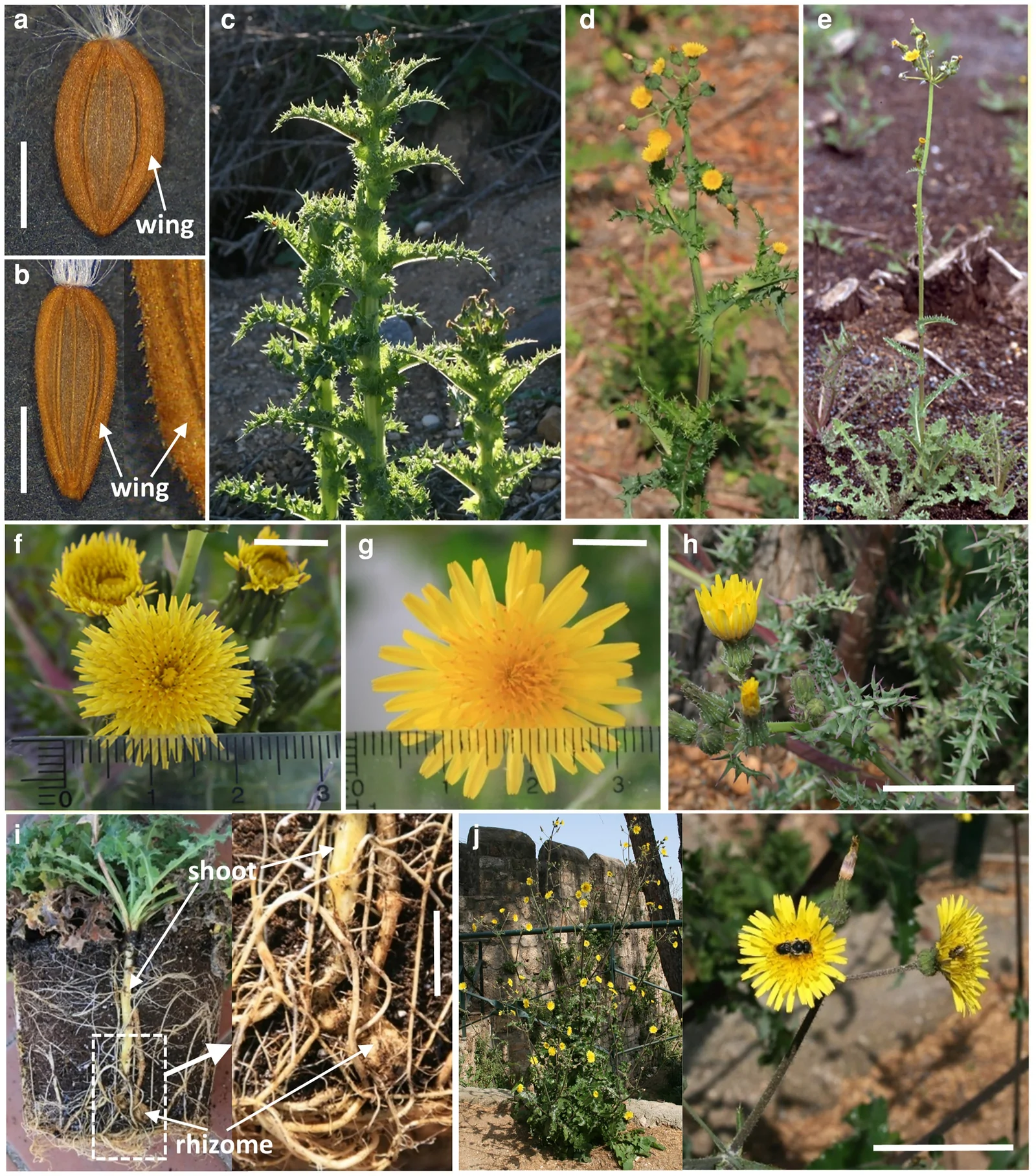
Variability in reproductive and vegetative characters in *Sonchus asper* from the northern Mediterranean. (a) Achene of a common *S. asper* (L.) Hill (A.MAR[g]). (b) Achene of the rhizomatous perennial morphotype *S. nymanii* Tineo & Guss. (N.ALI[AG]). (c) Leaf arrangement of common *S. asper* (L.) Hill plants with well-developed leaves exclusively cauline (associated with subsp. *asper*: A.MJO[A]). (d) Detail of a common *S. asper* (L.) Hill plant of intermediate leaf arrangement pattern and doubtful subspecies assignment ([AG]: Portugal, São Pedro Moel). (e) leaf arrangement of a common *S. asper* (L.) Hill plant with a well-developed basal rosette and small cauline leaves (associated with subsp. *glaucescens*: Spain, Seville [G]). (f) Flower heads of common *S. asper* (L.) Hill during anthesis (A.CAB[A]). (g) Flower head of the morphotype *S. glaucescens* Jord. during anthesis. (G.GAU[g]). (h) Detail of the somewhat glaucous and prickly morphotype, with rigid leaves, *S. glaucescens* Jord. (G.GAU[g]). (i) Vegetative multiplication of the rhizomatous perennial morphotype *S. nymanii* Tineo & Guss. (N.SCI[AG]). (j) Habit and blooming details of the morphotype *S. graecus* Reut. ex Weiss (Turkey, Antalya: SEV256670). Scale bars: 1 mm (a, b), 1 cm (f, g) or 5 cm (h–j).

To assess the biodiversity implications of the reported variability in *Sonchus asper*, we addressed four specific questions: (1) Do floral size and anther length reflect mating system divergence and qualify as functional traits? If so, we expected bimodal distributions of floral morphological characters and low selfing ability (indicating a xenogamous sexual system) in the large-floret plants, contrasting with high selfing capacity in typical plants with small florets and short anthers (Lewin 1948, Hsieh et al. 1972, Mejías 1992). A lack of clear divergence would reflect gradual adaptations rather than divergent strategies. (2) How is variation in life history linked to the mating system and leaf arrangement? We expected these traits to be associated, thereby enabling the characterization of distinct lineages. (3) Are the phylogenetic relationships and genetic structure of northern Mediterranean populations consistent with the variability in mating system, life form, and leaf arrangement? We combined nrDNA (ITS/ETS) and cpDNA sequencing with amplified fragment length polymorphism (AFLP) analyses to assess evolutionary relationships. We expected substantial genetic differentiation to be consistent with functional traits, reflecting the evolutionary processes that shaped potential lineages. (4) Finally, in light of our findings, what taxonomic treatment best reflects the biodiversity of the *S. asper* complex in the northern Mediterranean Basin?

## Materials and methods

### Study plant: variability and morphotype assignment

*Sonchus asper* s. l. is part of a phylogenetic clade of around 10 taxa within *Sonchus* (Kim et al. 2007), 7 of which are present in the Mediterranean Basin and 4 of which are endemic to the region (Boulos 1973). The clade also includes *S. oleraceus*, which, together with *S. asper*, represents one of the most important and globally widespread weeds among the nearly 100 species of *Sonchus*. Both species probably originated in the Mediterranean, as did the genus itself (Mejías and Andrés 2004) and the tribe Cichorieae (Funk et al. 2005). *S. asper* is a herbaceous plant with erect stems, 15–210 cm tall, whose young specimens and shoots form rosettes of spathulate leaves, from which emerges a floriferous stem, solitary or with erect-patent basal branches, bearing amplexicaul leaves with rounded auricles. The leaves are entire or pinnatifid to pinnatipartite, rarely pinnatisect, with ovate, triangular, or even linear lobes, with a denticulate margin bearing variably rigid spines. Flower heads are grouped in one to several panicles and contain 80–360 yellow florets, of which the outermost are the largest and determine the size of the flower head during anthesis. Achenes are oblanceolate, elliptic or oblong, strongly compressed, with marginal wings and three longitudinal ribs per side, which in most cases are single, although the lateral ones are frequently double (Fig. 1a and b). The pappus is more or less deciduous. This achene morphology is unique to the *S. asper* complex in the northern Mediterranean. In the southern Basin, it is also present in the endemic *S. mauritanicus* and *S. macrocarpus*, which are close relatives of *S. asper* (Boulos 1973, Kim et al. 2007). Similar achenes also appear in *S. hydrophilus* and *S. kirkii*, the two components of the clade restricted to the South-West Pacific.

The floriferous stem may bear well-developed leaves or reduced leaves with a persistent basal rosette (sub-rosulate habit). The current taxonomic treatment of *S. asper* proposes that plants exhibiting the former condition should be interpreted as annual (*S. asper* subsp. *asper*), whereas those showing the latter are considered biennial (subsp. *glaucescens* = subsp. *nymanii*; Boulos 1973, 1976). However, the identification of these subspecies is ambiguous due to continuous variation in leaf arrangement and the absence of roots in voucher specimens. Furthermore, this classification overlooks both the observed variability in flower size and 19th-century reports of perennial plants.

To account for this variability, we independently labelled the studied material based on two criteria: (a) morphotypes inferred from the protologues (plant morphotype) of *S. asper* (Hill 1756), *S. nymanii* (in Gussone 1844), *S. glaucescens* (Jordan 1847), and *S. graecus* (Weiss 1869) and (b) the current division into two subspecies based on leaf arrangement. For the first criterion, we relied on protologue descriptions in terms of floret size (specifically, the length and width of outer ligules, and anther length in both outer and central florets), as well as observations of life forms and leaf traits (which are robust, distinctly glaucous, and have rigid and sharp spines in the *S. glaucescens* morphotype). Measurements were taken from florets of dried voucher specimens, which were often briefly boiled in water with a few drops of detergent to allow careful spreading. We then tested for multimodality in the distributions of floret traits using the functions ‘locmodes’ and ‘modetest’ of the R package *multimode* v.1.5 (Ameijeiras-Alonso et al. 2021).

Morphotype assignments were indicated by prefixing each population acronym with a letter (A = *S. asper*, N = *S. nymanii*, G = *S. glaucescens*, R = *S. graecus*). To denote leaf arrangement, a bracketed letter was appended to each acronym: [A] for plants with dense, well-developed, exclusively cauline leaves, or [a] for those with mostly cauline leaves (both associated with subsp. *asper*); [G] for plants with a well-developed basal rosette and small cauline leaves, or [g] for those also displaying some well-developed cauline leaves (both associated with subsp. *glaucescens*); [AG] for intermediate individuals that could not be reliably assigned (see Supplementary Table S2, Supplementary Figure S1).

### Plant distribution and material collection

We restricted our sampling primarily to the northern Mediterranean region, as the three taxa we tested as potential evolutionary lineages were originally described from material collected in the area. Furthermore, the complete range of variation in leaf arrangement is present in this region (Boulos 1973). To analyse the geographical distribution of variation, we measured and revised 227 voucher specimens from the herbaria B, BC, BM, C, COI, FI, GDAC, JACA, L, MA, MPU, SEV, and TLJ. Additionally, we examined 103 specimens from 78 populations collected during our field trips in Portugal, Spain, southern France, Italy, Greece, Turkey, and northern Morocco between May 2009 and May 2017 (see Supplementary Appendix S1). Geographic patterns of floret traits and leaf arrangement were assessed using Mantel tests, performed via the function ‘mantel’ in the R package *vegan* v.2.7-2 (Oksanen et al. 2013). Differences in floret traits among leaf-arrangement groups were evaluated using a one-way ANOVA or a non-parametric Kruskal–Wallis test, based on data distribution properties.

During these field trips, we also sampled fresh leaf tissue for DNA analysis—which was dried and stored in silica gel until DNA extraction—alongside mature seeds from 10 to 20 individuals across 40 wild populations (see Supplementary Table S2, Supplementary Figure S1). For the ITS/ETS and cpDNA analyses, we used a total of 143 samples from 39 populations (using three to five individuals per population, with the exception of population A.VSE[A], for which only two samples were available). For AFLP fingerprinting, we used 240 samples from 24 populations (7 to 12 individuals per population). We visited original locations reported in the protologues of *S. nymanii* and *S. glaucescens* to ensure the successful sampling of these morphotypes, given their restricted distributions.

### Reproductive system

Plants grown from achenes collected in 11 wild populations, alongside young plants transplanted from three additional populations, were cultivated in experimental garden facilities at the University of Seville (see Supplementary Table S2). Because typical *S. asper* is considered self-compatible with a high selfing capacity (Lewin 1948, Hsieh et al. 1972, Mejías 1992), observations for this morphotype were restricted to three populations from northern Italy and southern Spain. Of these, two populations, A.PEL[a] and A.CAB[A], possessed well-developed cauline leaves, whereas the third, A.MAR[g], was sub-rosulate. Plants were grown in plastic pots with a 3:1 (v/v) peat–perlite substrate, supplemented with a slow-release solid organic fertilizer (Osmocote 12-months; 3.5 g/L substrate).

For each adult individual (*n* = 126; mean = 9 per population), three pollination treatments were conducted in a pollinator-free greenhouse under standard conditions (12 h of daily light, 18–22°C): autonomous self-pollination (ASP), forced self-pollination (FSP) and multiple individual cross-pollination (MCP)―the latter using pollen mixed from multiple individuals within the same population. Manual pollinations (FSP and MCP) were performed during anthesis using cotton swabs or, when the flower heads were close enough, by gently rubbing them against one another (Silva et al. 2016), repeating the process over 2–4 days for each flower head. Anthesis typically lasted 2 days in small-sized flower heads (*S. asper* morphotype) and 3–4 days in the others. Florets opened daily within each flower head, completing pollen presentation and stigma exposure within hours; however, in some cases flower heads prolonged anthesis by one or two additional days without opening new florets, probably to increase fertilization opportunities (Mejías 1992).

Following flowering, individual flower heads were covered with clean tea bags and harvested once mature. For each plant, the index of self-incompatibility (ISI; Lloyd 1965, Raduski et al. 2012) was calculated: ISI = 1 − (relative selfing success/relative out-crossed success). For the R.KAS[g] population, we estimated selfing success from the ASP treatment. The population-level ISI was calculated as the arithmetic mean of the individual indices. Mating systems were categorized following Bawa (1974) and Raduski et al. (2012) as self-incompatible (SI; ISI ≥ 0.8), partially self-incompatible (pSI; 0.2 < ISI < 0.8), and self-compatible (SC; ISI ≤ 0.2). Non-normally distributed data―even after attempted transformation―were analysed using SPSS software (v29.0). Differences in fruit set among pollination treatments were evaluated using the non-parametric Friedman test for repeated measures, followed by *post hoc* pairwise comparisons via Wilcoxon signed-rank tests with a Bonferroni correction (ASP vs. FSP and FSP vs. MCP).

### ITS/ETS sequencing and sequence data analyses

PCR amplification of ITS was performed as described previously (Lee et al. 2005, Silva et al. 2015). The ETS region was amplified using the primers designed by Linder et al. (2000). PCR products were purified with GENEALL EXPIN PCR SV (GeneAll Biotechnology, Seoul, Korea), and subsequent DNA sequencing was done at the Geno Tech Corp. (Daejeon, Korea) using the same PCR primers. Base calling, sequence editing, and alignment were performed with Geneious R v.11.1.5 (Biomatters, Auckland, New Zealand). To evaluate the consistency between morphotype assignment or leaf arrangement and phylogenetic relationships within the complex, we conducted maximum likelihood (ML) analyses using W-IQ-TREE (Nguyen et al. 2015, Trifinopoulos et al. 2016). ML trees were generated with ultrafast bootstrap support (BS) calculated from 1000 bootstrap replicates to determine clade robustness (Hoang et al. 2018). Best-fit substitution models were estimated according to the Bayesian information criterion using ModelFinder (Kalyaanamoorthy et al. 2017) implemented in IQ-TREE. The resulting ML phylogenetic tree was visualized using the Interactive Tree of Life (iTOL) online tool v.6 (Letunic and Bork 2021). Sequences from the close relatives *S. kirkii* and *S. hydrophilus* were used as outgroups.

### cpDNA sequencing and sequence data analysis

We surveyed 17 noncoding regions of cpDNA based on Shaw et al. (2007) and selected the three regions with the largest number of potentially informative characters (PICs) in the *S. asper* complex: *ndhJ*-*trnF(GAA)*, *atpI*-*atpH*, and *psbJ*-*petA*. These three regions were amplified using the primers described in Shaw et al. (2007). The protocols for PCR purification, DNA sequencing, sequence data process (i.e. base calling, sequence editing, and alignment), and phylogenetic analysis followed the same procedures described above for ITS/ETS. The best-fit model for the three combined cpDNA regions was ‘F81 + F + I’. Additionally, a haplotype network based on the concatenated cpDNA was also constructed using statistical parsimony, implemented in the program TCS v.1.21 (Clement et al. 2001). Gaps were treated as missing data, and the connection limit was set to 95% in accordance with Hart and Sunday (2007).

### AFLP fingerprinting and analyses of AFLP data

The AFLP procedure followed established protocols (Vos et al. 1995). The six primer combinations selected for the selective PCR were *Eco*RI (Fam)-ACC/*Mse*I-CTA, *Eco*RI (Fam)-ACT/*Mse*I-CTG, *Eco*RI (Vic)-AAG/*Mse*I CAC, *Eco*RI (Vic)-ATG/*Mse*I CTGA, *Eco*RI (Ned)-AAC/*Mse*I-CTC, and *Eco*RI (Ned)-AGC/*Mse*I-CATA. Given the low level of differentiation detected within the small-floret clade (common *S. asper*) in the ITS/ETS tree, we chose five populations of this morphotype, trying to encompass leaf arrangement variability, and sampled the remaining populations from large-floret plants to focus the analysis mainly on potential differentiation within this poorly understood group. In addition to the 240 samples analysed, 25 additional individuals were replicated to exclude non-reproducible bands and to calculate the error rate, following Bonin et al. (2004). The fluorescence-labelled selective amplification products were separated by capillary gel electrophoresis at the ‘Genomic Unit’ (Universidad Complutense, Madrid, Spain) on an automated sequencer (3730 DNA Analyser, PE Applied Biosystems, Foster City, CA, USA). Raw data were exported to GeneMarker 1.8 (SoftGenetics, PA, USA) for automatic scoring of fragments after normalization of the profiles.

We used principal coordinate analysis (PCoA) to investigate genetic distances and relationships among populations. The matrix of Jaccard similarities among individuals was calculated with the ‘vegdist’ function in the R package *vegan* v.2.7-2 (Oksanen et al. 2013). The PCoA was performed using the resulting matrix and the ‘pco’ function in the R package *ecodist* v.2.1.3 (Goslee and Urban 2007).

The overall population genetic structure was explored using Bayesian clustering analysis implemented in STRUCTURE 2.3.4 (Pritchard 2010). Analyses were based on an admixture ancestry model with correlated allele frequencies for a range of *K* genetic clusters from 1 to 11, with 10 replicates for each *K* value. The analyses were performed with a burn-in period of 100 000 and a Monte Carlo Markov Chain (MCMC) run length of 10^6^ iterations. The most likely *K* value was determined based on the *ΔK* and Mean Ln*P*(*K*) (Evanno et al. 2005, Pritchard 2010) using Structure Harvester (Earl and vonHoldt 2012). For *K* = 2 and *K* = 3, the Full search method with 1000 replicates was used. For *K* = 5, we used the Greedy algorithm, with 30 000 random input orders.

In addition, we analysed the overall population genetic structure using the BAPS software. The maximum number of genetic clusters (*K*) was set from 2 to 24. We performed six replicates for each *K* value, and the results were averaged based on the resulting likelihood scores. Subsequently, an admixture analysis (Corander and Marttinen 2006) was conducted with the following settings: a minimum cluster size of four individuals; 100 iterations to estimate the admixture coefficients for the individuals; 200 simulated reference individuals from each population; and 20 iterations to estimate the admixture coefficients for the reference individuals.

We assessed patterns and levels of population genetic differentiation by performing analyses of molecular variance (AMOVA) using four different groupings conducted with ARLEQUIN v.3.01 (Excoffier et al. 2005).

## Results

### Variability in functional reproductive traits and mating systems

The characters selected to assess flower size (length and width of the outer ligules, and length of the outer and central anthers) were highly correlated (*P* < 0.001, *ρ* = 0.726–0.937; *N* = 284–327) with bimodal frequency distributions (*P* = 0.034 for ligule width and *P* < 0.001 for the three remaining characters. Fig. 2; see Supplementary Table S3). This distinguished two plant groups: one with small florets, whose outer ligules and anthers seldom exceeded 6.0 and 2.0 mm, respectively, and another with large florets whose outer ligules and anthers were consistently longer than 6.6 and 2.0 mm. The first group includes those plants that were assigned to the common morphotype of *S. asper*, and the second includes the morphotypes *S. nymanii*, *S. glaucescens*, and *S. graecus*. The variability of these four floret traits exhibited a significant geographic pattern (Mantel test: *P* < 0.001, *r* = 0.2546–0.3293. See Supplementary Figure S1, Supplementary Appendix S1), with large-floret morphotypes being absent from western areas (Fig. 3).

**Figure 2:**
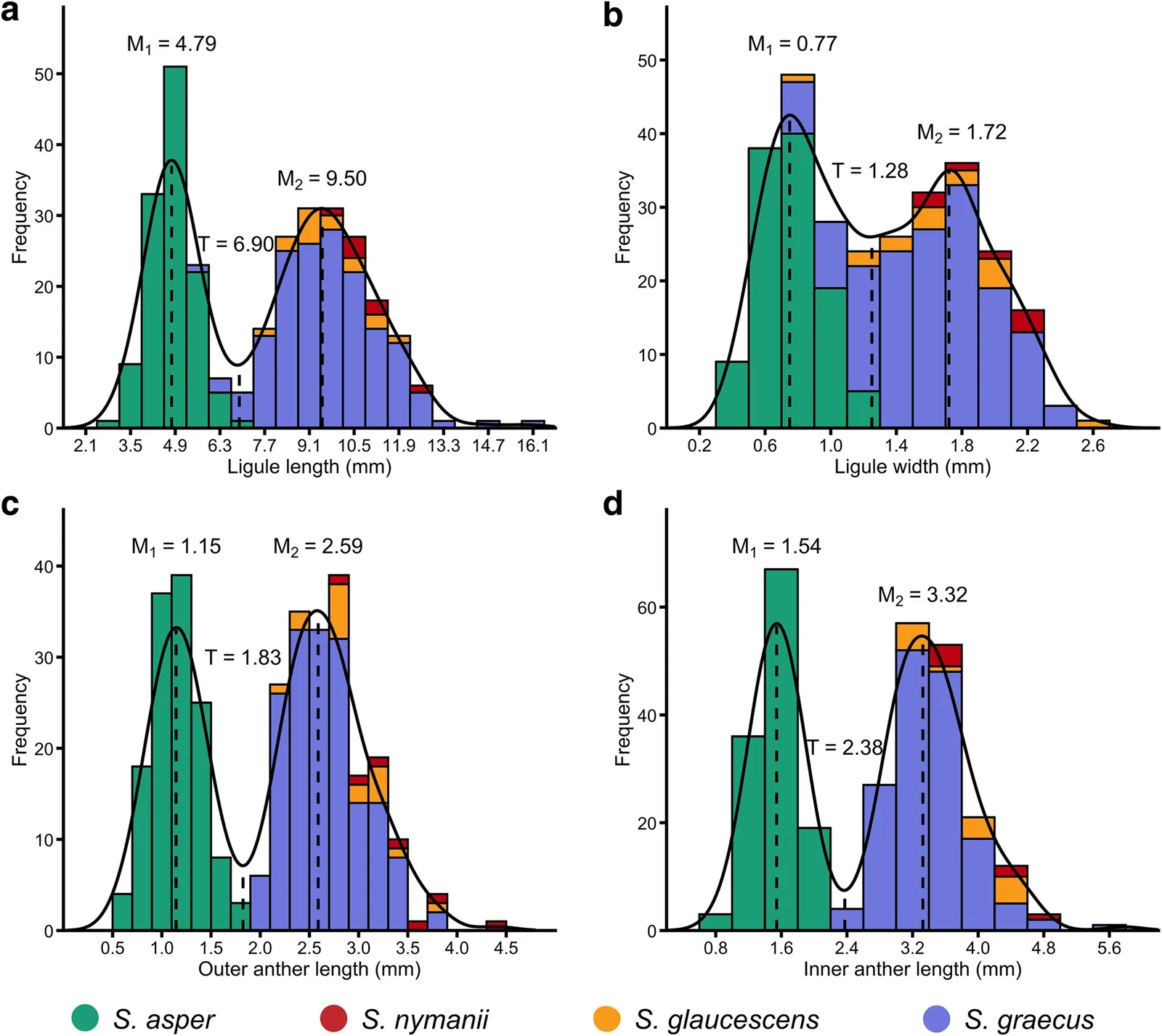
Frequency histograms of the functional traits related to the mating system in the *Sonchus asper* taxonomic complex in the northern Mediterranean, with indication of the morphotype assigned to the samples. We analysed the length (a, *N* = 299) and width (b, *N* = 285) of a ligule in an outer floret, and the anther tube length in an outer (c, *N* = 327) and an inner (central) floret (d, *N* = 303) of a flower head for each specimen studied (see Supplementary Appendix S1). M_1_ and M_2_ = modes of the bimodal distribution, and T = antimode, estimated according to Zhang et al. (2003).

**Figure 3:**
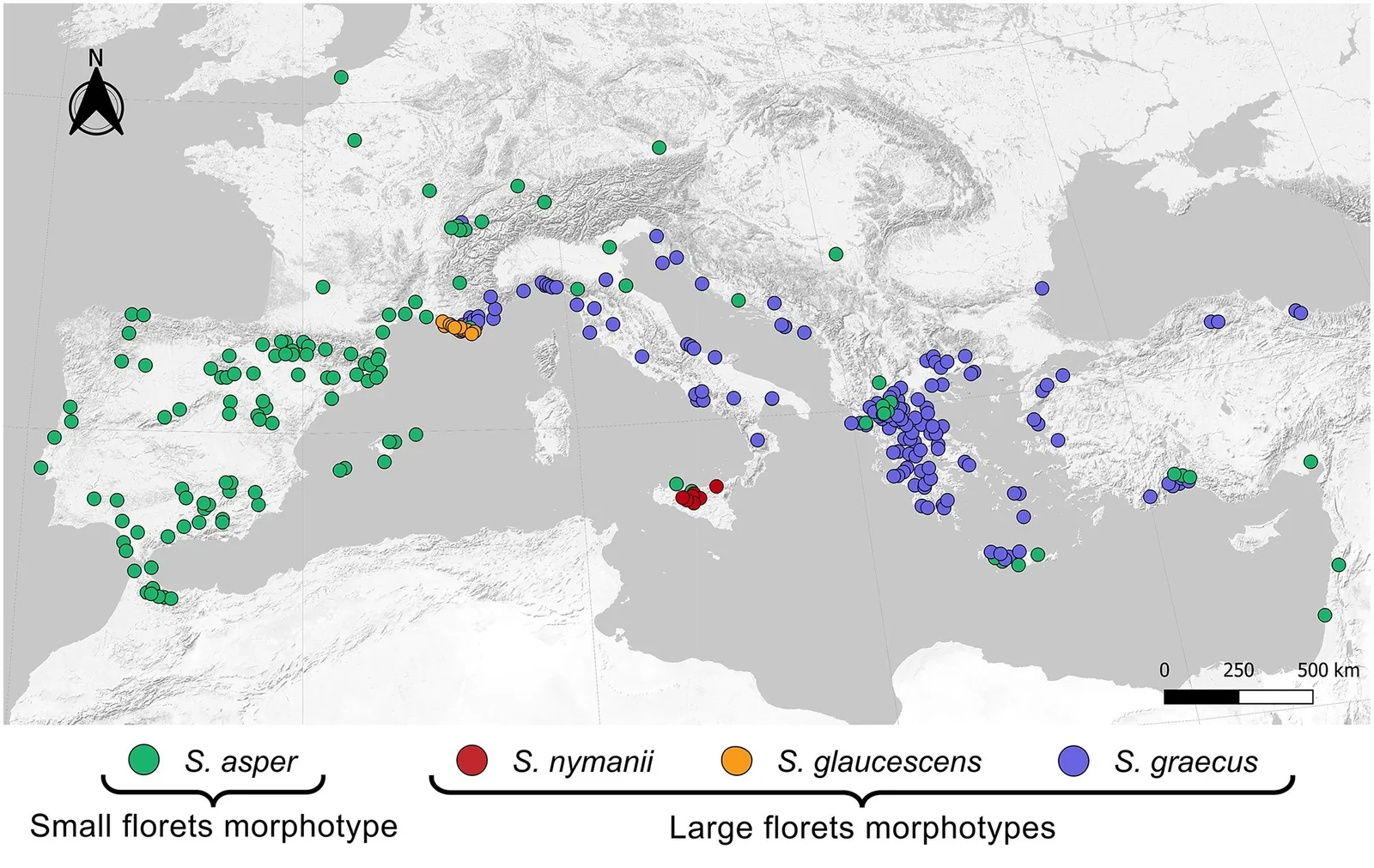
Geographical distribution of the localities of origin of the dried specimens (*N* = 322) used for the analysis of floret size variability within the *Sonchus asper* complex, with indication of the assigned morphotype.

Leaf arrangement also showed a significant, although weak, geographical correlation (Mantel test: *P* < 0.001, *ρ* = 0.0543). Overall, the sub-rosulate habit ([G] + [g]) was more common in eastern areas and among large-floret morphotypes, whereas small-floret plants more frequently displayed well-developed cauline leaves, with the typical subsp. *asper* habit ([A]) being exclusive to the latter. No differences in floret traits were detected among leaf-arrangement groups (see Supplementary Table S4) in either small-floret plants (*P* = 0.051–0.837) or large-floret plants (*P* = 0.305–0.935).

Among the cultivated plant material, common *S. asper* and *S. graecus* morphotypes always flowered during the first year and subsequently died, regardless of leaf arrangement. In contrast, *S. glaucescens* individuals were either annual or flowered in their second year and then died. Plants of the *S. nymanii* morphotype flowered variably and sparsely over several years, producing rhizomes that repeatedly generated new shoots (Fig. 1i). These observations confirm the accuracy of the life-form assessments in the protologues and the appropriateness of the morphotype assignments.

Populations of the small-floret *S. asper* morphotype produced a high fruit set regardless of the pollination treatment (Table 1; A.CAB[A]: *P* = 0.968; A.MAR[g]: *P* = 0.105), with ISI values near zero. We were unable to obtain sufficient data from the outcrossing treatments to calculate the ISI for the A.PEL[a] population, but the fruit set from ASP = 0.92 suggested an equally near-zero value. Within the large-floret group (*S. nymanii*, *S. glaucescens*, and *S. graecus* morphotypes), fruit set differed significantly among pollination treatments (Table 1; see Supplementary Table S5: *P* ≤ 0.001–0.016). Mean fruit set from autonomous (ASP) and forced (FSP) self-pollination was very low and did not differ in nine populations (*P* = 0.063–0.735; α = 0.025), except for R. MET[a] and R.GRE[g] (*P* = 0.011 and 0.012, respectively). Cross-pollination (MCP) fruit set was always significantly higher than in FSP (*P* ≤ 0.001–0.018; α = 0.025. See Supplementary Table S5). Consequently, the estimated ISI denoted an obligate xenogamous mating system in nine populations (ISI = 0.88–1.00) and a mixed system biased towards obligate xenogamy in two others: R.TER[g] and R.MET[a] (ISI = 0.70 and 0.79). In R.KAS[g], the ISI, exceptionally derived from ASP and MCP, also showed a prevalence of xenogamy. Individual ISI values (excluding R.KAS[g]) showed that 80 out of 93 large-floret plants tested were obligately xenogamous, 10 appeared to have a mixed system (ISI = 0.42–0.78, from G.POR[a], R.TER[g], R.ACH[g], and R.MET[a]), and only 3 were self-fertile (ISI = 0.08–0.20, from G.GAU[g], R.TER[g], and R.MET[a]).

**Table 1:** Variability in sexual system of *Sonchus asper* (L.) Hill populations in the northern Mediterranean Basin, classified according to the assigned morphotype.

Population (morphotype)	*N*	ASP	FSP	MCP	ISI	ISI state
**(Common *S. asper* (L.) Hill)**						
IT: San Pellegrino in Alpe (A.PEL[a])	6	0.92 (0.04)	—	—	—	SC
ES: Villamartín—Las Cabezas (A.CAB[A])^T^	12	0.75 (0.22)	0.77 (0.21)	0.84 (0.20)	0.07 (0.18)	SC
ES: Los Marines—Cortelazor (A.MAR[g])^T^	8	0.88 (0.14)	0.90 (0.09)	0.92 (0.04)	0.03 (0.09)	SC
**(*S. nymanii* Tineo & Guss.)**						
IT: Sicily, Scillato—Caltavuturo (N.SCI[AG])	8	<0.01 (<0.01)	<0.01 (0.01)	0.60 (0.34)	1.00 (<0.01)	SI
IT: Sicily, Alia (N.ALI[AG])	7	0.01 (0.02)	<0.01 <(0.01)	0.42 (0.29)	0.99 (0.01)	SI
**(*S. glaucescens* Jord.)**						
FR: Île de Gaou (G.GAU[g])	10	0.08 (0.16)	0.09 (0.21)	0.80 (0.13)	0.88 (0.29)	SI
FR: Port-Cross (G.POR[a])	8	0.00	0.02 (0.03)	0.41 (0.43)	0.89 (0.06)	SI
**(*S. graecus* E. Weiss)**						
TR: Kastamonu (R.KAS[g])^T^	7	0.01 (0.02)	—	0.66 (0.16)	0.98 (0.04)*	SI
TR: Terme—Ḉraşamba (R.TER[g])	8	0.08 (0.19)	0.14 (0.23)	0.57 (0.26)	0.79 (0.29)	pSI
GR: Achaïa beach (R.ACH[g])	14	0.02 (0.03)	0.05 (0.11)	0.80 (0.19)	0.92 (0.17)	SI
GR: Meteora (R.MET[a])	10	0.02 (0.02)	0.25 (0.21)	0.82 (0.14)	0.70 (0.26)	pSI
GR: Grevena (R.GRE[g])	11	<0.01 (<0.01)	0.04 (0.06)	0.75 (0.25)	0.95 (0.10)	SI
IT: Cesena—Cervia (R.CES[a])	7	0.03 (0.03)	0.02 (0.01)	0.92 (0.06)	0.98 (0.01)	SI
FR: Le Pas de Courtin (R.LAV[AG])	10	<0.01 (0.01)	<0.01 (0.01)	0.91 (0.03)	0.99 (0.01)	SI

We indicate mean population values of fruit set (s.d. in brackets) for the following pollination treatments in the greenhouse: ASP (autonomous self-pollination), FSP (manual forced self-pollination) and MCP (manual multiple individual cross-pollination). We also show mean ISI (index of self-incompatibility) = 1 − (FSP/MCP) and ISI states (Lloyd 1965, Bawa 1974, Raduski et al. 2012): SC (self-compatibility, ISI < 0.2), pSI (partial-self-incompatibility, 0.2 ≤ ISI < 0.8) and SI (self-incompatibility, ISI ≥ 0.8). (*) ISI = 1 − (ASP/MCP). *N*, number of individuals per population studied. ^T^Individuals obtained from transplanted young plants.

### ITS/ETS phylogeny

The aligned ITS region comprised 648 nucleotide sites, and the ETS consisted of 439, yielding a total of 1087 concatenated characters. This dataset included multiple-state polymorphisms at 26 sites: 12 within the ITS and 14 within the ETS regions (see Supplementary Table S6). Two alternative nucleotides were detected at nine of these polymorphic sites, whilst the remaining 17 sites exhibited only a single nucleotide polymorphism. Polymorphic sites were present in all but three populations (i.e. A.AMO[g], Italy, and A.BAD[A], Morocco, of the *S. asper* morphotype, and R.EUR[a], Greece, of the *S. graecus* morphotype). A total of 10 polymorphic sites were detected exclusively in *S. asper* populations, whereas 15 others were unique to the *S. graecus*/*S. nymanii*/*S. glaucescens* group. ML analysis of the concatenated sequences, which included 26 parsimony-informative sites, produced an unrooted tree (Fig. 4) resolving two major clusters within the complex. The first group comprised exclusively accessions of the small-floret *S. asper* morphotype, whereas the second group included all three large-floret morphotypes—*S. nymanii*, *S. graecus*, and *S. glaucescens*—with no exceptions to this pattern being detected. Two well-defined subclusters were recovered within the second group: one included all accessions assigned to *S. nymanii*, and the other included the accessions of *S. graecus* and *S. glaucescens* grouped together. The limited number of informative ITS/ETS characters did not provide sufficient resolution to reveal any further clusters within either of the two groups. In addition, little correspondence between tree topology and leaf arrangement was detected, as both plants with well-developed cauline leaves and plants with a sub-rosulate habit were present across all major clusters in the tree. However, the presence of a vigorous stem with dense, well-developed cauline leaves and no basal leaves during blooming ([A]) was restricted exclusively to the cluster of small-floret plants (the common *S. asper* morphotype).

**Figure 4:**
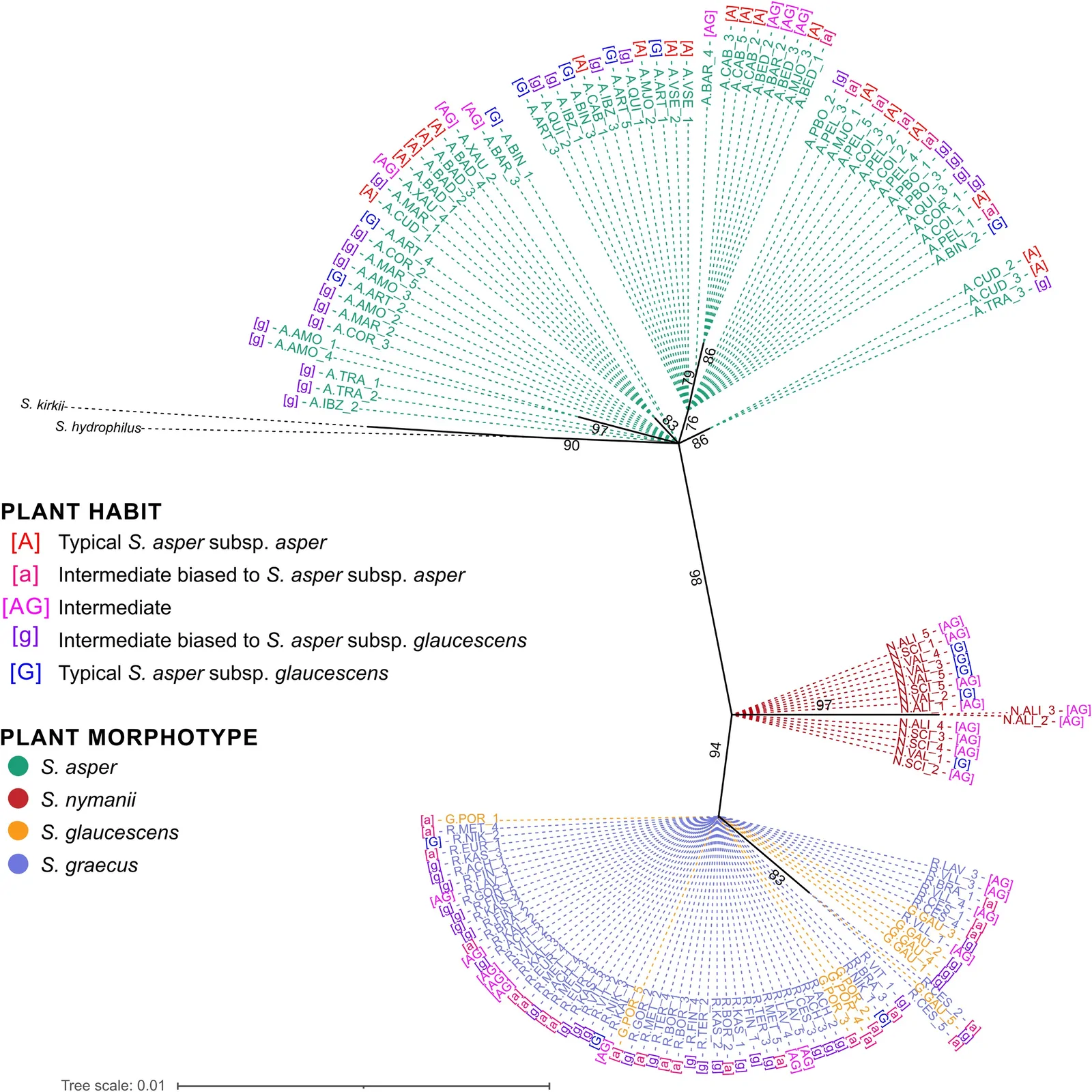
ML phylogenetic tree of the *Sonchus asper* complex in the northern Mediterranean based on nrDNA ITS/ETS sequences. Bootstrap values are shown on the major branches, and the morphotype and leaf arrangement assigned to each sample are indicated by colours and letters, according to indications in the figure. Populations are identified by acronyms according to Supplementary Table S2 and Supplementary Figure S1.

### cpDNA data

The concatenated matrix of cpDNA contained a total of 2265 characters: 826 for *ndhJ–trnL*, 790 for *atpI–atpH*, and 649 for *psbJ–petA*. The ML tree (see Supplementary Figure S2) exhibited low BS values. In contrast to the nuclear ITS/ETS analysis, it did not resolve reciprocal monophyly between the small- and large-floret morphotypes. Although none of the four defined morphotypes was monophyletic in this tree, accessions of *S. nymanii* were never sorted into the same clade as *S. glaucescens* accessions. The topology of the cpDNA phylogenetic tree revealed some additional phylogeographical patterns. For example, all Greek accessions of the *S. graecus* morphotype were placed in one of the two major clades, together with several Turkish materials and some accessions from a single Italian population (R.VLL[AG]) of this same morphotype. Within that same major clade, a moderately supported clade (80% BS) contained all but one accession (N.VAL_4) of the Sicilian endemic morphotype *S. nymanii*, two *S. asper* accessions (A.AMO_1 and A.AMO_4) also from Sicily (Italy), and some *S. graecus* accessions. Meanwhile, most accessions (54 of 62) of the *S. asper* morphotype were placed in the other major clade, distributed across three strongly supported clades (88–94% BS) alongside some Italian and French accessions of the *S. graecus* morphotype. This major clade also contained a separate clade where these accessions mapped together with all but one of the *S. glaucescens* accessions (G.GAU_3) and several accessions of *S. graecus* from Italy and Turkey. No phylogenetic or phylogeographical pattern could be inferred from the distribution of leaf arrangements in the cpDNA phylogenetic tree. Moreover, it is noteworthy that both leaf arrangements are present in virtually all clusters containing four or more accessions.

A total of 20 haplotypes were identified based on the three concatenated regions of cpDNA (see Supplementary Tables S7, S8). Their geographical distribution showed a notable pattern characterized by the following main features (Fig. 5): Haplotypes H1–H5 were primarily distributed across the western and central Mediterranean, occurring in the *S. asper*, *S. glaucescens*, and *S. graecus* morphotypes. None of them was found in the Balkan Peninsula; however, the most common haplotype, H1, was also frequent in the eastern subregion (*S. graecus* from the R.KAS[g], R.TER[g], and R.FIN[g] populations from Turkey), whereas H4 was frequent in the central-northern area. H6 was common among accessions of *S. graecus* from the Balkans, but was infrequently found in *S. asper*, specifically in material from the southern Iberian Peninsula (A.MJO[A]) and Morocco (A.BAD[A]). Furthermore, several haplotypes (H7, H9, H10, H11, H12, H14, H16, H17, H18, H19, and H20) were unique to the Balkan region or the island of Sicily, or shared exclusively between these two areas.

**Figure 5:**
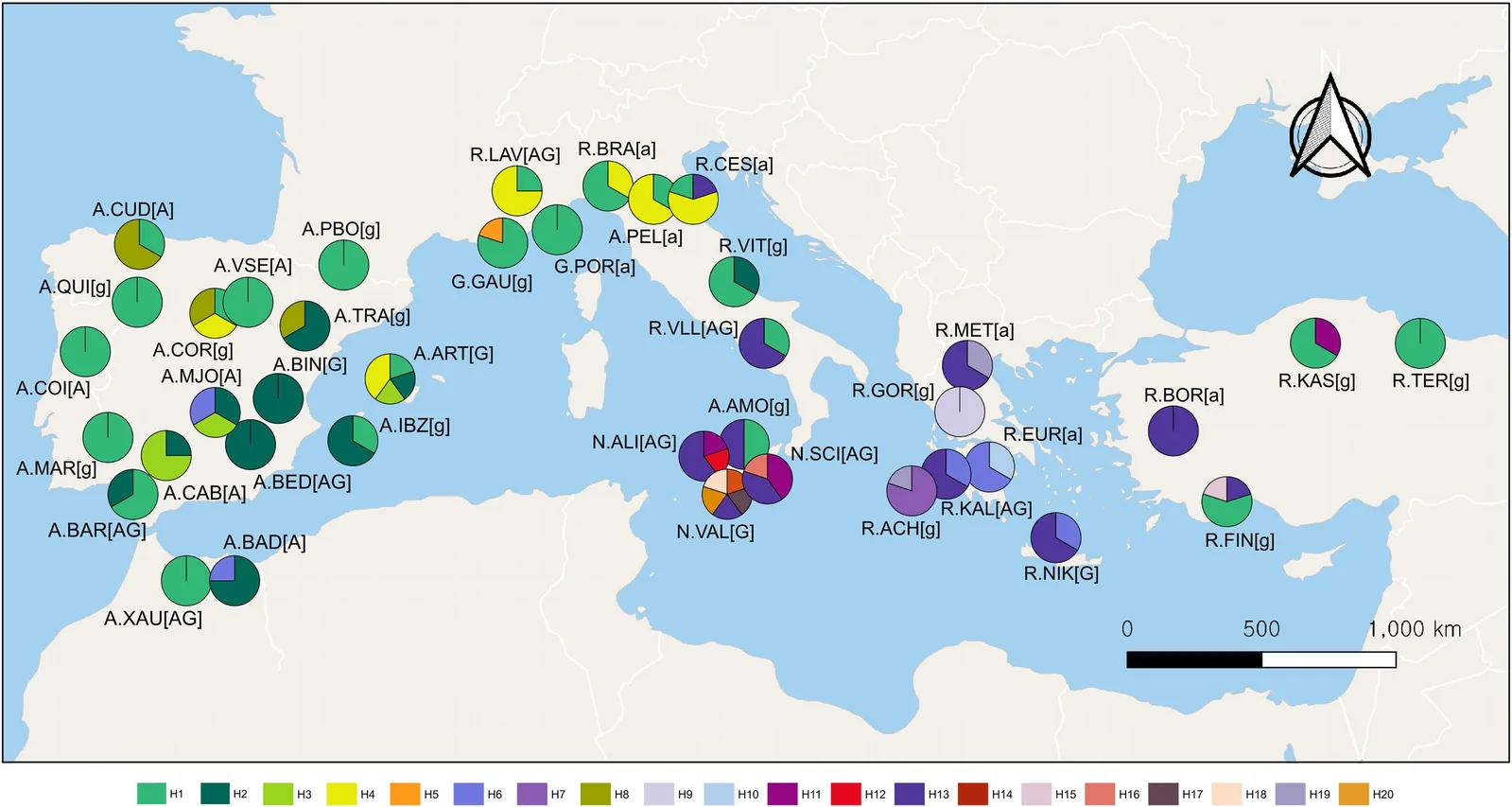
Geographical pattern of haplotype diversity of the cpDNA genes. For each population, a pie chart is shown representing the haplotypes found by colours, using the nomenclature indicated in the figure. Populations are identified by acronyms formed according to Supplementary Table S2 and Supplementary Figure S1.

The TCS network (Fig. 6) showed that most haplotypes were connected by a single mutational step, suggesting a recent origin. A total of 18 haplotypes were identified in large-floret morphotypes (2 in *S. glaucescens*, 8 in *S. nymanii*, and 11 in *S. graecus*), whereas seven haplotypes were found in the small-floret *S. asper* morphotype (see Supplementary Tables S7 and S8). The number of morphotype-specific haplotypes ranged from one in *S. glaucescens* to six in *S. nymanii*. The most common haplotype, H1, was shared among 52 accessions of three morphotypes: *S. glaucescens*, *S. graecus*, and *S. asper*. The second most common haplotype, H13, was shared among 21 accessions of the *S. nymanii* and *S. graecus* morphotypes, alongside two rare accessions of *S. asper* from Sicily (A.AMO[g]). The remaining haplotypes were present in 1 to 16 accessions of one or, less frequently, two morphotypes (Fig. 6).

**Figure 6:**
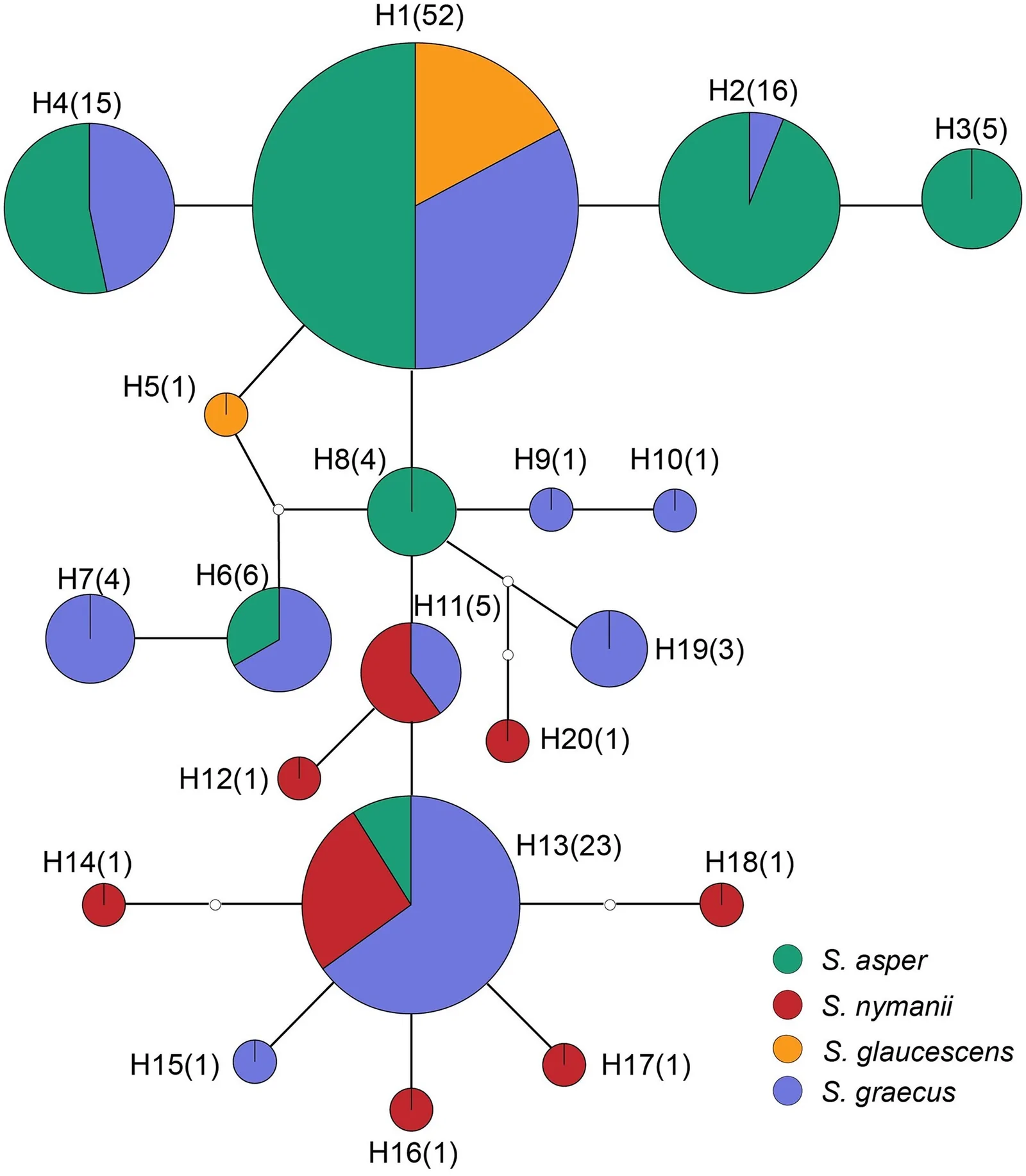
Haplotype parsimony network of the cpDNA genes in the *Sonchus asper* complex of the northern Mediterranean. Haplotype variants are represented by numbers preceded by ‘H’: H1–H20. The number of samples analysed presenting each haplotype is also indicated in parentheses, as well as the morphotypes in which they are present, represented by colours. The small circles on each link represent additional steps between haplotypes (see Supplementary Tables S7, S8).

### AFLP data

The six AFLP primer combinations generated 587 unambiguously scorable DNA fragments—of which 586 were polymorphic—from 240 individuals across 24 populations. In the individual-level PCoA (Fig. 7a), the first and second axes explained 63.0% and 14.3% of the total variation, respectively. Axis 1 clearly separated the populations into the small-floret (A.CAB[A], A.ART[G], A.XAU[AG], A.BAD[A], A.PEL[a]: *S. asper* morphotype) and large-floret morphotypes (the remaining populations: *S. nymanii*, *S. glaucescens*, and *S. graecus* morphotypes), although some individuals from A.PEL[a] were genetically close to the large-floret group and some individuals from R.TER[g] were genetically close to the small-floret morphotype. Axis 2 revealed that the populations of large-floret morphotypes formed three distinct groups: those of the *S. nymanii* morphotype (N.SCI[AG], N.VAL[G], and N.ALI[AG]) and *S. graecus* morphotype (R.KAS[g], R.TER[g], R.BOR[a], R.FIN[g], R.NIK[G], R.EUR[a], R.KAL[AG], R.ACH[g], R.GOR[g], R.MET[a], R.GRE[g], R.VIT[g], R.CES[a], R.VLL[AG], R.BRA[a], and R.LAV[AG]) were the most widely separated from each other, whereas the populations of the *S. glaucescens* morphotype (G.GAU[G] and G.POR[a]) occupied an intermediate position. The PCoA conducted at the population level (Fig. 7b) yielded similar results and highlighted the genetic differentiation between the small- and large-floret groups, as well as the divergence among the populations of the morphotypes *S. nymanii*, *S. glaucescens*, and *S. graecus*. No grouping pattern could be inferred from leaf arrangement.

**Figure 7:**
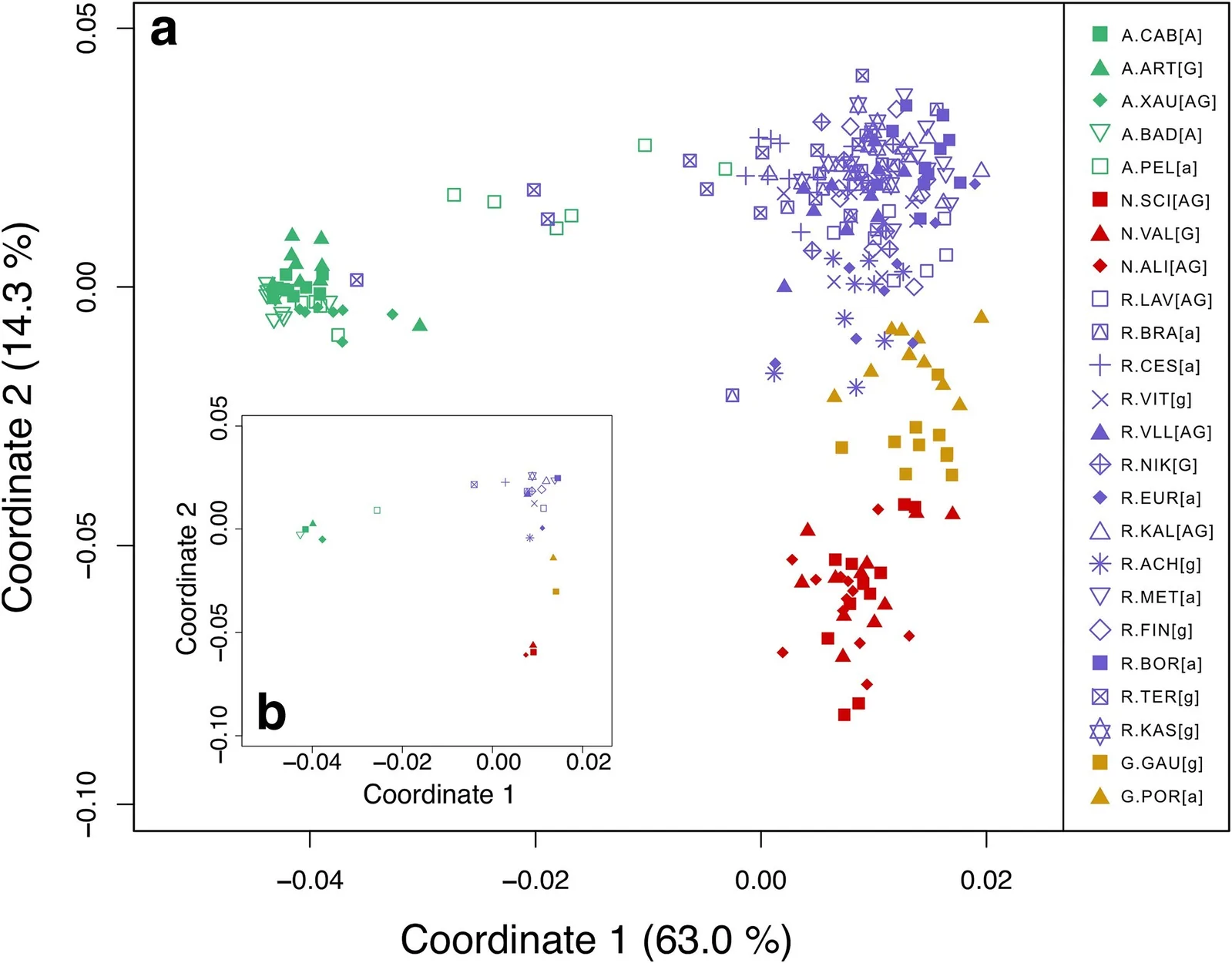
(a) Principal coordinate analysis (PCoA) based on a matrix of Nei and Lei’s similarities among 240 individuals of *Sonchus asper* taxonomic complex from northern Mediterranean basin analysed by AFLP fingerprinting (for details on population acronyms see Supplementary Table S2, Supplementary Figure S1). The percentage of variability explained by the first two coordinates amounts to 77.3%. (b) PCoA based on Nei and Lei’s distance at the population level.

Bayesian clustering analyses with STRUCTURE (Fig. 8) were generally consistent with the PCoA results (Fig. 7), showing that the most informative representation of overall genetic structure was achieved at *K* = 2 (*ΔK* = 211. See Supplementary Figure S3). The first cluster (small-floret morph) was homogeneous and consisted exclusively of individuals of the common *S. asper* morphotype, whereas the second cluster (large-floret morph) comprised individuals of the *S. nymanii*, *S. graecus*, and *S. glaucescens* morphotypes. A second peak at *ΔK* = 80 was obtained for *K* = 3, which separated the populations into three distinct clusters: *S. asper* morphotype, *S. nymanii* morphotype, and the *S. graecus/S. glaucescens* morphotypes; however, in the last cluster, some populations exhibited an extensive degree of admixture with the *S. nymanii* (R.EUR[a], R.ACH[g], G.GAU[g], and G.POR[a]) or *S. asper* (R.TER[g]) groups. A further minor peak of *ΔK* = 11 was obtained for *K* = 5, which separated the populations into five distinct clusters: one for each of the *S. asper*, *S. nymanii*, *S. glaucescens* and *S. graecus* morphotypes, alongside one group comprising R.EUR[a] and R.ACH[g]. Conversely, the Bayesian clustering conducted with BAPS (Fig. 8b) resulted in an optimal partition of four clusters, with a log-marginal likelihood of −52 086. This was highly congruent with the results obtained by STRUCTURE at *K* = 5, with the exception of the assignment of R.EUR[a] and R.ACH[g] to a separate group.

**Figure 8:**
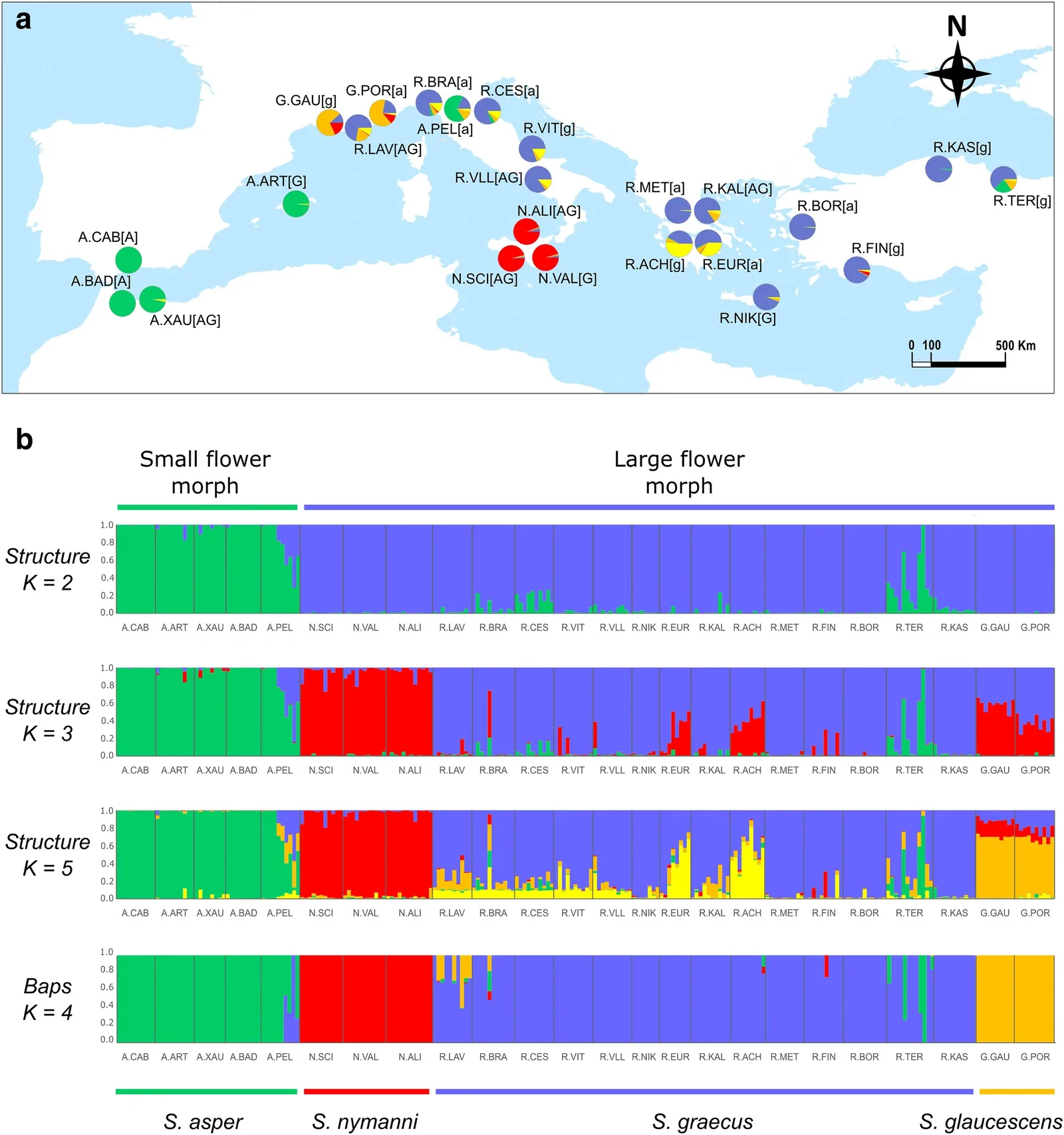
(a) Localities of the 240 sampled plants of *Sonchus asper* complex studied from 24 populations by AFLP fingerprinting (for details on population acronyms see Supplementary Table S2, Supplementary Figure S1). Pie charts indicate the global score of DNA fragments for each population. (b) Population structure of *S. asper* complex in the northern Mediterranean inferred by Bayesian clustering of the AFLP data by STRUCTURE and BAPS (*K* = 2–5).

A non-hierarchical AMOVA (Table 2) revealed that most of the overall genetic variation was explained by the within-population component (67.57%; *F*_ST_ = 0.324). A nested AMOVA (i.e. grouping small-floret vs. large-floret morphotypes) attributed the highest global variation (*F*_ST_ = 0.437, *P* < 0.001) to differences between the two floret morphs (‘small vs. large’). This global variation slightly decreased when considering three groups within *S. asper* (*S. asper* vs. *S. nymanii* vs. [*S. graecus* + *S. glaucescens*]) (*F*_ST_ = 0.405, *P* < 0.001). Global variation did not increase when considering either the nesting pattern of the four morphotypes (*S. asper* vs. *S. nymanii* vs. *S. graecus* vs. *S. glaucescens* morphotypes; *F*_ST_  _=_ 0.392; *P* < 0.001) or the genetic clusters retrieved with STRUCTURE at *K* = 5 (*F*_ST_  _=_ 0.378; *P* < 0.001).

**Table 2:** Results of AMOVA of AFLP data (squared Euclidean distance) from the 24 populations of *Sonchus asper* complex analysed (Fig. 8).

Grouping	*N*	Source of variation	d. f.	Variance components	Percentage of variance	Fixation Index [95% CI]
**a**. Whole sampling	24					*F*_ST_ = 0.324 [0.287–0.342]
		Among populations	23	10.22	32.43	
		Within populations	216	21.30	67.57	
**b**. Small floret morph vs. large floret morph morphotypes [common *S. asper*] [*S. nymanii* + *S. glaucescens + S. graecus*]	2					*F*_ST_ = 0.437 [0.375 −0.466]
		Among groups	1	9.42	24.88	*F*_CT_ = 0.249 [0.169–0.287]
		Among populations	22	7.13	18.43	*F*_SC_ = 0.251 [0.223–0.265]
		Within populations	216	21.30	56.28	
**c**. *S. asper* vs. *S. nymanii* vs. [*S. glaucescens + S. graecus*]	3					*F*_ST_ = 0.405 [0.359–0.428]
		Among groups	2	9.02	25.18	*F*_CT_ = 0.252 [0.193–0.281]
		Among populations	21	5.49	15.34	*F*_SC_ = 0.205 [0.176–0.220]
		Within populations	216	21.30	59.48	
**d.** *S. asper* vs. *S. nymanii* vs. *S. glaucescens* vs. *S. graecus*	4					*F*_ST_ = 0.392 [0.347–0.413]
		Among groups	4	9.35	26.70	*F*_CT_ = 0.267 [0.214–0.292]
		Among populations	19	4.37	12.49	*F*_SC_ = 0.170 [0.144–0.184]
		Within populations	216	21.30	60.81	
**e**. Grouping following STRUCUTRE clustering (*K* = 5) *S. asper* vs. *S. nymanii* vs. *S. glaucescens* vs. [*S. graecus—*(R.TER, R.ACH)] vs. [R.TER, R.ACH]	5					*F*_ST_ = 0.378 [0.336–0.398]
		Among groups	4	9.01	26.32	*F*_CT_ = 0.263 [0.218–0.286]
		Among populations	19	3.91	11.43	*F*_SC_ = 0.155 [0.127–0.169]
		Within populations	216	21.30	62.24	

d. f. degrees of freedom; general fixation index (*F*_ST_), fixation index for the region (*F*_CT_), and population within region (*F*_SC_) level are shown, in brackets the 0.95 confidence interval. Populations analysed are marked by GS in ‘Methodologies’ column in Supplementary Table S2 (see Supporting Information).

## Discussion

Our analysis reveals unexpected variability in floral traits, reproductive behaviour, and life forms within the currently accepted circumscription of *Sonchus asper* in the northern Mediterranean. First, two groups of plants can be separated by floret and flower head size (Fig. 2), corresponding to distinct mating systems (Table 1). Second, although annual life cycles dominate within the complex, biennial and rhizomatous perennial populations occur among large-floret plants (Fig. 1). These ecological differences consistently align with the ITS/ETS phylogenetic topology, which resolves three strongly supported clusters characterized by mating system and life cycle (Fig. 4). This variability—reflected in 19th-century descriptions but currently overlooked—implies consistent differences in ecological behaviour and evolutionary trends within what is currently considered a single species, necessitating a taxonomic reconsideration (Ornduff 1969, Anderson et al. 2002, Conix 2018). Furthermore, our sampling indicates a significant geographical pattern (Fig. 3). Western Mediterranean populations consistently exhibit small flowers and an annual cycle (*S. asper* morphotype), though they occur sparsely elsewhere. Conversely, most central and eastern northern Mediterranean populations display larger florets and showier flower heads. Among these, the annual cycle remains common (*S. graecus* morphotype), but prominent deviations appear in central Sicily and certain coastal areas of south-eastern France, where rhizomatous perennial (*S. nymanii* morphotype) and biennial (*S. glaucescens* morphotype) populations are confirmed.

Crucially, the phylogenetic tree topology and genetic variability do not support the division of *S. asper* into two subspecies based on leaf arrangement. Furthermore, assigning a biennial cycle to sub-rosulate plants (Boulos 1973, 1976) lacks experimental support, undermining this trait’s taxonomic value. The geographical pattern of leaf arrangement has little evolutionary significance and does not seem to reflect functional differences between the two groups of plants defined by floret size (see Supplementary Table S4).

### Divergence in mating system and persistence mechanisms

As expected, small-floret plants of *S. asper* exhibit a high capacity for self-fertilization (Lewin 1948, Hsieh et al. 1972) with ISI values close to 0 (Table 1; Lloyd 1965, Raduski et al. 2012) and short anthers with low pollen content (fewer than 350 grains per floret; Mejías 1992), thereby exhibiting a clear selfing syndrome (Sicard and Lenhard 2011). Mejías (1992) also reported high self-fertilization in common *S. asper* plants, including both subsp. *asper* and subsp. *glaucescens*. In contrast, the large-floret group exhibits a markedly xenogamous mating system, with ISI values near 1 (0.70–1.00) and higher pollen contents (∼2000 grains per floret; unpubl. data). These divergences reflect well-differentiated reproductive strategies (e.g. Lloyd 1965, Wyatt 1984, Bernacchi and Tanksley 1997, Guo et al. 2009, Soto-Trejo et al. 2013, Freyman and Höhna 2019) rather than gradual shifts, as evidenced by the bimodal distribution of ligule and anther sizes (Fig. 2). Furthermore, the three xenogamous morphotypes differ in their persistence mechanisms, which likely involve distinct demographic features and requirements associated with the reproductive phase (Silvertown 1983, Vallejo-Marín et al. 2010, Thrall et al. 2014, Barrett and Harder 2017, Friedman 2020, Sun et al. 2022). Conversely, the self-compatible group consists exclusively of monocarpic annuals, a pattern that is common among selfers (Barrett and Harder 2017, Friedman 2020, Ferrer et al. 2025).

Obligate xenogamy is likely driven by a strong sporophytic self-incompatibility reaction, which is typical of Asteraceae (Hughes and Babcock 1950, Allen and Hiscock 2007, Brennan et al. 2011). This hypothesis is supported by the low fruit set under FSP (Table 1) and uniform patterns of pollen presentation and stigma accessibility across groups, indicating no significant herkogamy or dichogamy effects between them. Nevertheless, some reproductive variability occurs, as is common within the family (e.g. Brennan et al. 2005, Ortiz et al. 2006, Scheffknecht et al. 2007, Koseva et al. 2017), sometimes biasing population-level ISI towards the margin of the self-incompatibility values (0.70–0.79; Table 1). The existence of two divergent groups with well-defined boundaries—governed mainly by self-incompatibility (Raduski et al. 2012)—aligns with the influential hypothesis by Lande and Schemske (1985) regarding the predominance of selfing or outcrossing as the two stable states in a population's mating system. However, factors stabilizing mixed strategies or modulating incompatibility responses might operate in populations with skewed mating systems (Barrett 2003, Goodwillie et al. 2005).

### Phylogenetic and phylogeographic patterns

The ITS/ETS phylogenetic reconstruction (Fig. 4) resolved all variation into two major clades corresponding to mating systems, with no topological inconsistencies. The earliest-diverging clade comprised the common *S. asper* morphotype representatives: annual weeds characterized by small florets and a high selfing ability. The remaining cluster included the three primarily xenogamous, large-floret morphotypes. Similarly, AFLP analysis showed that the most informative genetic structure occurred at *K* = 2 (Table 2, Fig. 8b; see Supplementary Figure S3), separating small- from large-floret plants, which strongly suggests that mating system differentiation occurred early during diversification. The ITS/ETS topology revealed no further clustering among small-floret plants; moreover, although all variation in leaf arrangement is present within this clade, no apparent phylogenetic sorting was detected. This, together with the annual cycle observed in all experimental populations, demonstrates that the current criterion for subdividing *S. asper* into two subspecies based on leaf arrangement lacks solid biological and phylogenetic support, and that this trait cannot serve as a surrogate for annual/biennial life cycle variation in this case. Meanwhile, within the large-floret xenogamous clade, both ITS/ETS and AFLP markers consistently separated the rhizomatous perennial *S. nymanii* accessions from the remaining two large-floret morphotypes: *S. glaucescens* (frequently biennial and highly prickly) and *S. graecus* (strictly annual). However, BAPS clustering also resolved distinct profiles despite a notable geographical overlap between the *S. glaucescens* and *S. graecus* morphotypes (Fig. 8b); thus, the divergence between these two morphotypes can be viewed as the result of a recent eco-regional adaptation that generated two cohesive population systems.

Haplotype diversity in cpDNA markers does not consistently correspond to mating systems and life forms; nonetheless, its lineage-sorting patterns, together with ITS/ETS and AFLP data, allow us to formulate several evolutionary hypotheses for the group. The *S. nymanii* morphotype exhibits high diversity in cpDNA haplotypes and maximum specificity despite its narrow central Sicilian range (Fig. 6; see Supplementary Tables S7, S8), which suggests an ancient origin, as do the nuclear markers. Since the rhizomatous habit is a widespread trait in three of the eight proposed clades for *Sonchus* s. l. (Kim et al. 2007), it likely represents the retention of an ancestral trait rather than an evolutionary novelty. The *S. glaucescens*/S. *graecus* lineage displays the greatest cpDNA diversity within the complex (see Supplementary Tables S4, S5), yet surprisingly, its haplotype profile resembles that of the self-fertile clade more than the other xenogamous clade, thereby supporting an ancient, independent origin for both xenogamous groups. This cpDNA variability is mainly exhibited by the widespread *S. graecus* morphotype, whilst *S. glaucescens* shows remarkable haplotypic homogeneity, again pointing to a recent, derived origin. Unexpectedly, cpDNA haplotype diversity also reveals compelling phylogeographic patterns. For example, the Balkan *S. graecus* populations exhibit highly differentiated profiles, as all five private haplotypes within this morphotype are restricted to this region, suggesting historical isolation. Similar phylogeographical breaks associated with Quaternary glacial refugia have been documented in this region (Španiel and Rešetnik 2022). Other haplotypes also display geographically biased distributions, reflecting, for instance, a west–east differentiation across the Iberian Peninsula.

Despite the clear segregation into clades, cpDNA haplotype diversity (Fig. 5) and AFLP profiles (Figs 7 and 8) reveal a substantial level of admixture—both between large-floret morphotypes (e.g. R.LAV[AG]) and, more interestingly, between *S. asper* and other morphotypes (e.g. A.PEL[a], A. AMO[g] and R.TER[g]). Intrinsic post-zygotic isolation mechanisms (genic and chromosomal barriers) likely have reduced effectiveness within the complex; indeed, no differences in chromosome number (*2n* = 18) and little variation in chromosome morphology occur between *S. asper* and *S. nymanii* morphotypes (Costales-Maestre et al. 2023), nor among the remaining morphotypes (unpubl. data). Pre-zygotic barriers might be crucial for maintaining this biodiversity (Rieseberg and Willis 2007, Widmer et al. 2009, Sobel et al. 2010), with historical–biogeographical processes likely driving current patterns of variability (Sobel et al. 2010, Baack et al. 2015, Tang et al. 2022). However, ecological interactions should not be underestimated (Sicard and Lenhard 2011, Barrett and Harder 2017, Orsucci and Sicard 2021). First, obligate outcrossers have drastically different pollination requirements compared to selfers. Second, the two endemic morphotypes occupy distinct ecological niches (Rieseberg and Willis 2007, Widmer et al. 2009, Sobel et al. 2010, Shang et al. 2020): inland croplands in Sicily for *S. nymanii*, and a narrow coastal habitat in France for *S. glaucescens*. Assessing hybridization barriers and ecological niche factors, together with characterizing morphotype diversity across the southern Mediterranean Basin, will provide deeper insights into the evolutionary history of this taxonomic complex and the mechanisms maintaining its diversity.

### Taxonomic and nomenclatural proposals

The phenotypic variability described here, together with the molecular data, scarcely supports retaining *S. asper* as a single species. From both ontological (conceptual) and epistemological (operational) perspectives, distinct lineages exhibiting such disparate ecological and evolutionary trajectories in mating systems and life forms within a single species are difficult to reconcile (Simpson 1951, van Valen 1976, Wiley and Mayden 2000, Sites and Marshall 2004, de Queiroz 2007, Choi 2016, Zachos 2016). Remarkably, these divergences only surfaced upon re-examination of ancient protologues, with a focus on potential indicators of differential ecological performance. Owing to the difficulty of discerning such distinctive traits in herbarium specimens, they had hitherto been overlooked; however, our subsequent testing has confirmed that these are functional traits of evolutionary significance in this species complex. Crucially, bringing this unaccounted diversity to light does not reflect cryptic speciation processes, but rather is an artefact of over-reliance on herbarium material—leading to what could be termed ‘cryptic herbarium taxa’.

According to the typification performed by Boulos (1998), the name *S. asper* (L.) Hill (UPS-BURSER 6:14) should be applied exclusively to annual plants with small florets. A few remaining anthers on the designated specimen allow their length to be estimated at ∼1 mm, placing this specimen within the group of self-fertile plants with low pollen content (Table 1, Fig. 2). Within the large-floret group, we propose recognizing two well-characterized clades at the species level: the rhizomatous perennial *S. nymanii* Tineo & Guss., and the short-lived *S. glaucescens* Jord. Furthermore, within the latter species, two cohesive population systems should be recognized as subspecies, given their lower genetic divergence (Fig. 4) and minor evolutionary singularity (Patten 2015, Reydon and Kunz 2021). This involves accepting paraphyletic taxa at lower hierarchical levels (Carnicero et al. 2019, Míguez et al. 2021).

Based on these criteria, Box 1 presents a proposed taxonomic treatment for the plants currently treated as *S. asper* in the northern Mediterranean, pending a more comprehensive morphometric analysis of this complex. The inherent taxonomic variability recognized here significantly enhances our knowledge of biodiversity within the genus *Sonchus* in the area. Furthermore, it reinforces the hypothesis that the clade containing *S. asper* originated and primarily diversified within the Mediterranean basin (Kim et al. 2007).

Box 1. Taxonomic treatment for the taxa within *Sonchus asper* s. l. in the northern Mediterranean

Key to the taxa:

1a. Outer anthers 0.5–1.9 mm; ligules of the outermost florets in the head less than 6.5 mm long, shorter than the tube **1. *S. asper***

1b. Outer anthers 2.0–4.4 mm; ligules of the outermost florets in the head more than 6.5 mm long, longer than the tube 2

2a. Rhizomatous perennials; achenes with double lateral ribs; leaf margins soft **2. *S. nymanii***

2b. Annuals or biennials; achenes with single or double lateral ribs; leaf margins soft to strongly spiny 3

3a. Leaves rigid and coriaceous, with strongly spiny margins, pinnatipartite, the largest with more than 12 lateral lobes; plants biennial or annual **3a. *S. glaucescens* subsp. g*laucescens***

3b. Leaves soft, with weakly spiny margins, pinnatifid to pinnatipartite or rarely undivided, with no more than 12 lateral lobes; plants strictly annual **3b. *S. glaucescens* subsp. *graecus***

**1. *S. asper*** (L.) Hill, Herb. Brit. 1: 47 (1769)

*S. oleraceus* var. *asper* L., Sp. Pl.: 794 (1753) [basion.]

*S. asper* subsp. *glaucescens sensu* Boulos, non *S. glaucescens* Jord., Observ. Pl. Nouv. 5: 75, t. 5 (1847), non *S. asper* subsp. *glaucescens* (Jord.) Ball in J. Linn. Soc., Bot. 16: 548 (1878)

*S. asper* subsp. *nymanii sensu* Hegi, non *S. nymanii* Tineo & Guss. in Guss., Fl. Sicul. Syn. 2: 860 (1844).

Annuals, (5–)18–150 cm tall. Stems solitary or with up to 6 erect-patent basal branches. Leaves undivided, or pinnatifid to pinnatipartite with up to 12 lateral lobes; margins spiny with teeth 1.5–9.7 mm long, very rarely soft, sometimes fleshy and coriaceous. Flower heads 10.8–29.5 mm in diameter during anthesis. Florets yellow, often pale yellow; outermost florets with ligules 3.1–6.7 × 0.6–1.2 mm and tubes 3.5–7.9 mm; anthers 0.5–2.1 mm. Achenes oblanceolate or elliptic, the inner ones sometimes oblong, 1.9–3.7 × 0.6–1.7 mm, with 3 single ribs; wings 0.05–0.35 mm wide.

*Habitat*: Cultivated and uncultivated fields, paths, roadsides, and grasslands in disturbed environments. Prefers moist soils. Elevation: 0–1500 m a.s.l.

*Known distribution*: Cosmopolitan; common in the western Mediterranean region.

*Taxonomic notes*: To the best of our knowledge, this is the species within the complex that displays a worldwide distribution, whereas the remaining taxa appear to be restricted to the Mediterranean region. Comprehensive global sampling is desirable to test this hypothesis.

**2. *S. nymanii*** Tineo & Guss. in Guss., Fl. Sicul. Syn. 2: 860 (1844)

non *S. asper* subsp. *nymanii* (Tineo & Guss.) Hegi, Ill. Fl. Mitt.-Eur. 6(2): 1110 (1928)

Rhizomatous perennials with erect, commonly unbranched shoots 48–94 cm long. Leaves pinnatifid to pinnatisect with 2–14(−20) lateral pinnae and soft margins, bearing teeth 1.0–2.5(−4.1) mm long. Flower heads 15.4–44.3 mm in diameter during anthesis. Florets bright yellow; outermost florets with ligules 9.5–12.7 × 1.5–2.3 mm and tubes 7.0–10.1 mm; anthers (2.2–)2.7–4.8 mm. Achenes oblanceolate 2.3–3.4 × 0.7–1.2 mm, generally with a single central rib and 2 double lateral ribs; wings 0.02–0.16 mm wide.

*Habitat*: Cultivated fields, abandoned farmlands, and lower slopes. Damp clay soils. Elevation: 400–900 m a.s.l.

*Known distribution*: Central Sicily (Italy).

*Taxonomic notes*: It is often misidentified as the polyploid *S. arvensis* (*2n* = 36, 54; Mejías and Andrés 2004), which is also a rhizomatous perennial, but whose achenes have rounded, unwinged margins.

**3. *S. glaucescens*** Jord., Observ. Pl. Nouv. 5: 75, t. 5 (1847)

*S. asper* subsp. *glaucescens* (Jord.) Ball in J. Linn. Soc., Bot. 16: 548 (1878)

non *S. asper* subsp. *glaucescens sensu* Boulos

Annuals or biennials. Stems solitary or with up to 8 erect-patent basal branches. Achenes oblanceolate or elliptic, the inner ones sometimes oblong, with 3 single ribs, or with a single central rib and 1 or 2 double lateral ribs.

**3a.** subsp. ***glaucescens*** Jord., Observ. Pl. Nouv. 5: 75, t. 5 (1847)

Often biennials, 29–100 cm tall. Stems solitary. Leaves mostly pinnatipartite, with up to 24 lateral lobes, rigid, coriaceous, and somewhat fleshy; strongly prickly margins bearing teeth 3.9–5.3 mm long. Flower heads 21.8–49.0 mm in diameter during anthesis. Florets bright yellow, sometimes orange-yellow, outermost florets with ligules 8.0–12.2 × (0.9–)1.2–2.6 mm and tubes 5.8–8.1 mm; anthers 2.2–4.6 mm. Achenes 2.5–3.1 × 1.0–1.4 mm, with wings 0.1–0.3 mm wide.

*Habitat*: Coastal rocky areas. Loose soils.

*Elevation*: 0–180(−506) m a.s.l.

*Known distribution*: South-eastern France: coast of the Provence-Alpes-Côte d’Azur region and the Hyères Islands.

**3b.** subsp. ***graecus*** (Weiss) Mejías & S.-Ch. Kim, **comb. nov.**

*S. graecus* Reut. ex Weiss in Verh. K.K. Zool.-Bot. Ges. Wien 19:45 (1869) [basion.]

Annuals, 13–210 cm tall. Stems solitary or with up to 8 erect-patent basal branches. Leaves usually pinnatifid to pinnatipartite with up to 12 lateral lobes, sometimes undivided; soft margins bearing teeth 1.0–4.6 mm long. Flower heads 15.4–44.3 mm in diameter during anthesis. Florets yellow or bright yellow; outermost florets with ligules (5.5–)6.6–13.1(16.0) × 0.8–2.4 mm and tubes 5.5–8.8 mm; anthers 2.0–4.7 mm. Achenes 1.8–3.5 × 0.8–1.9 mm, with wings 0.05–0.4 mm wide.

*Habitat*: Cultivated and uncultivated fields, paths and road margins, and grasslands in disturbed environments. Prefers moist soils. Elevation: 0–1100 m a.s.l.

*Known distribution*: Central and eastern northern Mediterranean, from south-eastern France to Turkey and Bulgaria. Ruderal/weedy behaviour like that of *S. asper* (L.) Hill, which appears to be rather rare and scarce within the distribution range of this taxon.

### A functional/genealogical framework for species delimitation

We show that analysing functional traits and evaluating how their performance fits into phylogenetic relationships can be useful in unravelling evolutionary histories, thereby facilitating taxonomic judgements (Spalink et al. 2018, Hoffmann 2019). The taxonomic utility of functional traits has recently gained recognition (Mlambo 2014, Moore et al. 2018, Hidalgo-Triana and Pérez-Latorre 2019, Wang et al. 2020), with authors emphasizing their importance even in optimizing herbarium-based research (Heberling 2022, Shi et al. 2025). This requires identifying true functional traits as surrogates for species performance within the studied taxonomic group (Violle and Jiang 2009, Mlambo 2014). In our study, the breeding system (and its associated characters) and life form proved to be functional traits of genuine biological significance, though not necessarily of universal application (see Kalinkina et al. 2020). The methodological framework of this approach is inherent in several species concepts (e.g. Simpson 1951, van Valen 1976, Templeton 1989, Baum and Shaw 1995, de Queiroz 1998, 2005). We believe that orienting taxonomic practice towards traits that differentiate ecological performance lends robustness and stability to taxonomic proposals. Furthermore, this key ecological concept links systematics and ecology, adding evolutionary value to biodiversity analysis. Its prominent role in niche assessment for understanding plant community assembly (Violle and Jiang 2009, Escudero and Valladares 2016, E-Vojtkó et al. 2020), alongside the trend of incorporating functional diversity into conservation (Cadotte et al. 2011, de Oliveira et al. 2022), makes integrating functional traits into taxonomy essential.

## Supplementary Material

plag040_Supplementary_Data

## Data Availability

The data underlying this article is available in https://doi.org/10.12795/11441/188982.
