# Supplementary material for: Functional traits reveal unaccounted diversity within Sonchus asper (L.) Hill (Asteraceae) in the northern Mediterranean and enable the reinstatement of ancient taxa
Source: AoB Plants. 2026 Sep 2;18(5):plag040. doi: 10.1093/aobpla/plag040 (PMC13626247; doi:10.1093/aobpla/plag040)
Supplement: Supplementary file 1 [file plag040_supplementary_data.zip › AoBPlants-SupplementaryAsper.North-Med.pdf]

Supplementary Table S1.- Information from a Web of Science query on the use of the concept of functional traits in studies of plant taxonomy and systematics. We performed two main searches (Subject Queries) to detect publications that use this concept in both systematics (systemat*) or taxonomy (taxonom*) studies. Among all the references provided by the Web of Science (General), we selected the results from the category "Plant Science" and reviewed the content of the abstract and/or the entire content to select those that involve systematic or taxonomic objectives (Systematic approach and Taxonomic approach, respectively). We indicate the entire number of references provided, and the number of these references of systematic/taxonomic relevance, detailing the year or period of years of publication ([years]). We also categorize the aims of the latter references as Classification (identifying taxa, groups of taxa or lineages) or Evolutionary (to explain the evolution and diversification of taxonomic groups). The 19 references with systematic/taxonomic relevance obtained throughout the query are listed below.

	**Subject Queries**					
	"**functional trait***"(All Fields) + **systemat***(Topic): **453** results			"**functional trait***"(All Fields) + **taxonom***(Topic): **2,469** results		
	Category: **Plant Science**			Category: **Plant Science**		
	General	(Systematic approach)		General	(Taxonomy approach)	
		**Classification**	**Evolutionary**		**Classification**	**Evolutionary**
Year of first result	1999	2006	2018	1991	2009	2018
Results [years]	2 [1999] +<br>4 [2000-2009] +<br>32 [2010-2019] +<br>62 [2020-2025]:	1[2006] +<br>2 [2019] +<br>3[2020-2025]:	1 [2018] +<br>4 [2020-2025]:	2 [1991-1999] +<br>6 [2000-2009] +<br>119 [2010-2019] +<br>216 [2020-2025]:	1 [2009] +<br>3 [2019] +<br>6 [2020-2025]:	1 [2018] +<br>1 [2023]:
**Total results**	100	**6**	**5**	343	**10**	**2**

Results of systematic/taxonomic relevance obtained throughout the entire query (bibliographic references):

***Borges LM, Pastore JFB, Souza AFC, Rubens Pirani J, Simon MF. 2023**. Let there be clades: phylogenetics of *Mimosa* series *Pachycarpae* and *Setosae* (Fabaceae) improves the infrageneric classification of the genus. *Botanical Journal of the Linnean Society* 201: 61–79 (S, T). ***Chen Y, Chen J, Xue B, Yang B, Thomas DC, Saunders RMK. 2025**. Gynoecial diversity in the Annonaceae and the evolution of functional traits that overcome the limitations of apocarpy. *Journal of Systematics and Evolution* 63: 495–509 (S). ***Gibbs AK, Udovicic F, Drinnan AN, Ladiges PY. 2009**. Phylogeny and classification of *Eucalyptus* subgenus *Eudesmia* (Myrtaceae) based on nuclear ribosomal DNA, chloroplast DNA and morphology *Australian Systematic Botany* 22 :158–179 (T). ***Hidalgo-Triana N, Pérez-Latorre A. 2019**. A study of functional traits reveals serpentinomorphoses and new taxa in populations of Mediterranean *Genista* (Fabaceae). Phytotaxa. 394: 244-256 (T). ***Hoffmann MT. 2019**. To the roots of *Carex*: Unexpected anatomical and functional diversity. *Systematic Botany* 44: 26-31 (S). ***Kalinkina VA, Mikhaylova YV, Kislov DE. 2020**. Diversity and taxonomy of the *Trifolium lupinaster* polymorphic complex in Eastern Europe and Asia. *Flora* 267: 151597 (T). ***Khan R, Hill RS, Liu J, Biffin E. 2023**. Diversity, distribution, systematics and conservation status of Podocarpaceae. *Plants-Basel* 12: 1171 (S). ***Luizi-Ponzo AP, Silva-e-Costa JC. 2019**. Complex sporoderm structure in bryophyte spores: a palynological study of Erpodiaceae Broth. *Acta Botanica Brasilica* 33: 141-148 (T). ***Moore TE, Schlichting CD, Aiello-Lammens ME, Mocko K, Jones CS. 2018**. Divergent trait and environment relationships among parallel radiations in *Pelargonium* (Geraniaceae): a role for evolutionary legacy? *New Phytologist* 219: 794–807 (T). ***Moreira-Saporiti A, Teichberg M, Garnier E. *et al.* 2023**. A trait-based framework for seagrass ecology: Trends and prospects. *Frontiers in Plant Science* 14: 1088643 (S, T). ***Nazari H, Ghahremaninejad F. 2025**. Micromorphological analysis of leaf trichomes in Australian species of Boraginales. *Turczaninowia* 28, 2: 101–111 (T). ***Pinto-Silva NP, De**

Souza KF, Silva OLM *et al*. 2023. Trichomes in the megadiverse genus *Croton* (Euphorbiaceae): a revised classification, identification parameters and standardized terminology. *Botanical Journal of the Linnean Society* 203: 37–49 (S, T). ***Rodrigues RS, Hartmann LS, Flores AS. 2019**. Seedling morphology of some Brazilian taxa of *Aeschynomene* (Leguminosae) and its systematic relevance. *Flora* 255: 69-79 (S, T). ***Spalink D, Pender J, Escudero M *et al.* 2018**. The spatial structure of phylogenetic and functional diversity in the United States and Canada: An example using the sedge family (Cyperaceae). *Journal of Systematics and Evolution* 56: 449–465 (S). ***Steyn HM, Wyk AE. 2024**. Notes on morphological characteristics and life history strategy of the genus *Acanthopsis* Harv. (Acanthaceae). *Bothalia* 54: 1-15 (T). ***Tank DC, Beardsley PM, Kelchner SA, Olmstead RG. 2006**. Review of the systematics of Scrophulariaceae s.l. and their current disposition. *Australian Systematic Botany* 19: 289–307 (S). ***Wang J-H, Cai Y-F, Li S-F, Zhang S-B. 2020**. Differences in leaf physiological and morphological traits between *Camellia japonica* and *Camellia reticulata*. *Plant Diversity* 42: 181-188 (S). ***Xue B, Guo X, Landis JB. 2020**. Accelerated diversification correlated with functional traits shapes extant diversity of the early divergent angiosperm family Annonaceae. *Molecular Phylogenetics and Evolution* 142: 106659. ***Yao X, Zhang Z-R, Yang J-B, Corlett RT, Li D-Z, Yu W-B. 2025**. An updated plastome phylogeny and molecular dating of Apocynaceae, with an emphasis on histories of biogeography and diversification in tropical and subtropical Asia. *Journal of Systematics and Evolution* 63: 1519–1535 (T).

(S), result in the query (systemat*). (T), result in the query (taxonom*)

Supplementary Table S2.- Populations used in the studies of sexual system, perpetuation mechanism and genetic analyses, indicating plant morphotype and leaf arrangement assignments. We report the values of reproductive functional traits analysed: ligule size and anther length (from an outer and an inner floret of the flower head), and the methodological analysis applied (Methodologies) to the population: ISI, self-incompatibility index; PH, phylogenies based on nr DNA ITS/ETS sequences and three concatenated cpDNA (*ndhJ-trnL, atpI-atpH, and psbJ-petA*) regions; GS, genetic structure by AFLP markers (in parentheses the identification of the populations analysed using AFLP markers). In the analysis of flowers, when possible, three to five specimens were used per population; two specimens per population were used for the morphotypes *S. nymanii* and *S. glaucescens*, due to the scarcity of the morphotypes.

**Locality of populations and morphotype assigned**	**Acronym**	**Geographical Coordinates**	**Elevation** (m a.s.l.)	**Collection Number**	**Ligule size: L × W** (mm)	**Anthers' length: outer** (mm)	**inner**	**Methodologies**
**common *Sonchus asper* (L.) Hill**								
ITALY: Sicily, Alia, road to Montemaggiore	A.AMO[g]	37º46'32"N 013º43'00"E	770	SEV 284735	5.1 × 0.7	1.4	1.5	PH
ITALY: Toscana, San Pellegrino in Alpe	A.PEL[a]	44º10'59"N 010º28'37"E	1520	SEV 284736	4.8 × 0.5	0.8	1.0	ISI, PH, GS
SPAIN: Mallorca, Artá	A.ART[G]	39º44'10.5"N 3º20'05.5"E	220	SEV 284739	3.1 × 0.7	1.2	1.1	PH, GS
SPAIN: Ibiza, pine forest near Cala Jondals	A.IBZ[g]	38º53'15"N 001º19'56"E	100	SEV 284743	3.8 × 1.1	1.6	1.9	PH
SPAIN: Huesca, Puerto de Bonansa	A.PBO[g]	42º25'27"N 000º38'49"W	1390	SEV 284749	4.6 × 0.8	1.0	1.4	PH
SPAIN: Teruel, Tramacastilla	A.TRA[g]	40º25'56"N 001º34'51"W	1330	SEV 284748	3.9 × 0.8	1.0	1.2	PH
SPAIN: Soria, Valonsadero Park	A.VSE[A]	41º48'31"N 002º32'40"W	1040	SEV 284750	4.9 × 1.1	0.8	1.2	PH
SPAIN: Soria, Calatañazor	A.COR[g]	41º42'00"N 002º49'19"W	1025	SEV 284751	4.1 × 0.6	1.3	1.5	PH
SPAIN: Asturias, Cudillero	A.CUD[A]	43º32'29"N 006º07'30"W	240	SEV 284747	4.7 × 0.7	1.0	1.3	PH
SPAIN: Zamora, Quiruelas de Vidriales	A.QUI[g]	42º01'06"N 005º50'57"W	710	SEV 284746	4.5 × 0.8	1.1	1.3	PH
SPAIN: Albacete, Bienservida	A.BIN[G]	38º30'35"N 002º40'19"W	780	SEV 284742	5.0 × 0.8	1.3	1.5	PH
SPAIN: Jaén, Bedmar	A.BED[a]	37º49'37"N 003º26'02"W	525	SEV 284741	4.5 × 0.9	1.0	1.2	PH
SPAIN: Jaén, Marmolejo	A.MJO[A]	38º01'36"N 004º09'48"W	190	SEV 284740	4.9 × 0.8	1.0	1.2	PH
SPAIN: Sevilla, Las Cabezas – Villamartín	A.CAB[A]	36º57'20"N 005º50'48"W	95	SEV 284738	4.5 × 0.8	1.6	1.7	ISI, PH, GS
SPAIN: Cádiz, Sancti Petri, La Barrosa beach	A.BAR[AG]	36º23'01"N 006º11'56"W	sea level	SEV 284744	4.3 × 0.4	0.6	1.1	PH
SPAIN: Huelva, Los Marines – Cortelazor	A.MAR[g]	37º54'41"N 006º37'07"W	665	SEV 284745	5.8 × 1.0	1.0	1.4	ISI, PH,
PORTUGAL: Coimbra, Adémia de Cima	A.COI[A]	40º15'00"N 008º26'34"W	20	SEV 284737	5.0 × 1.1	1.0	1.3	PH
MOROCCO: West Rif, Xauen, road to Ketama	A.XAU[AG]	35º05'52"N 005º15'58"W	570	SEV 284732	5.4 × 0.7	1.0	1.5	PH, GS
MOROCCO: West Rif, Bab Taza – Bab Berret	A.BAD[A]	35º01'06"N 005º00'33"W	1402	SEV 284733	4.7 × 0.9	0.8	1.4	PH, GS
***Sonchus nymanii* Tineo & Guss.**								
ITALY: Sicily, Scillato – Caltavuturo	N.SCI[AG]	37º50'34"N 013º54'14"E	385	SEV 284703	9.5 × 1.9	3.7	4.4	ISI, PH, GS
					11.2 × 2.2	4.4	4.8	
ITALY: Sicily, Valledolmo	N.VAL[G]	37º43'42"N 013º48'01"E	685	SEV 284704	10.4 × 1.7	2.9	3.8	PH, GS
					10.9 × 2.3	3.2	3.7	
ITALY: Sicily, Alia, road to Roccapalumba	N.ALI[AG]	37º46'32"N 013º40'12"E	780	SEV 284705	12.7 × 1.8	3.5	3.8	ISI, PH, GS
					10.3 × 2.1	3.7	4.4	

Locality of populations and morphotype assigned	Acronym	Geographical Coordinates	Elevation (m a.s.l.)	Collection Number	Ligule size: L × W (mm)	Anther length: outer (mm)	inner	Methodologies applied
***Sonchus glaucescens* Jord.**								
FRANCE: Var, île de Gaou	G.GAU[g]	43º10'04"N 005º42'56"E	sea level	SEV 284713	9.5 × 2.0	3.0	4.4	ISI, PH, GS
					10.5 × 2.0	3.4	4.3	
FRANCE: Var, îles d'Hyères, Port - Cross	G.POR[a]	43º00'36"N 006º22'54"E	sea level	SEV 284715	9.2 × 2.1	3.2	4.6	ISI, PH, GS
***Sonchus graecus* E. Weiss**								
TURKEY: Kastamonu, Bulacik Köyü	R.KAS[g]	41º17'50"N 033º46'44"E	1065	SEV 284719	9.5 × 2.0	2.2	3.6	ISI, PH, GS
					10.0 × 2.2	2.2	3.0	
TURKEY: Samsun, Terme – Ḉraşamba	R.TER[g]	41º13'06"N 036º54'26"E	30	SEV 284718	12.3 × 1.9	2.5	3.4	ISI, PH, GS
TURKEY: Izmir, Bornova – Manisa	R.BOR[a])	38º31'56"N 027º16'53"E	440	SEV 284717	9.0 × 1.2	2.9	3.5	PH, GS
TURKEY: Lycia, Finike	R.FIN[g]	36º34'21"N 029º57'19"E	510	SEV 284716	9.7 × 2.1	2.7	3.9	PH, GS
GREECE: Crete, Agios Nikolaus	R.NIK[g]	35º12.'50"N 025º40'11"E	148	SEV 284725	9.5 × 2.0	2.8	3.6	PH, GS
GREECE: Peloponnesus. Eurostini, road to Oros Kyllini	R.EUR[a]	38º06'00"N 022º23'51"E	436	SEV 284720	8.5 × 1.3	2.3	3.3	PH, GS
GREECE: Peloponnesus, Trapeza – Klavryta	R.KAL[AG]	38º07'31"N 022º12'51"E	633	SEV 284721	10.8 × 1.9	2.8	3.6	PH, GS
GREECE: Peloponnesus, Patras – Kyllini, Achaïa beach	R.ACH[g]	38º09'09"N 021º33'42"E	sea level	SEV 284722	9.5 × 1.9	2.4	3.5	ISI, PH, GS
GREECE: Gorinetssia, Mesolorgi – Arta road	R.GOR[g]	38º39'03"N 021º33'42"E	28	SEV 284723	7.7 × 1.3	2.4	3.1	PH
GREECE: Tríkala, Meteora	R.MET[a]	39º43'32"N 021º38'15"E	540	SEV 284724	9.9 × 1.4	2.6	3.5	ISI, PH, GS
GREECE: Grevená	R.GRE[g]	40º05'39"N 021º27'02"E	510	SEV 290032	8.6 × 1.5	2.6	3.6	ISI
					10.4 × 1.6	2.5	3.3	
ITALY: Arruzzi, San Vito Chietino	R.VIT[g]	42º18'30"N 014º26'49"E	sea level	SEV 284729	8.8 × 1.9	2.8	3.3	PH, GS
ITALY: Emilia-Romagna, Cesena – Cervia	R.CES[a]	44º12'56"N 012º20'01"E	3	SEV 284728	9.8 × 1.7	2.5	3.4	ISI, PH, GS
ITALY: Campania, Avellino – Torelli	R.VLL[AG]	40º54'09"N 014º48'17"E	340	SEV 284730	9.3 × 1.7	2.6	3.1	PH, GS
ITALY: Liguria, road to Passo di Bracco	R.BRA[a]	44º14'21"N 009º32'52"E	130	SEV 284727	10.3 × 2.1	2.5	3.6	PH, GS
FRANCE: Var, Hyéres – Le Lavandou, Pas de Courtain	R.LAV[AG]	43º08'59"N 006º17'26"E	75	SEV 284726	10.5 × 1.5	3.3	4.0	ISI, PH, GS

Supplementary Table S3.- Statistical parameters of the bimodal distribution of functional traits of florets associated with the sexual system. M_1 and M_2: modes of the bimodal distribution; T: antimode; A_1 and A_2: amplitude associated with M_1 and M_2, respectively; A_B: bimodal amplitude; A_{an}: antimode amplitude (Zhang *et al.*, 2003); *P*-value: significance obtained in the test for unimodality (Ameijeiras-Alonso *et al.*, 2019; 2021).

**Character**	**M_1 (mm)**	**M_2 (mm)**	**T (mm)**	A_1	A_2	A_B	A_{an}	***P*- value**
Ligule length (outer floret)	4.79	9.50	6.90	0.160	0.141	0.629	0.052	$< 2.2e^{-16}$
Ligule width (outer floret)	0.77	1.72	1.28	0.701	0.583	0.202	0.466	0.034
Anther length (outer floret)	1.15	2.59	1.83	0.466	0.510	0.686	0.147	$< 2.2e\text{-}^{16}$
Anther length (inner floret)	1.54	3.32	2.38	0.419	0.427	0.776	0.094	$< 2.2e\text{-}^{16}$

Supplementary Table S4.- Statistical comparisons for functional traits of florets according to plant habit assignment (subrosulate and no-subrosulate plants : [A] with well-developed leaves exclusively cauline, or [a] mostly cauline; [G] when plants had a well-developed basal rosette and small cauline leaves, or [g] also some well-developed cauline leaves; [AG] was used for intermediate individuals with very doubtful assignment) in the voucher specimes analysed, grouped according outer floret size group. For the plants of the small florets group (N = 118), we used the non-parametric Kruskal-Wallis test (*H* statistic), while for plants of large florets plant group (N = 160) a one-way ANOVA test (*F* statistic) was reliable.

**Habit**	**Small florets group**					**Large florets group**				
		Ligule size		Anther length			Ligule size		Anther length	
	Frequency (%)	length	width	outer	inner	Frequency (%)	length	width	outer	inner
[A]	20.4	4.81 (0.53)	0.78 (0.21)	1.11 (0.26)	1.47 (0.23)	0.0	—	—	—	—
[a]	28.8	4.86 (0.53)	0.75 (0.19)	1.08 (0.27)	1.49 (0.25)	13.8	9.54 (1.31)	1.63 (0.38)	2.60 (0.35)	3.38 (0.47)
[AG]	17.8	4.77 (0.65)	0.77 (0.17)	1.23 (0.22)	1.62 (0.22)	31.9	9.93 (1.78)	1.66 (0.36)	2.75 (0.51)	3.50 (0.54)
[g]	22.0	4.86 (0.85)	0.80 (0.20)	1.21 (0.26)	1.56 (0.27)	30.1	9.80 (1.64)	1.69 (0.37)	2.68 (0.37)	3.45 (0.46)
[G]	11.0	4.45 (0.78)	0.72 (0.12)	1.24 (0.21)	1.44 (0.22)	16.2	9.82 (1.27)	1.65 (0.42)	2.63 (0.34)	3.35 (0.42)
	H value	1.819	1.443	9.423	6.277	*F* value	0.296	0.142	1.217	0.664
	P value	0.769	0.837	0.051	0.179	*P* value	0.828	0.935	0.305	0.575

Supplementary Table S5.- Statistical analysis comparisons for the study of reproductive behaviour. We compared the following pollination treatments in the greenhouse: ASP (Autonomous self-pollination), FSP (hand-forced self-pollination) and MCP (hand multiple individual cross-pollination). In general, we tested overall variability among treatments using the Friedman test for related samples, and paired comparisons between ASP and FSP treatments or FSP and MCP using the Wilcoxon test, given that the data were not normally distributed, either in raw form or after attempting transformations. However, in R.KAS, only ASP vs. MCP (Wilcoxon test) could be performed, and comparisons were not possible with A.PEL. The significance threshold (α) for Friedman tests was 0.05, while for the post-hoc Wilcoxon tests (ASP vs. FCP and FSP vs. MCP) the Bonferroni correction was applied, so α = 0.025.

**Population (Morphotype)**	**N**	**Overall Variability**	**Paired Comparisons**	
			ASP : FSP	FSP : MCP
**common *S. asper* (L.) Hill**				
IT: San Pellegrino in Alpe (A.PEL[a])	6	---	---	---
ES: Villamartín - Las Cabezas (A.CAB[A])[T]	12	χ^2=4.500, *P*=0.105	*Z*=-0.078, *P*=0.937	*Z*=-1.569, *P*=0.117
ES: Los Marines - Cortelazor (A.MAR[g])[T]	8	χ^2=0.065, *P*=0.968	*Z*=0.000, *P*=1.000	*Z*=-0.338, *P*=0.735
***S. nymanii* Tineo & Guss.**				
IT: Sicily, Scillato – Caltavuturo (N.SCI[AG])	8	χ^2=14.769, *P*=0.000	*Z*=-0.447, *P*=0.655	*Z*=-2.521, *P*=0.012
IT: Sicily, Alia (N.ALI[AG])	7	χ^2=10.889, *P*=0.004	*Z*=-0.314, *P*=0.753	*Z*=-2.366, *P*=0.018
***S. glaucescens* Jord.**				
FR: Île de Gaou (G.GAU[g])	10	χ^2=17.657, *P*=0.000	*Z*=-0.674, *P*=0.500	*Z*=-2.803, *P*=0.005
FR: Port-Cross (G.POR)[a]	8	χ^2=14.000, *P*=0.000	*Z*=-1.859, *P*=0.063	*Z*=-2.521, *P*=0.012
***S. graecus* E. Weiss**				
TR: Kastamonu (R.KAS[g])	7	*Z*=-2.366, *P*=0.018	---	---
TR: Terme - Ḉraşamba (R.TER[g])	8	χ^2=14.000, *P*=0.000	*Z*=-1.859, *P*=0.063	*Z*=-2.521, *P*=0.012
GR: Achaïa beach (R.ACH[g])	14	χ^2=22.370, *P*=0.000	*Z*=-1.726, *P*=0.084	*Z*=-3.296, *P*=0.000
GR: Meteora (R.MET[a])	10	χ^2=17.897, *P*=0.000	*Z*=-2.547, *P*=0.011	*Z*=-2.803, *P*=0.005
GR: Grevena (R.GRE[g])	11	χ^2=20.829, *P*=0.000	*Z*=-2.521, *P*=0.012	*Z*=-2.934, *P*=0.003
IT: Cesena – Cervia (R.CES[a])	7	χ^2=10.571, *P*=0.005	*Z*=-0.507, *P*=0.612	*Z*=-2.366, *P*=0.018
FR: Le Pas de Courtin (R.LAV[AG])	10	χ^2=16.270, *P*=0.000	*Z*=-0.338, *P*=0.735	*Z*=-2.805, *P*=0.005

Supplementary Table S6.- Multiple state polymorphisms found in the ITS and ETS aligned sequences of 1,087 concatenated sites of 143 *Sonchus asper* complex samples of 39 populations from the northern Mediterranean.

		ITS (TOTAL 648 SITES)												ETS (TOTAL 439 SITES)													
**Plant Morphotype**	**Accession \ site #**	**41**	**63**	**85**	**150**	**206**	**212**	**239**	**387**	**391**	**402**	**432**	**456**	**733**	**776**	**792**	**806**	**834**	**864**	**904**	**919**	**929**	**932**	**940**	**982**	**989**	**1031**
	A.BED_1	C	G	C	A	T	Y	A	C	Y	C	C	A	G	G	C	T	G	G	A	A	G	A	R	G	A	G
	A.BED_2	C	G	C	A	T	Y	A	C	Y	C	C	A	G	G	C	T	G	G	A	A	G	A	R	G	A	G
	A.BED_3	C	G	C	A	T	Y	A	C	Y	C	C	A	G	G	C	T	G	G	A	A	G	A	R	G	A	G
	A.BIN_1	T	G	C	A	T	Y	A	C	C	C	C	A	G	G	C	T	G	G	A	A	G	A	G	G	A	G
	A.BIN_2	Y	G	C	A	T	Y	A	C	C	C	C	A	G	G	C	T	G	G	A	A	G	A	G	G	A	G
	A.BIN_3	Y	G	C	A	T	Y	A	C	C	C	C	A	G	G	C	T	G	G	A	A	G	A	G	G	A	G
	A.BAR_2	C	G	C	A	T	Y	A	C	Y	C	C	A	G	G	C	T	G	G	A	A	G	A	R	G	A	G
	A.BAR_3	T	G	C	A	T	Y	A	C	Y	C	C	A	G	G	C	T	G	G	A	A	G	A	G	G	A	G
	A.BAR_4	Y	G	C	A	T	Y	A	C	C	C	C	A	G	G	C	T	G	G	A	A	G	A	R	G	A	G
	A.COR_1	Y	G	C	A	T	Y	A	C	C	C	C	A	G	G	C	T	G	G	A	A	G	A	G	G	A	G
	A.COR_2	T	G	C	A	T	C	A	C	C	C	C	A	G	G	C	T	G	G	A	A	G	A	G	G	A	G
	A.COR_3	T	G	C	A	T	C	A	C	C	C	C	A	G	G	C	T	G	G	A	A	G	A	G	G	A	G
	A.VSE_1	C	G	C	A	T	Y	A	C	C	C	C	A	G	G	C	T	G	G	A	A	G	A	G	G	A	G
	A.VSE_2	C	G	C	A	T	Y	A	C	C	C	C	A	G	G	C	T	G	G	A	A	G	A	G	G	A	G
	A.TRA_1	T	G	C	A	T	C	A	C	C	C	C	A	G	R	C	T	G	G	A	A	G	A	G	G	A	G
	A.TRA_2	T	G	C	A	T	C	A	C	C	C	C	A	G	R	C	T	G	G	A	A	G	A	G	G	A	G
	A.TRA_3	T	G	C	A	T	C	A	C	C	C	C	A	G	G	C	T	G	G	A	A	G	A	G	G	A	G
S. asper	A.PBO_1	Y	G	C	A	T	Y	A	C	C	C	C	A	G	G	C	T	G	G	A	A	G	A	G	G	A	G
	A.PBO_2	Y	G	C	A	T	Y	A	C	C	C	C	A	G	G	C	T	G	G	A	A	G	A	G	G	A	G
	A.PBO_3	Y	G	C	A	T	Y	A	C	C	C	C	A	G	G	C	T	G	G	A	A	G	A	G	G	A	G
	A.AMO_1	T	G	C	A	T	C	A	C	C	C	C	A	G	G	C	T	G	G	A	A	G	A	G	G	A	G
	A.AMO_2	T	G	C	A	T	C	A	C	C	C	C	A	G	G	C	T	G	G	A	A	G	A	G	G	A	G
	A.AMO_3	T	G	C	A	T	C	A	C	C	C	C	A	G	G	C	T	G	G	A	A	G	A	G	G	A	G
	A.AMO_4	T	G	C	A	T	C	A	C	C	C	C	A	G	G	C	T	G	G	A	A	G	A	G	G	A	G
	A.PEL_1	Y	G	C	A	T	Y	A	C	C	C	C	A	G	G	C	T	G	G	A	A	G	A	G	G	A	G
	A.PEL_2	Y	G	C	A	T	Y	A	C	C	C	C	A	G	G	C	T	G	G	A	A	G	A	G	G	A	G
	A.PEL_3	Y	G	C	A	T	Y	A	C	C	C	C	A	G	G	C	T	G	G	A	A	G	A	G	G	A	G
	A.PEL_4	Y	G	C	A	T	Y	A	C	C	C	C	A	G	G	C	T	G	G	A	A	G	A	G	G	A	G
	A.PEL_5	Y	G	C	A	T	Y	A	C	C	C	C	A	G	G	C	T	G	G	A	A	G	A	G	G	A	G
	A.XAU_2	T	G	C	A	T	C	A	C	C	C	C	T	G	G	C	T	G	G	A	A	G	A	G	G	A	G
	A.XAU_4	T	G	C	A	T	C	A	C	C	C	C	T	G	G	C	T	G	G	A	A	G	A	G	G	A	G
	A.BAD_1	T	G	C	A	T	C	A	C	C	C	C	T	G	G	C	T	G	G	A	A	G	A	G	G	A	G
	A.BAD_2	T	G	C	A	T	C	A	C	C	C	C	T	G	G	C	T	G	G	A	A	G	A	G	G	A	G
	A.BAD_3	T	G	C	A	T	C	A	C	C	C	C	T	G	G	C	T	G	G	A	A	G	A	G	G	A	G
	A.BAD_4	T	G	C	A	T	C	A	C	C	C	C	T	G	G	C	T	G	G	A	A	G	A	G	G	A	G

	A.CAB_1	Y	G	C	A	T	Y	A	C	C	C	C	A	G	G	C	T	G	G	A	A	G	A	R	G	A	G
	A.CAB_2	C	G	C	A	T	Y	A	C	C	C	C	A	G	G	C	T	G	G	A	A	G	A	R	G	A	G
	A.CAB_3	C	G	C	A	T	Y	A	C	C	C	C	A	G	G	C	T	G	G	A	A	G	A	R	G	A	G
	A.CAB_5	C	G	C	A	T	Y	A	C	C	C	C	A	G	G	C	T	G	G	A	A	G	A	R	G	A	G
	A.ART_1	C	G	C	A	T	Y	A	C	C	C	Y	A	G	G	C	T	G	G	A	A	G	A	G	G	A	G
	A.ART_2	T	G	C	A	T	C	A	C	C	C	C	A	G	G	C	T	G	G	A	A	G	A	G	G	A	G
	A.ART_3	Y	G	C	A	T	Y	A	C	C	C	Y	A	G	R	C	T	G	G	A	A	G	A	G	G	A	G
	A.ART_4	T	G	C	A	T	Y	A	C	C	C	C	A	G	R	C	Y	G	G	A	A	G	A	G	G	A	G
	A.ART_5	Y	G	C	A	T	Y	A	C	C	C	C	A	G	R	C	Y	G	G	A	A	G	A	G	G	A	G
	A.IBZ_1	Y	G	C	A	T	Y	A	C	C	C	C	A	G	R	C	Y	G	G	A	A	G	A	G	G	A	G
	A.IBZ_2	T	G	Y	A	T	C	A	C	C	C	C	A	G	G	C	T	G	G	A	A	G	A	G	G	A	G
	A.IBZ_3	Y	G	C	A	T	Y	A	C	C	C	C	A	G	R	C	Y	G	G	A	A	G	A	G	G	A	G
	A.CUD_1	T	G	C	A	T	C	A	C	C	C	C	W	G	G	C	T	G	G	A	A	G	A	G	G	A	G
	A.CUD_2	T	G	C	A	T	C	A	C	C	C	C	A	G	G	C	T	G	G	A	A	G	A	G	G	A	G
	A.CUD_3	T	G	C	A	T	C	A	C	C	C	C	A	G	G	C	T	G	G	A	A	G	A	G	G	A	G
	A.COI_1	Y	G	C	A	T	Y	A	C	C	C	C	A	G	G	C	T	G	G	A	A	G	A	G	G	A	G
	A.COI_2	Y	G	C	A	T	Y	A	C	C	C	C	A	G	G	C	T	G	G	A	A	G	A	G	G	A	G
	A.COI_3	Y	G	C	A	T	Y	A	C	C	C	C	A	G	G	C	T	G	G	A	A	G	A	G	G	A	G
	A.QUI_1	Y	G	C	A	T	Y	A	C	C	C	C	A	G	G	C	T	G	G	A	W	G	A	G	G	A	G
	A.QUI_2	Y	G	C	A	T	Y	A	C	C	C	C	A	G	G	C	T	G	G	A	W	G	A	G	G	A	G
	A.QUI_3	Y	G	C	A	T	Y	A	C	C	C	C	A	G	G	C	T	G	G	A	A	G	A	G	G	A	G
	A.MAR_1	T	G	C	A	T	C	A	C	C	C	C	T	G	G	C	T	G	G	A	A	G	A	G	G	A	R
	A.MAR_2	T	G	C	A	T	C	A	C	C	C	C	A	G	G	S	T	G	G	A	A	G	A	G	G	A	G
	A.MAR_5	T	G	C	A	T	C	A	C	C	C	C	A	G	G	C	T	G	G	A	A	G	A	G	G	A	G
	A.MJO_1	Y	G	C	A	T	Y	A	C	C	C	C	A	G	G	C	T	G	G	A	A	G	A	G	G	A	G
	A.MJO_2	Y	G	C	A	T	Y	A	C	C	C	C	W	G	G	C	T	G	G	A	A	G	A	G	G	A	G
	A.MJO_3	C	G	C	A	T	Y	A	C	Y	C	C	A	G	G	C	T	G	G	A	A	G	A	R	G	A	G
S. graecus	R.LAV_1	T	G	C	A	T	C	A	C	C	C	C	A	G	G	C	T	A	G	A	A	G	T	G	G	A	G
	R.LAV_3	T	G	C	M	T	C	R	C	C	C	C	A	G	G	C	T	R	G	A	A	G	W	R	G	A	G
	R.LAV_4	T	G	C	A	T	C	A	C	C	C	C	A	G	G	C	T	R	G	A	A	G	T	G	G	A	G
	R.LAV_5	T	G	C	M	T	C	A	C	C	C	C	A	?	?	?	?	?	?	?	?	?	?	?	?	?	?
	R.ACH_1	T	G	C	A	T	C	A	C	C	C	C	A	G	G	C	T	A	G	A	A	G	T	G	G	A	G
	R.ACH_2	T	G	C	A	T	C	A	C	C	C	C	A	G	G	C	T	R	G	A	A	G	W	G	G	A	G
	R.ACH_3	T	G	C	A	T	C	A	C	C	C	C	A	G	G	C	T	R	G	A	A	G	W	G	G	A	G
	R.ACH_4	T	G	C	A	T	C	A	C	C	C	C	A	G	G	C	T	A	G	A	A	G	T	G	G	A	G
	R.ACH_5	T	G	C	A	T	C	A	C	C	C	C	A	G	G	C	T	A	G	A	A	G	T	G	G	A	G
	R.MET_1	T	G	C	A	T	C	A	C	C	C	C	A	G	G	C	T	A	G	A	A	G	T	G	G	A	G
	R.MET_2	T	G	C	A	T	C	A	Y	C	C	C	A	G	G	C	T	A	G	A	A	G	T	G	G	A	G
	R.MET_3	T	G	C	A	T	C	A	C	C	C	C	A	G	G	C	T	A	G	A	A	G	T	G	G	A	G
	R.MET_4	T	G	C	A	T	C	A	C	C	C	C	A	G	G	C	T	A	G	A	A	G	T	G	G	A	G
	R.MET_5	T	G	C	A	T	C	A	C	C	C	C	A	G	G	C	T	R	G	A	A	G	T	G	G	A	G
	R.CES_1	T	G	C	M	T	C	R	C	C	C	C	A	G	G	C	T	R	G	A	A	G	W	R	G	W	G
	R.CES_2	T	G	C	M	T	C	G	C	C	C	C	A	G	G	C	T	R	G	A	A	G	W	R	G	W	G
	R.CES_3	T	G	C	A	T	C	A	C	C	C	C	A	G	G	C	T	R	G	A	A	G	W	R	G	A	G

	R.CES_4	T	G	C	**M**	T	C	**R**	C	C	C	C	A	G	G	C	T	**R**	G	A	A	G	**W**	**R**	G	**W**	G
	R.CES_5	T	G	C	**M**	T	C	G	C	C	C	C	A	G	G	C	T	**R**	G	A	A	G	**W**	**R**	G	**W**	G
	R.VLL_1	T	G	C	**M**	T	C	**R**	C	C	C	C	A	G	G	C	T	**R**	G	A	A	G	**W**	**R**	G	**W**	G
	R.VLL_2	T	G	C	A	T	C	A	C	C	C	C	A	G	G	C	T	A	G	A	A	G	T	G	G	A	G
	R.VLL_3	T	G	C	**M**	T	C	**R**	C	C	C	C	A	G	G	C	T	**R**	G	A	A	G	**W**	**R**	G	**W**	G
	R.VLL_4	T	G	C	**M**	T	C	**R**	C	C	C	C	A	G	G	C	T	**R**	G	A	A	G	**W**	**R**	G	**W**	G
	R.VLL_5	T	G	C	A	T	C	A	C	C	C	C	A	G	G	C	T	A	G	A	A	G	T	G	G	A	G
	R.FIN_1	T	G	C	A	T	C	A	**Y**	C	C	C	A	G	G	C	T	A	G	A	A	G	T	G	G	A	G
	R.FIN_2	T	G	C	A	T	C	A	C	C	C	C	A	G	G	C	T	A	G	A	A	G	T	G	G	A	G
	R.FIN_3	T	G	C	A	T	C	A	C	C	C	C	A	G	G	C	T	A	G	A	A	G	T	G	G	A	G
	R.FIN_4	T	G	C	A	T	C	A	**Y**	C	C	C	A	G	G	C	T	A	G	A	A	G	T	G	G	A	G
	R.FIN_5	T	G	C	A	T	C	A	C	C	C	C	A	G	G	C	T	A	G	A	A	G	T	G	G	A	G
	R.KAS_1	T	G	C	A	T	C	A	**Y**	C	C	C	A	G	G	C	T	A	G	A	A	G	T	G	G	A	G
	R.KAS_2	T	G	C	A	T	C	A	**Y**	C	C	C	A	G	G	C	T	A	G	A	A	G	T	G	G	A	G
	R.KAS_3	T	G	C	A	T	C	A	C	C	C	C	A	G	G	C	T	A	G	A	A	G	T	G	G	A	G
	R.KAS_4	T	G	C	A	T	C	A	C	C	C	C	A	G	G	C	T	A	G	A	A	G	T	G	G	A	G
	R.KAS_5	T	G	C	A	T	C	A	C	C	C	C	A	G	G	C	T	A	G	A	A	G	T	G	G	A	G
	R.NIK_1	T	G	C	A	T	C	A	C	C	C	C	A	G	G	C	T	**R**	G	A	A	G	**W**	**R**	**R**	A	G
	R.NIK_2	T	G	C	A	T	C	A	C	C	C	C	A	G	G	C	T	A	G	A	A	G	T	G	G	A	G
	R.NIK_3	T	G	C	A	T	C	A	C	C	C	C	A	G	G	C	T	A	G	A	A	G	T	**R**	**R**	A	G
	R.EUR_1	T	G	C	A	T	C	A	C	C	C	C	A	G	G	C	T	A	G	A	A	G	T	G	G	A	G
	R.EUR_4	T	G	C	A	T	C	A	C	C	C	C	A	G	G	C	T	A	G	A	A	G	T	G	G	A	G
	R.EUR_5	T	G	C	A	T	C	A	C	C	C	C	A	G	G	C	T	A	G	A	A	G	T	G	G	A	G
	R.KAL_1	T	G	C	A	T	C	A	C	C	C	C	A	G	G	C	T	A	G	A	A	G	T	**R**	G	A	G
	R.KAL_2	T	G	C	A	T	C	A	C	C	C	C	A	G	G	C	T	A	G	A	A	G	T	G	G	A	G
	R.KAL_3	T	G	C	A	T	C	A	C	C	C	C	A	G	G	C	T	A	G	A	A	G	T	G	G	A	G
	R.GOR_1	T	G	C	A	T	C	A	C	C	C	C	A	G	G	C	T	A	G	A	A	G	T	G	G	A	G
	R.GOR_2	T	G	C	A	T	C	A	C	C	C	C	A	G	G	C	T	A	G	A	A	G	T	G	G	**W**	G
	R.BRA_1	T	G	C	**M**	T	C	**R**	C	C	C	C	A	G	G	C	T	**R**	G	A	A	G	**W**	**R**	G	**W**	G
	R.BRA_2	T	G	C	A	T	C	A	C	C	C	C	A	G	G	C	T	A	G	A	A	G	T	G	G	A	G
	R.BRA_3	T	G	C	A	T	C	A	**Y**	C	C	C	A	G	G	C	T	**R**	G	A	A	G	**W**	**R**	**R**	A	G
	R.VIT_1	T	G	C	**M**	T	C	A	C	C	C	C	A	G	G	C	T	**R**	G	A	A	G	**W**	**R**	G	**W**	G
	R.VIT_2	T	G	C	**M**	T	C	**R**	C	C	C	C	A	G	G	C	T	A	G	A	A	G	**W**	**R**	G	**W**	G
	R.VIT_3	T	G	C	A	T	C	A	C	C	C	C	A	G	G	C	T	A	G	A	A	G	T	G	G	A	G
	R.BOR_1	T	G	C	A	T	C	A	**Y**	C	C	C	A	G	G	C	T	A	G	A	A	G	T	G	G	A	G
	R.BOR_2	T	G	C	A	T	C	A	**Y**	C	C	C	A	G	G	C	T	A	G	A	A	G	T	G	G	A	G
	R.BOR_3	T	G	C	A	T	C	A	**Y**	C	C	C	A	G	G	C	T	A	G	A	A	G	T	G	G	A	G
	R.TER_2	T	G	C	A	T	C	A	**Y**	C	C	C	A	G	G	C	T	A	G	A	A	G	T	G	G	A	G
	R.TER_3	T	G	C	A	T	C	A	**Y**	C	C	C	A	G	G	C	T	A	G	A	A	G	T	G	G	A	G
	R.TER_4	T	G	C	A	T	C	A	**Y**	C	C	C	A	G	G	C	T	A	G	A	A	G	T	G	G	A	G
S. nymanii	N.SCI_1	T	G	C	A	T	C	A	C	C	C	C	A	G	G	C	T	G	**S**	A	A	G	A	G	G	A	G
	N.SCI_2	T	**K**	C	A	T	C	A	C	C	C	C	A	G	G	C	T	G	G	A	A	G	A	G	G	A	G
	N.SCI_3	T	**K**	C	A	T	C	A	C	C	C	C	A	G	G	C	T	G	G	A	A	G	A	G	G	A	G
	N.SCI_4	T	**K**	C	A	T	C	A	C	C	C	C	A	G	G	C	T	G	G	A	A	G	A	G	G	A	G

	N.SCI_5	T	**K**	C	A	T	C	A	C	C	C	C	A	**R**	G	C	T	G	**S**	A	A	G	A	G	G	A	G
	N.VAL_1	T	**K**	C	A	T	C	A	C	C	C	C	A	G	G	C	T	G	G	A	A	G	A	G	G	A	G
	N.VAL_2	T	**K**	C	A	**Y**	C	A	C	C	C	C	A	**R**	G	C	T	G	**S**	**R**	A	**K**	A	G	G	A	G
	N.VAL_3	T	**K**	C	A	T	C	A	C	C	C	C	A	G	G	C	T	G	**S**	A	A	G	A	G	G	A	G
	N.VAL_4	T	**K**	C	A	T	C	A	C	C	C	C	A	G	G	C	T	G	**S**	A	A	G	A	G	G	A	G
	N.VAL_5	T	**K**	C	A	T	C	A	C	C	C	C	A	**R**	G	C	T	G	**S**	A	A	G	A	G	G	A	G
	N.ALI_1	T	**K**	C	A	**Y**	C	A	C	C	**Y**	C	A	**R**	G	C	T	G	**S**	A	A	G	A	G	G	A	G
	N.ALI_2	T	G	C	A	C	C	A	C	C	T	C	A	A	G	C	T	G	C	A	A	T	A	G	G	A	G
	N.ALI_3	T	G	C	A	C	C	A	C	C	**Y**	C	A	A	G	C	T	G	C	A	A	T	A	G	G	A	G
	N.ALI_4	T	**K**	C	A	T	C	A	C	C	C	C	A	G	G	C	T	G	**S**	A	A	G	A	G	G	A	G
	N.ALI_5	T	**K**	C	A	T	C	A	C	C	C	C	A	G	G	C	T	G	**S**	A	A	G	A	G	G	A	G
	G.GAU_1	T	G	C	**M**	T	C	**R**	C	C	C	C	A	G	G	C	T	**R**	G	A	A	G	**W**	**R**	G	**W**	**R**
	G.GAU_2	T	G	C	**M**	T	C	**R**	C	C	C	C	A	G	G	C	T	**R**	G	A	A	G	**W**	**R**	G	**W**	**R**
	G.GAU_3	T	G	C	**M**	T	C	**R**	C	C	C	C	A	G	G	C	T	**R**	G	A	A	G	**W**	**R**	G	**W**	**R**
	G.GAU_4	T	G	C	**M**	T	C	**R**	C	C	C	C	A	G	G	C	T	**R**	G	A	A	G	**W**	**R**	G	**W**	**R**
S. glaucescens	G.GAU_5	T	G	C	C	T	C	G	C	C	C	C	A	G	G	C	T	**R**	G	A	A	G	**W**	**R**	G	**W**	**R**
	G.POR_1	T	G	C	A	T	C	A	C	C	C	C	A	G	G	C	T	A	G	A	A	G	T	G	G	A	G
	G.POR_2	T	G	C	A	T	C	A	C	C	C	C	A	G	G	C	T	**R**	G	A	A	G	**W**	**R**	G	A	G
	G.POR_3	T	G	C	A	T	C	A	C	C	C	C	A	G	G	C	T	**R**	G	A	A	G	**W**	**R**	G	A	G
	G.POR_4	T	G	C	A	T	C	A	C	C	C	C	A	G	G	C	T	**R**	G	A	A	G	**W**	**R**	G	A	G
	G.POR_5	T	G	C	A	T	C	A	C	C	C	C	A	G	G	C	T	A	G	A	A	G	T	G	G	A	G

IUPAC Codes for Ambiguous Bases: R (A or G), Y (C, T), M (A, C), K (G, T), S (G, C), W (A, T)

Supplementary Table S7.- Haplotypes found based on the sequences of three concatenated cpDNA (*ndhJ-trnL*, *atpI-atpH*, and *psbJ-petA*) regions, indicating the accessions where they were detected.

**Haplotypes**	**Q'ty**	**Morphotypes**	**Accessions**
H1	52	*S. asper* (26)	A.BAR_2, A.BAR_3, A.COR_3, A.VSE_1, A.VSE_2, A.PBO_1, A.PBO_2, A.PBO_3, A.AMO_2, A.AMO_3, A.PEL_3, A.PEL_4, A.XAU_2, A.XAU_4, A.ART_1, A.IBZ_2, A.CUD_2, A.COI_1, A.COI_2, A.COI_3, A.QUI_1, A.QUI_2, A.QUI_3, A.MAR_1, A.MAR_2, A.MAR_5
		S. graecus (17)	R.LAV_1, R.CES_4, R.VLL_1, R.VLL_4, R.FIN_1, R.FIN_2, R.FIN_3, R.KAS_2, R.KAS_4, R.KAS_5, R.BRA_1, R.BRA_3, R.VIT_2, R.VIT_3, R.TER_2, R.TER_3, R.TER_4
		S. glaucescens (9)	G.GAU_1, G.GAU_2, G.GAU_4, G.GAU_5, G.POR_1, G.POR_2, G.POR_3, G.POR_4, G.POR_5
H2	16	*S. asper* (15)	A.BED_1, A.BED_2, A.BED_3, A.BIN_1, A.BIN_2, A.BIN_3, A.BAR_4, A.TRA_1, A.TRA_2, A.BAD_1, A.BAD_2, A.BAD_3, A.CAB_1, A.ART_2, A.MJO_1
		S. graecus (1)	R.VIT_1
H3	5	*S. asper* (5)	A.CAB_2, A.CAB_3, A.CAB_5, A.ART_4, A.MJO_3
H4	15	*S. asper* (8)	A.COR_2, A.PEL_1, A.PEL_2, A.PEL_5, A.ART_3, A.ART_5, A.IBZ_1, A.IBZ_3
		S. graecus (7)	R.LAV_3, R.LAV_4, R.LAV_5, R.CES_1, R.CES_2, R.CES_3, R.BRA_2
H5	1	*S. glaucescens* (1)	G.GAU_3
H6	6	*S. asper* (2)	A.BAD_4, A.MJO_2
		S. graecus (4)	R.NIK_3, R.EUR_1, R.EUR_5, R.KAL_1
H7	4	*S. graecus* (4)	R.ACH_1, R.ACH_2, R.ACH_3, R.ACH_4
H8	4	*S. asper* (4)	A.COR_1, A.TRA_3, A.CUD_1, A.CUD_3
H9	1	*S. graecus* (1)	R.GOR_2
H10	1	*S. graecus* (1)	R.EUR_4

Haplotypes	Q'ty	Morphotypes	Accessions
H11	5	*S. graecus* (2)	R.KAS_3, R.KAS_1
		S. nymanii (3)	N.SCI_1, N.SCI_2, N.ALI_2
H12	1	*S. nymanii* (1)	N.ALI_4
H13	23	*S. asper* (2)	A.AMO_1, A.AMO_4
		S. graecus (15)	R.MET_1, R.MET_4, R.MET_5, R.CES_5, R.VLL_2, R.VLL_3, R.VLL_5, R.FIN_4, R.NIK_1, R.NIK_2, R.KAL_2, R.KAL_3, R.BOR_1, R.BOR_2, R.BOR_3
		*S. nymanii (*6)	N.SCI_3, N.SCI_4, N.VAL_2, N.ALI_1, N.ALI_3, N.ALI_5
H14	1	*S. nymanii* (1)	N.VAL_1
H15	1	*S. graecus* (1)	R.FIN_5
H16	1	*S. nymanii* (1)	N.SCI_5
H17	1	*S. nymanii* (1)	N.VAL_5
H18	1	*S. nymanii* (1)	N.VAL_3
H19	3	*S. graecus (*3)	R.MET_2, R.MET_3, R.ACH_5
H20	1	*S. nymanii (*1)	N.VAL_4

Supplementary Table S8.- Haplotype distribution pattern in the samples, based on the sequences of three concatenated cpDNA (*ndhJ-trnL*, *atpI-atpH*, and *psbJ-petA*) regions. Red is used for haplotypes that were unique to one morphotype.

**Locality of populations and morphotype**	**Acronym**	**Haplotypes**																			
		**H1**	**H2**	**H3**	**H4**	**H5**	**H6**	**H7**	**H8**	**H9**	**H10**	**H11**	**H12**	**H13**	**H14**	**H15**	**H16**	**H17**	**H18**	**H19**	**H20**
**common *Sonchus asper* (L.) Hill**																					
ITALY: Sicily, Alia, road to Montemaggiore	A.AMO[g]	2												2							
ITALY: Toscana, San Pellegrino in Alpe	A.PEL[a]	2			3																
SPAIN: Isla de Mallorca, Artá	A.ART[G]	1	1	**1**	2																
SPAIN: Isla de Ibiza, near Cala Jondals	A.IBZ[g]	1			2																
SPAIN: Huesca, Puerto de Bonansa	A.PBO[g]	3																			
SPAIN: Teruel, Tramacastilla	A.TRA[g]		2						**1**												
SPAIN: Soria, Valonsadero Park	A.VSE[A]	2																			
SPAIN: Soria, Calatañazor	A.COR[g]	1			1				**1**												
SPAIN: Asturias, Cudillero	A.CUD[A]	1							**2**												
SPAIN: Zamora, Quiruelas de Vidriales	A.QUI[g]	3																			
SPAIN: Albacete, Bienservida	A.BIN[G]		3																		
SPAIN: Jaén, Bedmar	A.BED[a]		3																		
SPAIN: Jaén, Marmolejo	A.MJO[A]																				
SPAIN: Sevilla, Villamartín – Las Cabezas	A.CAB[A]		1	**3**																	
SPAIN: Cádiz, Sancti Petri, La Barrosa beach	A.BAR[AG]	2	1																		
SPAIN: Huelva, Los Marines – Cortelazor	A.MAR[g]	3																			
PORTUGAL: Coimbra, Adémia de Cima	A.COI[A]	3																			
MOROCCO: West Rif, Xauen, road to Ketama	A.XAU[AG]	2																			
MOROCCO: West Rif, Bab Taza – Bab Berret	A.BAD[A]		3																		
***Sonchus nymanii* Tineo & Guss.**																					
ITALY: Sicily, Scillato – Caltavuturo	N.SCI[AG]											2		2			**1**		**1**		
ITALY: Sicily, Valledolmo	N.VAL[G]													1	**1**			**1**			**1**
ITALY: Sicily, Alia, road to Roccapalumba	N.ALI[AG]											1	**1**	3							
***Sonchus glaucescens* Jord.**																					
FRANCE: Var, île de Gaou	G.GAU[g]	4				**1**															
FRANCE: Var, îles d'Hyères, Port - Cross	G.POR[g]	5																			
***Sonchus graecus* E. Weiss**																					
TURKEY: Kastamonu, Bulacik Köyü	R.KAS[g]	3										2									
TURKEY: Samsun, Terme – Ḉraşamba	R.TER[g]	3																			
TURKEY: Izmir, Bornova – Manisa	R.BOR[a])													3							
TURKEY: Lycia, Finike	R.FIN[g]	3										1				**1**					
GREECE: Crete, Agios Nikolaus	R.NIK[g]						1							2							
GREECE: Peloponnesus. Eurostini.	R.EUR[a]						2				**1**										
GREECE: Peloponnesus, Trapeza – Klavryta	R.KAL[AG]						1							2							
GREECE: Peloponnesus, Patras – Kyllini, Achaïa	R.ACH[g]							**4**												**1**	
GREECE: Gorinetssia, Mesolorgi – Arta road	R.GOR[g]									**1**											
GREECE: Tríkala, Meteora	R.MET[a]													3						**2**	
ITALY: Arruzzi, San Vito Chietino	R.GRE[g]	2	1																		
ITALY: Emilia-Romagna, Cesena – Cervia	R.VIT[g]	1			3																
ITALY: Campania, Avellino – Torelli	R.CES[a]	2												3							
ITALY: Liguria, road to Passo di Bracco	R.VLL[AG]	2			1																
FRANCE: Var, Hyéres – Le Lavandou.	R.BRA[a]	1			3																

Supplementary Figure S1. Geographical locations of the populations studied, indicating the morphotype and habit assignments. Morphotypes: *S. asper* (L.) Hill (; acronym: A.); *S. nymanii* Tineo & Guss. (; acronym: N.); *S. glaucescens* Jord. (; acronym: G); *S. graecus* E. Weiss (; acronym: R). Plant habit: [A] plants with well-developed leaves exclusively cauline, or [a] mostly cauline (subsp. *asper*); [G] plants with a well-developed basal rosette and small cauline leaves, or [g] also with some well-developed cauline leaves (subsp. *glaucescens*); [AG] was used for intermediate individuals that could not be reliably assigned.

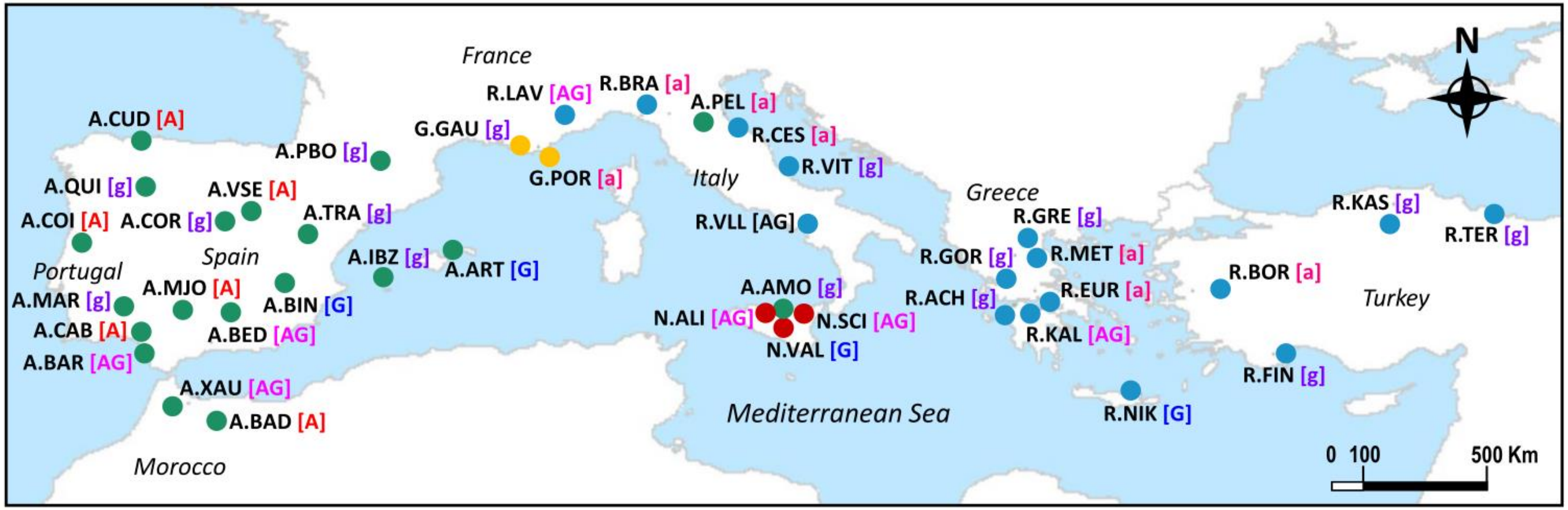

Supplementary Figure S2.- ML phylogenetic tree based on the sequences of three concatenated cpDNA regions, *ndhJ-trnF*(GAA), *atpI-atpH*, and *psbJ-petA*. Bootstrap values are shown on the major branches and the morphotype assigned to each sample is indicated by colours, according to indications in the figure. Populations are indicated by acronyms according to Supplementary Data Table S2 and Fig. S1.

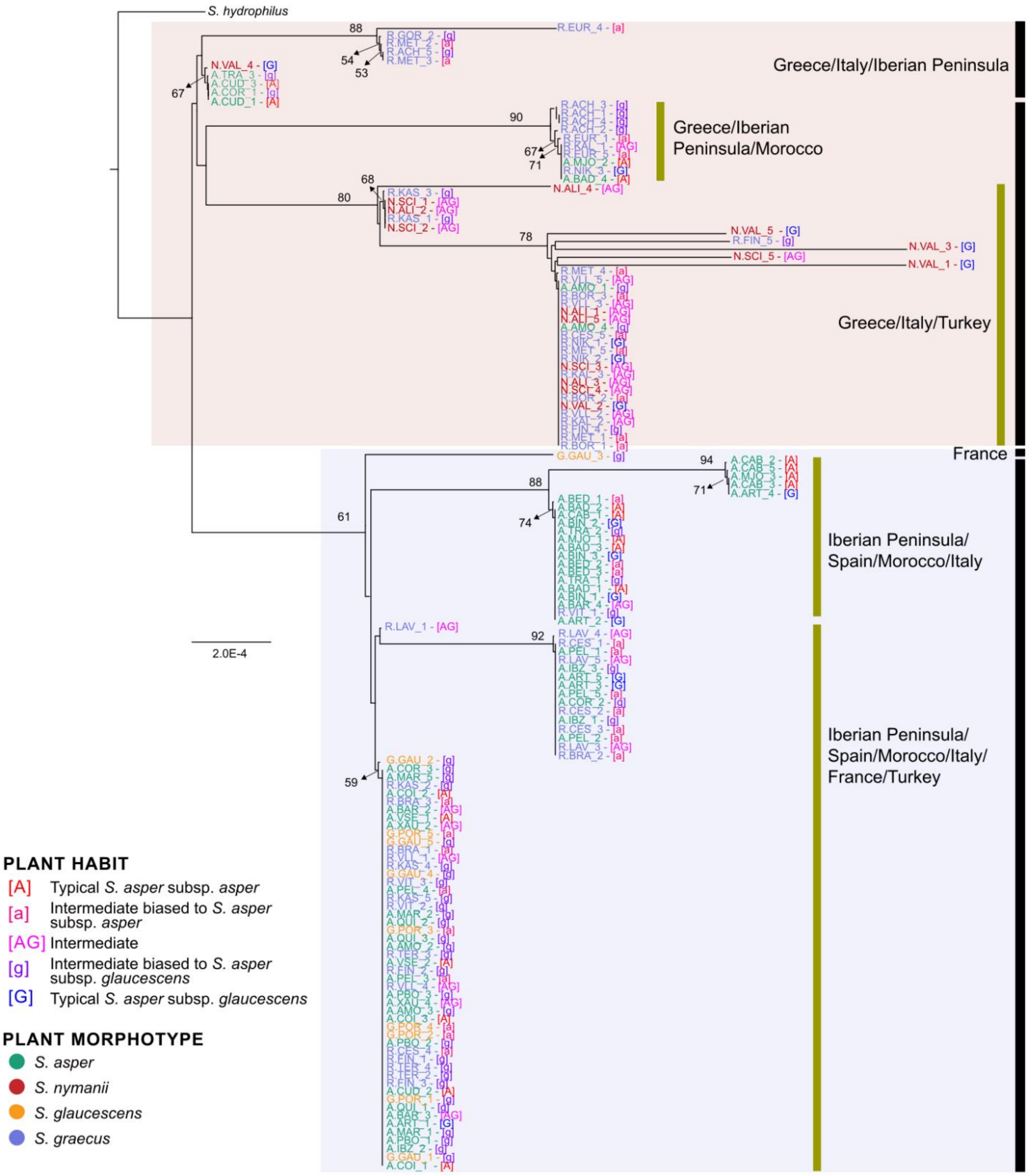

Supplementary Figure S3. Delta-K estimates using data from AFLP markers in 240 individuals of *Sonchus asper* complex from northern Mediterranean Basin, according to methods of Evanno *et al.* (2005) in Structure Harvester.

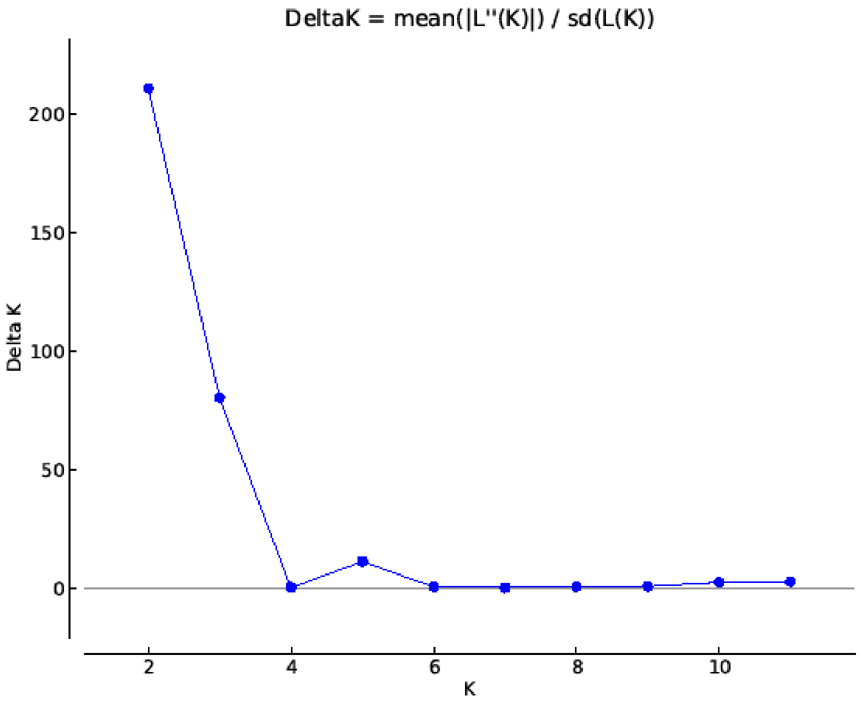

Supplementary Appendix S1.- Herbarium specimens (*exsiccata*) examined and measured to analyse morphotype distribution within the *Sonchus asper* complex across the study area, ordered by herbarium where they are registered. Taxonomic identification was performed in accordance with the criteria of taxonomic treatment.

B: ZE BOTANISCHER GARTEN UND BOTANISCHES MUSEUM, FREIE UNIVERSITÄT BERLIN. BERLIN, GERMANY

1. *Sonchus asper* (L.) Hill

GREECE: Kriti: Nomos Lasithiou: Eparhia Mirabellou Oropedio (mountain plain) 500 m, SW of Kastelli E of Neapoli, 35º15'23.5"N 25º38'12.7"E, 290 m, 05.04.2003, surrounded by similar fields, vineyards and olive groves, Bareka & Turland (B 10 0192714)
GREECE: Kriti: Nomos Rethimnis: Eparhia Agiou Vasiliou Small oropedio (mountain plain) n of Sellis, between village and mountain ridge, 405 m, 35º12'38.4"N 24º22'52.5"E, Kamari & Turland (B 10 0193398)
GREECE: Kriti: Eparchie Rethimni: Petres-Schlucht N von Karoti, Unkrautfluren, Feuchtstellen und Ufer im Talgrund am Schluchtausgang, sandige Alluvialböden, 5-10 m, 13.06.1982, 35º20'30"N 24º21'30"E, H. Risse (B 10 0591039)
GREECE: Kriti, Kommas, Pitsidia, between Kommos and beach, irregular stepped slope of exposed outcrop, rock tumble, with erosion gullies, 10.05.1982, J.M. Shay (B 10 0591053)
GREECE: Thesprotia, SW Paleochori, 08.05.2005, Hartlaubgebüsch, Krautfluren, Stranßenrand, 100 m, 39º32'08"N 20º26'02"E, R. & E. Willing (B 10 0211744)
GREECE: Grevena, Ep. Grevenon, SW Vatolakko. Aufgelassener Acker, Straßenrand. 500m. 40º08'43"N 21º28'17"E. 23.07.2000. R. Eisenblaätter & E. Willing (B 10 0591050)
GREECE: Kriti, Kommas, Pitsidia, between Kommos and beach, irregular stepped slope of exposed outcrop, rock tumble, with erosion gullies, 10.05.1982, J.M. Shay (B 10 0591053)
GREECE: Florina, Ep. Florinis, OSO Karye. Sandige Hänge, Eichenbuschwald. 1050 m. 40º44'53"N 21º11'05"E. 30.07.1998, R. Eisenblätter & E. Willing (B10 0591799)
GREECE: Ioannina, Ep. Metsovou, SW Kranea. Kiefernwald, Bachbett, Serpentinwand, 39º52'33"N 21º15'00"E, 1000 m, 18.07.2000, R. Eisenblätter & E. Willing (B10 0591864).
GREECE: Grevena, Ep. Grevenon, SO Smixi. Sumpfweise in farnreichen Bergwiesen. 1050m. 40º02'53"N 21º09'29"E. 22.07.2000. R. Eisenblätter & E. Willing (B 10 0591887)
GREECE: Ioannina, Ep. Metsovou, Milea. Buchenwaldrand an Bachlauf im Ortseingang. 1090m. 39º51'13"N 21º13'33"E. 18.07.2000, R. Eisenblätter & E. Willing (B10 0591889).
ITALY: Venetia. Patavium (Padova) sponte in R. Horto Botanico, solo macriori et depauperato, 06.1920, A. Béguinot (B 10 0057645)
SERBIA: In viis silvarum pr. Ripanj, Belgrad, 1887.V, Bornmüller (B10 0591026)
SIRIA: Libani in reg. subalp. Jugi Sanin, 17.06.1897, J. Bornmüller (B10 0591019)
SIRIA: Libani in reg. subalp. Jugi Sanin, --.07.1897, 1600 m, J. Bornmüller (B10 0591020)

3b. *Sonchus glaucescens* subsp. *graecus* (Weiss) Mejías & S.-Ch. Kim

CROATIA: Insel Cherso, Hallstatt, Lanterna, Õlhamie, 8.5.1932, Friederich Morton (B10 0095221)
CROATIA: Insel Cherso, Hallstatt, Lanterna, 3.5.1932, Friederich Morton (B10 0095222)
CROATIA: Wüste Stellen bei S. Andrea nnveis Triest, 04.1886, Verouvé (B 10 0591022)
CROATIA: Wüste Stellen um venen Hafnio, 05.05.1886, Verouvé (B10 0591023)
CROATIA: Seestrand bei Veruda, 02.05.1902, K. Untchj (B10 0591024)
CROATIA: Zadar, Mauern, 15.04.1886, J. Bornmüller (B10 0591027)

CROATIA: Ragusa. 21.05.1886, J. Bornmüller (B 10 0591029)

CROATIA: Ragusa-Lapad. 28.05.1886, J. Bornmüller (B 10 0591030)
GREECE: Achaia, Ep. Patron, SW Petas. Ölbaumhain, krautige Böschung. 170m. 09.04.2001. 38º02'55"N 21º28'35"E, R. & E. Willing (B10 0059874).
GREECE: Arkdhia, Ep. Gortynias, SW Livadhaki. Felswand, Kiefern, Q. coccifera-Gebüsch. 430m. 37º37'42"N 21º47'41"E. 18.04.2001. R. & E. Willing (B 10 0059877)
GREECE: Serres, O Serres. Kiefernwald, Straßenrand. 100 m. 04.05.2006. 41º05'51"N 23º33'41"E. R. & E. Willing (B10 0235611).
GREECE: Grevena, S Mikrolivado, 15.05.2012, Kiefernwald, Felsanriss, 970 m, 39º57'16"N 21º13'29"E, R. & E: Willing (B 10 0500477)
GREECE: Lesvos, Mitilini Airport, c. 5 km SE of Mitilini Town, chalky pebbles in roadside by the beach, 0-2m, 39º03'N 26º36'E, H. Nielsen (B 10 0591032)
GREECE: Larissa, Efrimeno, river Pinios-Delta N of Stomio, dune vegetation. On coastal deposits, 1-3 m, 06.05.1999, 39º52'24"N 22º44'01"E, A. Schuler (B 10 0162233)
GREECE: Imathia, Naussa, Vermio, above Kato Seli near Katafigo, burned plots in *Pinus nigra* forest, on limestone, 1250-1270m, 40º32'N 21º56'E, 23.06.2001, A. Schuler (B10 0162234)
GREECE: Thesprotia, SW Paleochori, 08.05.2005, Hartlaubgebüsch, Krautfluren, Stranßenrand, 100 m, 39º32'08"N 20º26'02"E, R. & E. Willing (B 10 0211747)
GREECE: Arta, W Kapsala, 22.05.2005, Felshang, Tannen-Laubwald, 700 m, 39º22'53"N 21º14'45"E, R. & E. Willing (B10 0211763)
GREECE: Arta, SO Koryfi, 22.05.2005, Felsige, Krautfluren, 700 m, 39º24'23"N 21º16'07"E, R. & E. Willing (B10 0211764)
GREECE: Serres, W Livadhia, 29.04.2006, Pappeln, Krautfluren, 85 m, 41º15'27"N 23º04'27"E, R. & E. Willing (B10 0235606)
GREECE: Serres, SSW Promachonas, 30.04.2006, Laubgebüsch, Krautfluren, 80 m, 41º21'26"N 23º21'12"E, R. & E. Willing (B10 0235608)
GREECE: Serres, NO Sidhirokastro, Q. coccifera-Geb., Kiefern, Krautfluren. 140 m. 02.05.2006. 41º15'33"N 23º24'11"E. R. & E. Willing (B10 0235609)
GREECE: Evrytania, SW Dafni. Felsanriss, Krautfluren, Q. coccifera-Geb. 570 m. 39º01'41"N 21º43'29"E. 12.06.2007. R. & E. Willing (B 10 0281676)
GREECE: Fthiotis, Paleovracha, Ölbaumhain, Straßenrand, Krautfluren. 245m. 38º54'36"N 22º03'42"E. 09.06.2007. R. & E. Willing (B 10 0281678)
GREECE: Fthiotis, SO Paleokastro, 01.06.2007. Felswand, Q. coccifera-Geb., Krautfluren, 480 m, 38º58'41"N 21º54'42"E, R. & E. Willing (B 10 0281683)
GREECE: Karditsa, SW Kedros. Hartlaubgebüsch. 175m. 39º11'13"N 22º02'13"E. 30.05.2007. R. & E. Willing (B 10 0281686)
GREECE: Trikala, W Taxiarche. Kiesgrube in Felsfluren. 80m. 39º34'41"N 21º53'46"E. 24.05.2007. R. & E. Willing (B 10 0281693)
GREECE: Trikala, S Parapotamo. Ruderalfluren, Krautfluren. 110 m. 39º31'50"N 21º42'21"E. 23.05.2007. R. & E. Willing (B 10 0281694)
GREECE: Larisa, Chalkiades, 21.04.2008, Straßenrand, Krautfluren am Ortsrand, 260 m, 39º23'42"N, 22º 25'33"E, R. & E. Willing (B 10 0315476)
GREECE: Larisa, SW Mandra, 19.04.2008, Acker, ausgedehnte Krautfluren, Steinbruch, 105 m, 39º36'51"N 22º14'53"E, R. & E. Willing (B 10 0315477)
GREECE: Arkadhia, SO Stemnitsa, 20.06.2010, Felsanriss, Tannenwald, 1050m, 37º32'21"N 22º07'22"E, R. & E. Willing (B 10 0371333)
GREECE: Arkadhia, NO Elati, 19.06.2010, Mischwald, Krautfluren, 1055 m, 37º37'40"N 22º09'18"E, R. & E. Willing (B 10 0371335)
GREECE: Trikala, NO Megali Kerasea, 20.06.2012, Serpentin-Hänge, Straßenrand, 630 m, 39º45'17"N 21º29'35"E, R. & E. Willing (B10 0500440)
GREECE: Trikala, Kalambaka, 18.06.2012, Laubwald, Felswand, 430 m, 39º43'16"N 21º37'53"E, R. & E. Willing (B 10 0500442)
GREECE: Kozanis, S Xirolimni, 29.05.2012, Straßenrand Krautfluren, 690 m, 40º17'25"N 21º39'32"E, R. & E. Willing (B 10 0500462)

GREECE: Kozanis, S Siatista, 27.05.2012, Wiesige Felsfluren, 800 m, 40º14'27"N, 21º33'42"E, R. & E. Willing (B 10 0500464)
GREECE: Grevena, SW Grevena, 17.05.2012, Ruderalfluren, Ackerrand, Krautfluren, 560 m, 40º04'25"N 21º24'34"E, R. & E. Willing (B 10 0500475)
GREECE: Ioannina, W Mikra Peristeri, 13.05.2012, Laubgebüsch, Krautfluren, 640 m, 39º45'14"N 21º04'53"E, R. & E: Willing (B10 0500478)
GREECE: Ioannina, W Mikra Peristeri, 13.05.2012, Laubgebüsch, Krautfluren, 640 m, 39º45'14"N 21º04'53"E, R. & E: Willing (B10 0500478)
GREECE: Lesvos, Mitilini Airport, c. 5 km SE of Mitilini Town, chalky pebbles in roadside by the beach, 0-2m, 39º03'N 26º36'E, H. Nielsen (B 10 0591032)
GREECE: Santorin-Archipiels, Nea Kaimeni: Nordhafen und Krater von 1570. Steile Felswände, Schuttfächer und geschlossene Lupinus-Hyparrhenia-Rasen, Lava Rohboden, 5-50 m, 05.05.1984. Th. Raus (B10 0591033)
GREECE: Kriti: Amari, Bergzunge NW von Klisidhi, 35º16'30"N 24º37'30"E, 525m, 18.06.1983, H. Risse (B 10 0591036)
GREECE: Frindort: in Gafarfalitarum bai Ravadifion, 05.1966, Lipped (B 10 0591041) handwritten, illegible
GREECE: Peloponnesus: Achaia, prope pagum "Kalavryta", versus coenoc. Hagros Lavra, 800 m, 03.05.1926, J. Bornmüller (B 10 0591044)
GREECE: Attica: in arvis. Legimus in agro Acharnano ad radices m. Parnethis, 08.05.1895, (B 10 0591045, Heldreich Herbarium Graecum Normale)
GREECE: Kastrades (Corfú); an einem Graben im Park der Casa rossa, 3 m, 9/6.1896, C. Baenitz (B 10 0591046)
GREECE: Insula Thasos, in alvieto ad Limenas, 21.05.1891, E. v. Halácsy (B10 0591047, Herbar J. Bornmüller)
GREECE: Insula Thasos: In pinetis montis Ipsarion inter Prinos et Hag. Panteleimon, c. 800-1000 m, 12.07.1973, K. H. Rechinger (B10 0591048)
GREECE: Naxos, Potamia, 50 m, 37º05'18"N 25º24'48"E,feutche, S-exp., Straßenböschung, Granit, 21.03.1992, N. Böhling (B 10 0591051)
GREECE: Ep. Kidhonia: Kirche Ajios Elefsa an der Straße zwischen Nerokouros und Malaxa (35º28'N/24º03'30"E); feuchter Straßengraben, ca. 400 m, 11.05.1983, H. Risse (B 10 0591052)
GREECE: Attica: in arvis. Legimus in agro Acharnano ad radices m. Parnethis, 08.05.1895 (B 10 0591054, De Heldreich Herbarium Graecum Normale)
GREECE: Attica: in arvis. Legimus in agro Acharnano ad radices m. Parnethis, 08.05.1895 (B 10 0591054, De Heldreich Herbarium Graecum Normale)
GREECE: Drama, Paranestion, flood-plain habitats of river Nestos, c. 1 km S of Paranestion, ruderal habitats, river banks, on alluvial deposits, 100m, 30.05.2001, 41º16'N 24º31'E, A. Schuler (B10 0478162)
GREECE: Magnisia, Ep. Volou, 1.0km NO Afete, Hartlaubgebüsch, 39º17´N 23º10'E, 320 m, 16.05.1993, Willing (B10 0591064)
GREECE: Ioannina, Ep. Metsovou, SO Flambourari, S Tsouka Arosia. Hangböschung in Kiefernwald, 1150m, 39º50'03"N 21º02'34"E, 17.07.2000, R. Eisenblätter & E. Willing (B10 0591795)
GREECE: Kardhitsa, 7.9km NW Kardhitsa. Ackerrand, feuchter Graben, 40m, 39º25'31"N 21º52'39"E, 01.05.2000, R. Eisenblätter & E. Willing (B10 0591798)
GREECE: Nom. Magnisia, Ep. Almirou, 3.4 km SO Almiros, Ackerrand 13.05.1993, 70-100m, 39º09'N 22º46'0-30"E, Willing (B 10 0591822)
GREECE: Nom. Magnisia, Ep. Almirou, 4.0 km NO Vrinena, 07.05.1993, Felsige Hangböschung, Q. coccifera-Geb. und Straßenrand, 39º05'39"N 22º50'E, 150 m, Willing (B 10 0591833).
GREECE: Serres, Ep. Serron, Menikion, Moni Aj. Pnevmatos. Buschreiche Wiesen, 41º06'30"N 23º41'E, 450 m, 30.06.1992, Willing (B10 0591838)
GREECE: Nom. Etolia-Akarnania, Ep. Nafpaktias, 2.2km NW Pokista, Krautreiche Spartium-Böschung, 38º34'N 21º48'E, 560m, 20.05.1991, Willing (B10 0591842)
GREECE: Nom. Fokis, Ep. Dhoridhos, Trikorfa-Massiv, 0.4 SO Tihio, Lichter Eichenwald auf erodierten Sa-Hängen m. wechselfuchten Wiesen, 38º29'N 22º00'30"E, 640 m, 14.05.1991, Willing (B 10 0591844)
GREECE: Nom. Evrytania, 3.1 km WNW Karpenisi, Felsanriß unter Tannenwald, 39º55'30"N 21º45'30"E, 1120m, 01.06.1991. Willing (B 10 0591845)

GREECE: Lakonia, Ep. Epidhavrou Limiras, NO Vrontamas, Felder mit Ölbäumen, 36º58’29”N 22º39’38”E, 170 m, R. Eisenblätter & E: Willing (B 10 0591847)
GREECE: Lakonia, Ep. Epidhavrou Limiras, N Peristeri, Ölbaumhain, sandig, Felder, 36º52’45”N 22º40’34”E, 40 m, R. Eisenblätter & E. Willing (B 10 0591848)
GREECE: Arkadhia, Ep. Kynourias, SW Poulithra. Ölbaumhain, Straßenrand, 37º06’05”N 22º53’04”E, 230 m, 06.04.1998, R. Eisenblätter & E. Willing (B10 0591857)
GREECE: Ioannina, Ep. Konitsis, SO Armata. Fast bewuchsloe Serpentinhänge, einz. Kiefern und Wacholder, 40º01’38”N 20º59’05”E, 1100 m, 25.07.1998, R. Eisenblätter & E. Willing (B10 0591858)
GREECE: Trikala, Ep. Trikalon, 7.0km NW Trikala, Ruderalfluren, 39º36’38”N 21º43’16”E, 80 m, 28.04.2000, R. Eisenblätter & E. Willing (B10 0591868)
GREECE: Ioannina, Ep. Konitsis, ONO Konitsa. Kiefernwald, 40º03’25”N 20º46’15”E, 820 m, 20.07.1998, R. Eisenblätter & E. Willing (B10 0591870)
GREECE: Ioannina, Ep. Dhodhonis, Tsepelovo. Ruderalfluren an Quelle. 1000 m. 39º54’11”N 20º49’09”E. 17.07.1998, R. Eisenblätter & E. Willing (B10 0591873)
GREECE: Magnisia, Ep. Volou, 1.0 km O Anhialos, Verkrautete Ölbaumhaine und aufgelassenes Weinfeld, 20 m, 39º16´30”N 22º54’E, 05.05.1993, Willing (B10 0591878)
GREECE: Ioannina, Ep. Metsovou, S Katara-Paß. Serpentinhänge m. Kierfern und Wacholder. 1520m. 39º47’34”N 21º12’37”E. 19.07.2000, R. Eisenblätter & E. Willing (B10 0591883)
GREECE: Grevena, Ep. Grevenon, SW Grevena. Ruderalfluren, Straßenrand. 40º04’06”N 21º24’54”E. 470m. 21.07.2000. R. Eisenblätter & E. Willing (B 10 0591886)
Arkadhia, Ep. Mandinias, NO Kandhila. Felswand, Q. coccifera-Gebüsch, 950 m. 02.05.2001. 37º46’57”N 22º23’27”E. R. & E. Willing (B10 0059869)
GREECE: Fthiotis, Ep. Lokridhos, 2.8km SO Aj. Konstantinos. Aufgelassener Acker, 38º44´N 22º52’E, 220 m, 01.05.1993, Willing (B10 0591900)
GREECE: Nichoria Ridge, 0.6 km NE of Karpofora, edge of excavation trench, 25.05.1973, J.M. Shay (B 10 9011520)
GREECE: Karpofora, roadside, 25.05.1973, J.M. Shay (B 10 9011617)
GREECE: Nichoria Ridge, 0.6 km NE of Karpofora, roadside, 08.05.1973, J.M. Shay (B 10 9011618)
GREECE: Neromilo, roadside, 02.07.1971, J.M. Shay (B 10 9011619)
GREECE: Tsana, 0.7 km NE of Tsana; 2.5 km N of Petalidhi, Dig House, 08.05.1973, J.M. Shay (B 10 9011620)
GREECE: Arkadhia, Ep. Gortynias, NO Neochori. Krautige Farnfluren mit Zypressen und Obst-Baümen. 430 m. 19.04.2001. 37º40’35”N 21º49’10”E. R. & E. Willing (B10 0059868)
GREECE: Evrytania, SW Dafni. Felsanriss, Krautfluren, Q. coccifera-Geb. 570 m. 39º01’41”N 21º43’29”E. 12.06.2007. R. & E. Willing (B 10 0281676)
HERCEGOVINA: ad radices mtis Velež prope Mostar in valle fluvii Neretva- in declibivus lapidosis, solo calcareo, 70 m, 14.05.1970, E. Mayer (B 10 0591021)
ITALIY: Amalfi: Brinshige Ha’nge leer. Maiore, 12-16.04.1939, Bornmüller (B 100057666)
ITALIY: Liguria: inter Rapallo et Zriagli, 29.04.1909, J. Bornmüller (B 10 0057667)
ITALIY: Liguria: auf Krislisisland bis Noli, 8.05.1934, Baschant (B 10 0057668)
ITALIY: Liguria: Portofino, auf Sikarland, 04.1930, Baschant (B 10 0057669)
MONTENEGRO: Cattaro, 15.05.1886, J. Bornmüller (B10 0591031)

BC: INSTITUT BOTÀNIC DE BARCELONA. BARCELONA, SPAIN

1. *Sonchus asper* (L.) Hill

AUSTRIA: Steyregg.13/09/1887, D. Rauscher (BC-35693)

FRANCE: Dijon (Côte-d'or).--/09/1922.Mme. de Chamberet (BC-858788)
FRANCE: Gedre.08/04/1907.F.re Sennen (BC-858787)
FRANCE: Villeneuve (Lot et Garonne).18/07/1926.F.re Sennen (BC-858793)
ITALY: Venecia, Padua, --/06/1920. A. Beguinot (BC-35681)
SPAIN: Baleares, Isla de Cabrera. 20/04/1948, P.F. (BC-104032)
SPAIN: Barcelona, Calders. no date, Sra. Gallardo (BC-130204)
SPAIN: Barcelona, Collsacabra. --/07/1886, Vayreda (BC-655554)
SPAIN: Barcelona, Dosrius. 20/07/1944, P. Montserrat (BC-618116)
SPAIN: Barcelona, Tordera. --/05/1893, Vayreda (BC-654823)
SPAIN: Burgos, Bugedo. --/--/1909, H.no H. Elías (BC-858784)
SPAIN: Cádiz, Puerto de Santa María.25/05/1951. A. et O. de Bolós (BC-115823)
SPAIN: Gerona, Agullana.21/06/1908.F.re Sennen (BC-858778)
SPAIN: Gerona, Cerdanya.20/08/1916. F.re Sennen (BC-35698)
SPAIN: Gerona, Ripollés (Vidrá).25/06/1952. A. et O. de Bolós(BC-118241)
SPAIN: Gerona, Sant Llorenç de la Muga.15/05/1905.F.re Sennen (BC-858780)
SPAIN: Gerona, Vidreres.20/05/1945.Font i Quer (BC-631917)
SPAIN: Lérida, La Noguera (Linyola).11/06/1962. F.Masclans (BC-597907)
SPAIN: Lérida, Maldá.11/06/1972.Abel Boldú.(BC-631593)
SPAIN: Tarragona, Cambrils.08/04/1917.F.re Sennen.(BC-35677)
SPAIN: Murcia, Sierra de Espuña.27/06/1930. Hno. Jerónimo (BC-858785)
SPAIN: Teruel, --/08/1909.F.re Sennen (BC-858782)
SPAIN: Teruel, Monreal del Campo. No date. J. Benedicto (BC-35691)
SPAIN: Teruel, Orihuela del Tremedal.15/08/1936. Font i Quer (BC-642949)

3a. *Sonchus glaucescens* Jord. subsp. *glaucescens*

FRANCE: Marsella.01/05/1866.Emile Ballé (BC-133338)

3b. *Sonchus glaucescens* subsp. *graecus* (Weiss) Mejías & S.-Ch. Kim

ITALY: Près Pise.--/--/1868. Henry Van Heurek (BC-651462)
ITALY: Potenza.10/06/1925.O. Gavioli.(BC-76020)

BM: THE NATURAL HISTORY MUSEUM. LONDON, ENGLAND.

1. *Sonchus asper* (L.) Hill

FRANCE: Tour du Valat, S.E. of Mas; by irrigation canal, 11.June.1968, Lynn Kendrick & Elizabeth G. Moyes (nº. 216) (BM000759594)

3a. *Sonchus glaucescens* Jord. subsp. *glaucescens*

FRANCE: Var. Toulon, rochers maritimes au Cap-Brun, sol silicieux, 15 m, 16 avril 1906, L. Verguin (BM000759622)
FRANCE: Var. Toulon, rochers maritimes au Cap-Brun, 16.IV.1906, L. Verguin (BM000759589)

FRANCE: Hyéres, Var, France. No date, no collector, (Herb. G.W.T.H. Fleming) BM000759584

3b. *Sonchus glaucescens* subsp. *graecus* (Weiss) Mejías & S.-Ch. Kim

ITALY: Ravello (Salerno), al basis di Sta. Chiara, in ruderatis, c. 200 m, 14.IV.1912. no collector (BM000759596)
ITALY: Leucaspide (Taranto), in olivetis, 24.III.1920, flos aureus, duplo major quam in 13/20, sin recolector (Herb. Lacaita) (BM000759597)
ITALY: Gravina di Leucaspide (Taranto), 29.4.1881, sin recolector (Herb. C.C. Lacaita) (BM000759598)
ITALY: Pienzza. Maji. --? (BM000759599)

C: UNIVERSITY OF COPENHAGEN, DENMARK

1. *Sonchus asper* (L.) Hill

SPAIN: Isla de Ibiza, Cala Llonga, affaldsdynge. Ostkysten, 13.IV.1968, Hansen, (C 67/2002/16) Graubünden, Föhrenbez, Chur, 620m auf Gartenland, IX.1929, Hatz, Herb. Morten P. Porsild, (C 67/2002/20)
SWITZERLAND: Graubünden, Föhrenbez, Chur, 620m auf Gartenland, IX.1929, Hatz, Herb. Morten P. Porsild, (C 67/2002/20)

3b. *Sonchus glaucescens* subsp. *graecus* (Weiss) Mejías & S.-Ch. Kim

ITALY: Campania.Nápoles, Westhänge des Vesuv in der Nähe der StraBe, Lavaboden, ca. 900 m, 5.VI.1968, Lippert & Zollitsch, (C 67/2002/3)

COI: UNIVERSITY OF COIMBRA, PORTUGAL

1. *Sonchus asper* (L.) Hill

ITALY: Palermo.--/04/--.Todaro.(COI-2/1113)
SPAIN: Isla de Menorca.14/04/1955.N.V. Lletró.(COI-3/1113)

FCO: UNIVERISDAD DE OVIEDO, SPAIN

**1.** *Sonchus asper* (L.) Hill

SPAIN: Luarca, Canero en la playa de Cueva, 18.VII.1974, *T.E. Díaz*, (FCO-11420)

FI: UNIVERSITA DEGLI STUDI DI FIRENZE. FIRENZE, ITALY

**1.** *Sonchus asper* (L.) Hill

ITALY: Isola de Giannutri (Toscana). 20/05/1977. P.V. Arrigoni & P.L. di Tommaso (FI-4459/11)
SWITZERLAND: Châtelaine, près de Genéve (Suisse): lieux cultivés. 13 juin 1884. E. Ayasse. (FI067264)

**3b.** *Sonchus glaucescens* subsp. *graecus* (Weiss) Mejías & S.-Ch. Kim

GREECE: Attica: in arvis. Legimus in agro Acharnano ad radices m. Parnethis. 8 Mai. 1985. J. Dörfler, Wein (FI067267)
ITALY: Sungo la costiera da Portiglione a Torre delle Civette (Marimma). 15 Maggio 1892. U. Martelli (FI067265)
ITALY: Cosenza: sopra Spezzano grande. 22-V-1898. A. Treda. (FI067261)
ITALY: Monterotondo (Roma) 01/06/1951. C. Steimberg (FI-4459/7)
ITALY: Region Toscana. --/03/1863. O. Beccari (FI-4459/9)
ITALY: Region Toscana. 19/05/1892. U. Martelli (FI-4459/10)

G: CONSERVATOIRE ET JARDIN BOTANIQUES DE LA VILLE DE GENÈVE. GENEVE, SWITZERLAND

**1.** *Sonchus asper* (L.) Hill

ITALY: Veneto. Venecia. Patavium *(Padova)*, spronte in R. Horto Botanico. --/VI/1920. Adr. Fiori et A. Beguinot, herb. Delessert, (G-8886/41)
MOROCCO: Yebala. In quercetis suberis, ad pedem Yebel Sugna, 26.V.1928, Font i Quer, herb. Delessert, (G-8886/73)
SPAIN: Burgos. Santo Domingo de Silos. 17/07/1979. J. Fernández Casas, A. Pons-Sorolla, Susanna (G-8886/67)
SWITZERLAND: Vaud. Athenaz (Ginebra), 15.VI.1982, Haueter, herb. Genavense (G-8886/124)
SWITZERLAND: Zurich. Gschwendli, 13.VIII.1968, *Geissler*, herb. Patricia Geissler (G-8886/83)
SWITZERLAND: Vaud. Ginebra, 19.VII.1883, Schmidely, herb. Auguste Schmidely.(G-8886/109)

2. *Sonchus nymanii* Tineo & Guss.

ITALY: Sicilia. Mirto, V.1880, *Todaro*, herb. de Candolle, (G-8886/31)

3a. *Sonchus glaucescens* Jord. subsp. *glaucescens*

FRANCE: Bandol (Alpes Maritimes). 27/05/1899. G.R.Chodat (G-8886/24).
SPAIN: Marseille. --/05/1848. M. Cadet de Fontanai? (G-8886/59).

3b. *Sonchus glaucescens* subsp. *graecus* (Weiss) Mejías & S.-Ch. Kim

FRANCE: Var (Garrigues au Vieux-Cannet).25/04/1952.J. Bouchard (G-8886/65)
FRANCE: St. Martin du Var (Var).22/05/1861.E. Bourgean.(G-8886/51)
FRANCE: Nizza.02/05/1852.Friedrich Sigmund Alioth.(G-8886/53)
FRANCE: La Farlède (Var).17/05/1905. A. Albert. (G-8886/56)
SWITZERLAND: Mies (Vaud). 20/04/1937. O. Meylan(G-8886/54)

GDAC: UNIVERSIDAD DE GRANADA. GRANADA, SPAIN

1. *Sonchus asper* (L.) Hill

SPAIN: Córdoba. Sierra Morena, prox. Alto de Españares. 4.VI.1993. Melendo (GDAC 41929)
SPAIN: Granada. El Purche, 5.VIII.1983, Cueto, (GDAC 15042)

JACA: INSTITUTO PIRENAICO DE ECOLOGÍA, C.S.I.C. JACA, SPAIN

1. *Sonchus asper* (L.) Hill

SPAIN: Ceuta.15/06/1959. F. González Bernáldez (JACA-269232)
SPAIN: Guadalajara. Algora. 12/06/1974. P. Montserrat (JACA-249174)
SPAIN: Huesca, Bielsa. 10/07/1978. P. Montserrat (JACA-187178)
SPAIN: Huesca, Bierge. 22/05/1970. P. Montserrat (JACA-118070)
SPAIN: Huesca, Biescas. 05/09/1969. P. Montserrat (JACA-631069)
SPAIN: Huesca, Bonansa. 01/08/1987. G. Montserrat (JACA-747587)
SPAIN: Huesca, Sariñena. 11/05/1980. G. Montserrat (JACA-30580)
SPAIN: Huesca, Sasal-Sabiñáningo, solana incendiada con pastos secos, P. Montserrat (JACA13194)
SPAIN: Huesca, Siresa. 24/06/1969. P. Montserrat (JACA-338769)
SPAIN: Navarra, Monreal.17/06/1970. L. Villar (JACA-237670)

SPAIN: Zaragoza, Luesia. 14/05/1988. O. Escudero Nogue (JACA715388)
SPAIN: Zaragoza, Berrueco. 06/071972. P. Montserrat & L. Villar (JACA-448672)

L: NATURALIS BIODIVERSITY CENTER. NETHERLANDS, LEIDEN

1. *Sonchus asper* (L.) Hill

CROATIA: Dalmatië. Split, 29.IV.1940, Radermacher, National herb. Nederland (L 0488554)
FRANCE: Pas de Calais.Ambleteuse, Blanc Nez, haverakker bij het standbeeld van Latham, 75 m, 30.VII.1955, Touw, Rijksherbarium Leiden (L 0212520)
SWITZERLAND: Vaud. Leysin (wot), 5.IX.1921, Snuit, National herb. Nederland (L 0488560)

3b. *Sonchus glaucescens* subsp. *graecus* (Weiss) Mejías & S.-Ch. Kim

CROATIA: Dalmatië. Suidyde Marjan. Split. 200 m. 2.IV.1940, *Radermacher*, National herb. Nederland (L 0488555)
FRANCE: Var. Hyères, Forêt des Maures ten Oosten van Hyères, 15.V.1935, Huysman, National herb. Nederland (L 0488547)
ITALY: Liguria. Sestri Levante, 23.V.1962, Kern & Reichgelt, National herb. Nederland (L 0488552)

MA: REAL JARDÍN BOTÁNICO. MADRID, SPAIN

1. *Sonchus asper* (L.) Hill

PORTUGAL: Estremadura, Gourelha, Morelinho, pr. Sintra, 26.III.1992, Aedo & al. (MA507078)
SPAIN: Jaén, Orcera, Barranco del río Madera, 6.VI.1980, Pajarón (MA508244)
SPAIN: Ávila, Ojos Albos, encinar nitrificado, 3.VIII.1984, Burgaz & al. (MA506690)
SPAIN: Zamora, Ribadelago, 1000 m, 14.VIII.1987, García & Rey (MA510723)
SPAIN: Guadalajara, Chillarón del Rey, 21.V.1970, Bellot & al. (MA194093)
SPAIN: Granada, Baza, Cortijo de la Canaleja Alta, 18.VI.1992, Aedo & al. (MA508844)
SPAIN: Moncayo, zonas húmedas, octubre 1982, Burgaz, Fuentes & Mendiola (MA283991)

MPU: UNIVERSITÉ DE MONTPELLIER. MONTPELLIER, FRANCE

1. *Sonchus asper* (L.) Hill

FRANCE: Montpellier, la Vércine, 1.mai.1841 DC. Prd.(MPU)
FRANCE: Aude: ile de l'Aute, juin 1951, SfA (MPU, Herbier Albaille).
FRANCE: Prés de Montpellier, 15-5.1887, Jane de Morin (MPU).
FRANCE: Dans le canton de St. Chapter (Gard), mai 1822, Lombarell Dumas (MPU).
FRANCE: Chemin de Bayard (Hautes Alpes), 27 juin 1895, A. Faure (MPU, Herbier A. Faure).
FRANCE: Pr. St. Germain Mareil (Seine et Oise), 15 Sept. 1855, W. de Schoenefeld (MPU, Herbier du Dr. Sourd).
ISRAEL: Zichiron Jacob, Asie Mineur (Palestine), Fevrier 1906, Aaronsohn (MPU).
ITALY: Palermo. In cultis humidis, aprili sin año, Todaro (MPU, Todaro Flora Sicula exiccata).
SWITZERLAND: Châtelaine, près de Genève (Suisse): lieux cultivés, 13 juin, 1884, E. Ayasse.

3a. *Sonchus glaucescens* Jord. subsp. *glaucescens*

FRANCE: Rochers calcaires dabord de meslà Ste. Marguerita près de Toulon (rare), 8 mai 1870, illegible (MPU, Herbier André)
FRANCE: Toulon, escarpements, Cap Brun, 3 avril 1874, André (MPU, Herbier André)
FRANCE: Var, Toulon, au Cap Brun. Rochers maritimes silicieux, 17 avril 1904, L. Verguin (MPU)
FRANCE: Env. de Toulon Fort Ste. Marguerite, mai, 1848, E. Bourgeau (MPU, Herbier du Dr. Le Sourd)
FRANCE: Rochiers maritimes pour L'Estaque (Marseille. Ecahnatillons rabougris mais la plante n'est pas commune. Juin, 1870, no collector (MPU, Herbier Diomède Tuczkievicz)
FRANCE: Toulon. Sous le fair Lamalgue, 2 avril 1859, Gr. De Lasamlig (MPU, Herbier Bernardin Matin)
FRANCE: Toulon, bords de la mer pres du Cap Brun, 10 mai 1881, Espaque (MPU; Herbier Bernardin Martin).

3b. *Sonchus glaucescens* subsp. *graecus* (Weiss) Mejías & S.-Ch. Kim

GREECE: Insula Paros. Prope Naussa, 6.V.1898, Chr. Leonis (MPU, curavit I: Dörfler nº 104 -1, -2)

SEV: UNIVERSIDAD DE SEVILLA. SEVILLA, ESPAÑA

1. *Sonchus asper* (L.) Hill

MOROCCO: Chefchaouen. Tazhoute: Valle del Oued Talembote. Suelos de descomposición de calizas. 320 m. 14/03/1995. M.a Mateos, M.C. Reina, G. Sangalli, N. Sardón & B. Valdés (SEV139039)
SPAIN: Albacete. Sierra de Alcaraz, entre Bienservida y Villaverde de Guadalimar. 18/07/1985 J. Arroyo & J.A. Mejías (SEV126489)
SPAIN: Asturias. Subida a Monasterio de Hermo. 14/08/1987. M. Barrera & J.A. Mejías (SEV126475)
SPAIN: Ávila. Venta del Obispo. 17/09/1985. P. García & J.A. Mejías (SEV126451)
SPAIN: Cádiz. Chiclana, Marismas de Sancti Petri. 24/04/1985. J.A. Mejías (SEV126472)
SPAIN: Huelva. Hinojos. Pinares. 26/05/1987. J.A. Mejías, P.L. Ortiz & J.M. Polo (SEV126473)

SPAIN: Huesca: Lanavé. Rio Guarga. 13/08/1985. J.A. Mejías, J.M. Polo & C. Romero-Zarco (SEV126445)
SPAIN: Granada. Sierra de Baza. 11/06/1987. J.A. Mejías (SEV126454)
SPAIN: Málaga. El Burgo – Alora. 28/05/1985. P. García & J.A. Mejías (SEV126466)
SPAIN: Murcia. Calasparra. Regadíos. 05/06/1985. P. García & J.A. Mejías (SEV126467)
SPAIN: La Rioja. Entre Baños del Río y Mahave, Río Najerilla. 19/09/1985. P. García-Murillo & J.A. Mejías (SEV126448)

**3b.** *Sonchus glaucescens* subsp. *graecus* (Weiss) Mejías & S.-Ch. Kim

BULGARY: Southern coastal area of Black Sea, plage Silistar v. Resovo, 42º59’12”ºN 27º59’29”E, 23/05/1999, Ch. Gussev & R. Rutherford (SEV281002)
CROACIA: Trstno, Jardín Botánico, 03/10.2018,70 m asl, 42º42’46”N 017º58’35”E, Z. Díaz-Lifante (SEV288163).

TLJ: UNIVERSITÉ PAUL SABATIER. TOULOUSE, FRANCE

**3b.** *Sonchus glaucescens* subsp. *graecus* (Weiss) Mejías & S.-Ch. Kim

FRANCE: Entre Laboline et La Bollinette (Alpes Maritimes), Cultures, Chemins, env. 900m, 16.6.1913, Herbier J. Serres, TL0994919-20

PERSONAL COLLECTIONS

**1.** *Sonchus asper* (L.) Hill

FRANCE: Var, Hyères-Le Lavandou, bosque aclarado con Quercus suber, N 43º08’59.1” E 006º17’25.4”, 27.04.2011, Mejías, Silva & (Mejías - FR5/11; SEV 284731)
GREECE: Kriti: Chania-Rethimon, cercanías del cruce hacia Sfakia, naranjal en suelos margosos, N 35º23.815’ E 024º09.358’, Mejías (Mejías - KR12/11-1, KR12/11-2; SEV)
ITALY: Emilia-Romagna, Cesena-Cervia, campos cultivados, N 44º 12’56.1” E 012º20’00.8”, 29.05.2011, Lim, Mejías & Silva (Mejías - IT5/11B-1; SEV 284728)
ITALY: Sicily, alrededores de Alia, carretera a Montemaggiore, N 37º 46’ 31.6’’ E 13º 40’ 12.6’’, 03.05.2011, Mejías, Lim & Silva (Mejías - IT28/11; SEV 284735)
ITALY: Sicily, Scillato-Caltavuturo, margen de la carretera, 30.05.2011, Lim, Mejías & Silva (Mejías - IT24/11; SEV)
ITALY: Toscana, San Pellegrino in Alpe, 1520m, N 44º11’28” E 010º28’50”, 09.08.2011, Mejías (Mejías - IT30/2011; SEV 284736)
MOROCCO: Central Rif, carretera de Bab-Berreb a Ketama (Issaguen), pequeño claro en un bosque de cedros, 1350 m asl, N 34º59’07.2” W 004º48’29.4”, 14.06.2010, Mejías & Silva (Mejías - MRR5/10; SEV)

MOROCCO: Central Rif, carretera de Bab-Berreb a Ketama, cerca de Ketama, pequeño talud junto a la carretera, cerca de Ketama (Issaguen), 1660 m asl, N 34º57'42.0" W004º39'24.1", 14.06.2010, Mejías & Silva (Mejías – MRR7/10; SEV)
MOROCCO: West Rif, Bab Taza – Bab Berret, prados encharcados, 1402m, N 35º01'05.7" W 005º00'33.5", 14.06.2010, Mejías & Silva (Mejías - MRR3/10; SEV 284733)
MOROCCO: West Rif, Chefchaouen, carretera a Ketama, vegetación de ribera, 570m, N 35º05'51.9" W 005º15'57.6", 14.06.2010, Mejías & Silva (Mejías - MRR2/10; SEV 284732)
MOROCCO: Tangiers, Cabo Espartel, margen de un pinar de *Pinus pinea*, N 35º47'01.3" W 005º53'34.1", 16.05.2011, Mejías & Silva (Mejías - MRR4/11; SEV 284734)
PORTUGAL: Aveiro, Albergaria-a-Velha, roadside, N40º41.399' W008º31.444', 100 m asl, Mejías (Mejías - PNE12/12; SEV)
PORTUGAL: Beja, Vilanova de Sao Benito - Pias, olivares, N37º56.154' W007º26.289', 245 m asl, Mejías (Mejías - PNE2/12; SEV)
PORTUGAL: Coimbra, Adémia de Cima, borde de la carretera y cultivos, N 40º14'59.6" W 008º26'34.5", 29.05.12, Mejías (Mejías - PNE10/12; SEV 284737)
PORTUGAL: Leiria, São Pedro Moel, carretera de salida a Marinha Grande, pinares de *Pinus pinaster*, N39º45.019' W009º00.675', Mejías (Mejías - PNE8/12; SEV)
SPAIN: Albacete, Bienservida, cultivos de almendros, N 38º 30' 34.5'' W 2º 40' 18.9'', 10.05.2012, Mejías & Silva (Mejías - SE10/12; SEV 284742)
SPAIN: Asturias, Carcedo, prados y vía del ferrocarril, N 43º32.476' W 006º01.501', 31.05.2012, Mejías (Mejías - PNE15/12; SEV 284747)
SPAIN: Ávila, Cepeda de la Mora, salida de la N-502 a Cepeda de la Mora, 26.07.2009 Mejías (Mejías; SEV)
SPAIN: Baleares, Ibiza, carretera a Cala Jondals y Cala Molí, espacios aclarados en pinar, N 38º53.250' E 001º19.933', 23.05.2012, Mejías (Mejías - IBZ2/12; SEV 284743)
SPAIN: Baleares, Mallorca, Artá, entrada al Parc de Llevant, N39º44'10.5" E 003º20'05.5", 28.05.2010, Mejías (Mejías - MLL9/10; SEV 284739)
SPAIN: Baleares, Mallorca, Artá, ermita de Betlém, N39º44'14" E003º18'40", 28.05.2010, Mejías (Mejías - MLL13/10; SEV).
SPAIN: Cádiz, Chiclana, Playa de Sancti-Petri, suelo arenoso, N 36º23'01.5" W 006º11'56.2", 29.03.2012, Mejías (Mejías - CA5/12; SEV 284744)
SPAIN: Cádiz, Villamartín – Las Cabezas, campos cultivados, N39º44'10.5" E 003º20'05.5", 29.03.2012, Mejías (Mejías - CA7/12; SEV 284738)
SPAIN: Granada, Riofrío, vegetación de ribera, 500 m, 18/10/2013, Mejías (SEBICOP3/13, SEV)
SPAIN: Huelva, Los Marines-Cortelazor, márgenes de bosque con *Quercus* y *Castanea*, N 37º54.689' W 006º37.118', 28.05.2012, Mejías (Mejías - PNE1/12; SEV 284745)
SPAIN: Huesca, Bonansa, Puerto de Bonansa, orilla de la carretera, 1390m, N 42º25'26.9" W 000º38'48.8", 08.08.2013, Mejías (SEV 284749)
SPAIN: Jaén, Albánchez – Torres, Puerto de Albánchez hacia Torres, 10.05.2012, Mejías & Silva (Mejías - SE5/12; SEV)
SPAIN: Jaén, Bedmar, carretera a Jimena, olivares, N 37º 49' 37.0'' W 3º 26' 01.7'', 10.05.2012, Mejías & Silva (Mejías - SE2/12; SEV 284741)
SPAIN: Jaén, Marmolejo, cruce a Marmolejo en la A-4, N 38º01'36.3" W 004º09'48.0", 09.05.2012, Mejías & Silva (Mejías - SE1/12-1, SE1/12-2; SEV 284740)
SPAIN: Jaén, Torres, road to Jimena, olive groves, 10.05.2012, Mejías & Silva (Mejías - SE8/12; SEV)
SPAIN: Lérida, Caldes de Boí, ribera del río Noguera del Tor, 42,560978ºN 0,841665ºE, 1450 m, 08/09/2012 (SEV)
SPAIN: Soria, Calatañazor, campos cultivados junto a la población, 1025m, N 41º42'00.3" W 002º49'18.6", 12.08.2012, Mejías (SEV 284751).
SPAIN: Soria, Valonsadero Park, chopera, 1040m a.s.l., N 41º48'31.3" W 002º32'39.8", 10.08.2013, Mejías (SEV 284750)
SPAIN: Teruel, Tramacastilla-Noguera de Albarracín, orilla de la carretera, 1330m, N 40º25'56.3" W 001º34'50.7", 02.08.2012, Mejías (SEV 284748)
SPAIN: Zamora, Quiruelas de Vidriales, margen de la carretera, N 42º01.106' W 005º50.952', 30.05.2012, Mejías (Mejías - PNE13/12; SEV 284746)

TURKEY: Pamphylia province, Tassagil-Sagrim, roadside, N 36º59’ E 031º12’22.03.2010, Mejías, Navarro, Talavera & Terrab (Mejías - TK6/10-1; TK6/10-2; TK6/10-3; SEV)

2. *Sonchus nymanii* Tineo & Guss.

ITALY: Sicily, Alia – Roccapalumba, cultivos, N 37º 46’ 31.6’’ E 13º 40’ 12.6’’, 03.05.2011, Lim, Mejías & Silva (Mejías - IT27/11-1, IT27-2; SEV 284705)
ITALY: Sicily, Scillato-Caltavuturo, herbazal en suelo encharcado, N 37º 50’ 34.4’’ E 13º 54’ 14.3’’, 03.05.2011, Lim, Mejías & Silva (Mejías - IT22/11-1, IT22/11-2; SEV 284703)
ITALY: Sicily, Valledolmo, cultivos, carretera a Alia, N 37º 43’ 41.6’’ E 13º 48’ 01.2’’ 03.05.2011, Lim, Mejías & Silva (Mejías - IT26/11-1, IT26/11-2; SEV 284704)

3a. *Sonchus glaucescens* Jord. subsp. *glaucescens*

FRANCE: Var, îles d’Hyères, Port-Cross, construcciones abandonadas y márgenes de caminos, N 43º00’36.1” E 006º22’53.9”, 27.04.2011, Lim, Mejías & Silva (Mejías - FR14/11; SEV 284714)
FRANCE: Var, îles d’Hyères, Port-Cross, margen de un sendero en costa boscosa, N 43º00’18.5” E 006º22’26.4”, 27.04.2011, Lim, Mejías & Silva (Mejías - FR14/11bis; SEV 284715)
FRANCE: Var, Six-Tours-les-Plages, Cap Sicié, île de Gaou, suelos arenosos, N 43º10’03.8” E 005º42’56.1”, 26.04.2011, Lim, Mejías & Silva (Mejías - FR3/11-1, FR3/11-2; SEV 284713)

3b. *Sonchus glaucescens* subsp. *graecus* (Weiss) Mejías & S.-Ch. Kim

FRANCE: Var, Borme-les-Mimosas, La Faviére, N 43º07’06.5” E 006º23’01.4”, Lim, Mejías & Silva (Mejías - FR8/11; SEV)
FRANCE: Var, Cavalière. Plage de la Fossette, N 43º08’40.0” E 006º21’45.1”, Lim, Mejías & Silva (Mejías – FR11/11-1, FR11/11-2, FR11/11-3; SEV)
FRANCE: Var, Hyères-Le Lavandou, bosque aclarado de Quercus suber, N 43º08’58.7” E 006º17’26.5”, 27.04.2011, Mejías, Silva & (Mejías - FR4/11; SEV 284726)
FRANCE: Var, La Croix-Valmer – Saint-Tropez, roadside, N 43º09’55.9‘’ E 006º 30’07.4’’, 27.04.2011, Lim, Mejías & Silva (Mejías - FR12/11-1. FR12/11-2, FR12/11-3; SEV)
FRANCE: Var, cerca de La Gouargue, orilla de la carretera en un alcornocal, N 43º21’23.6” E 006º27’25.4”, 195 m, 27/04/2017; Berjano, Mejías &y Ortiz (Mejías - 111/17; SEV).
FRANCE: Var, Le Muy, carretera hacia Sainte-Maxime (D25), N 43º25’56” E 006º34’09”, 65 m, 27/04/2017; Berjano, Mejías & Ortiz (Mejías - 102/17; SEV).
FRANCE: Var, cercanías a Sainte-Maxime desde Le Muy (D25), N 43º20’30.2” E 006º36’30.3”, 116 m (GPS =20). 27/04/2017; Berjano, Mejías & Ortiz (Mejías - 105/17; SEV)
GREECE: Creta, Aghia Pelagia, bordes de la carretera, 20 m.sm. N 35º23’56.6” E 025º01’05.2”, 08.04.2011, Mejías (Mejías – KR5/11; SEV).
GREECE: Creta, Agios Nikolaus, carrretera a Neapolis, N 35º12.842’ E 025º40.191’, 09.04.2011, Mejías (Mejías - KR20/11SEV 284725)
GREECE: Creta, de Kare a Vrisses, bordes de la carretera, 430 m.sm. N 35º20.328’ E 024º28’12.339”, 08.04.2011, Mejías (Mejías - KR17/11; SEV).
GREECE: Chalkidiki, Paleochoia, carretera a Arnaia, cunetas y cultivos, N 40º30’24.4” E 023º27’42.9”, 310 m asl, 24.05.2014, Mejías (Mejías - TH3/14; SEV)
GREECE: Epiro, entre Paramythia y Mousiotitsa, margen de la carretera junto a un río con bosque de *Platanus orientalis*, 10.05.2010, Mejías, Ortiz, Sánchez & Silva (Mejías - GR67/10)
GREECE: Gorinetissa, carretera Mesolorgi-Arta, margen de la carretera, 28m, N 38º39’03.2” E 021º14’14.5”, 08.05.2010, Mejías, Ortiz, Sánchez & Silva (Mejías - GR60/10; SEV 284723)
GREECE: Grevena, zonas ruderales y cultivos, N 40º05’39,1” E 21º27’02.22”, Mejías, 25.05.2014 (Mejías - TH4/14-1, TH4/14-2; SEV)

GREECE: Meteora, margen de un bosque de Quercus, 539m, N 39º43'31.9" E 021º38'14.7", 11.05.2010, Mejías, Ortiz, Sánchez & Silva (GR74/10; Mejías - SEV 284724)
GREECE: Peloponesus, Eurostini, carretera a Oros Kyllini, cerca de Rozena, 436m, N 38º06'00" E 022º23'51.1", 08.05.2010, Mejías, Ortiz, Sánchez & Silva (Mejías - GR33/10; SEV 284720)
GREECE: Peloponesus, Trapeza-Kalavryta, margen de carretera en un bosque de ribera, 633m, N 38º39'03.2" E 022º12'51.4", 08.05.2010, Mejías, Ortiz, Sánchez & Silva (Mejías - GR41/10; SEV 284721)
GREECE: Peloponnesus, Patras-Kyllini, Playa Achaïa, nivel del mar, N 38º09'09.4" E 021º33'41.6", 08.05.2010, Mejías, Ortiz, Sánchez & Silva (Mejías - GR45/10; SEV 284722)
GREECE: Peloponesus, Puerto Kyllini, groves and *Arundo* reedbeds, close to the beach, N 37º56'22" E 021º08'33", 09.05.2010, Mejías, Ortiz, Sánchez & Silva (GR50bis/10; Mejías - SEV)
GREECE: Thessaloniki, around Thermi, *Pinus halapensis* forest with dense weed vegetation, 40 m asl, N 40º30'45" E 023º,04'40", 24.05.2014, Mejías (Mejías - TH1/14; SEV)
ITALY: Amalfi, costa amalfitana, Maiori, carretera a Salerno, carcanías de la ermita de Santa María de Olearia, N 40º38'13.6" E 14º40'38.7", 01.05.2011, Lim, Mejías & Silva (IT16/11-1, IT16/11-2; Mejías - SEV)
ITALY: Apulia, Lesina, crop fields to Lesina Marina, N 41º53'49.3" E 015º19''13.2", 30.04.2011, Lim, Mejías & Silva (Mejías - IT13/11-1, IT13/11-2; SEV)
ITALY: Arruzzi, San Vito Chietino, áreas ruderales en la cercanía de la playa, N 42º 18' 29.7'' E 14º 26' 48.4'', 30.05.2011, Lim, Mejías& Silva (Mejías - IT7/11; SEV 284729)
ITALY: Campania, Avellino-Torelli, campos cultivados de avellanos, N 40º 54' 09.1" E 14º 40' 16.8", 30.05.2011, Lim, Mejías & Silva (your name) (Mejías - IT14/11; SEV 284730)
ITALY: Chieti, entre Ortona y Torino di Sangro, 30.04.2011, Lim, Mejías & Silva (Mejías - IT11/11-1, IT11/11-2; SEV)
ITALY: Emilia-Romagna, Faenza, área de descanso en la autopista Bologna-Faenza, Cà Bianca, N 44º25'42.1" E 011º35'52.7", 29.04.2011, Lim, Mejías & Silva (Mejías - IT3/11-1, IT3/11-2; SEV)
ITALY: Emilia-Romagna, Cesena-Cervia, campos cultivados, N 44º 12'56.1" E 012º20'00.8", 29.05.2011, Lim, Mejías & Silva (Mejías - IT5/11-1, IT5/11-2; SEV 284728)
ITALY: Liguria, carretera a Passo del Bracco, 129m, N 44º14'20.7" E 009º32'52.0", 28.04.2011, Mejías, Ortiz, Sánchez & Silva (Mejías - IT1/11; SEV 284727)
TURKEY: Antalya province, Belek. Bosque de *Pinus halepensis*, cercano a la playa en ellos alrededores delHotel Maritime Pine Beach, N 36º50'42" E 031º04'25", 25.03/2010, Mejías (Mejías - TK 33/10-1, TK33/10-2; SEV256669)
TURKEY: Antalya. Jardines junto al puerto, N 36.884104º E 31.078359º, 25.03/2010, Mejías (Mejías - TK 34/10; SEV256670)
TURKEY: Balikesir province, carretera Edinick - Erdek, cerca del istmo, carretera hacia la playa, N 40º22'40.0" E 27º53'46.6", 15.05.2010, Ortiz & Silva (Mejías - TK97/10; SEV)
TURKEY: Balikesir province, carretera Edinick-Erdek, cerca del istmo, carretera a la playa, N 40º22'40.0" E 27º53'46.6", 15.05.2010, Ortiz & Silva (Mejías - TK98/10-1, TK98/10-2; SEV)
TURKEY: Izmir province, Bornova-Manisa, N 38º31'56.1" E 027º16'53.5", 13.05.2010, Ortiz & Silva (Mejías - TK88/10; SEV 284717)
TURKEY: Kastamonu province, Bulacik Köyü, campos cultivados de trigo, 1100m, N 41º17'50.2" E 033º46'44.4", 5.05.2011, Mejías & Sánchez (Mejías - TK231/10-1, TK231/10-2; SEV 284719)
TURKEY: Lycia, Finike, carretera a Antalya, pinar, N 36º34'21" E 029º57'19", 23.03.2010, Mejías, Navarro & Talavera (Mejías - TK19/10; SEV 284716)
TURKEY: Pamphylia province, Tassagil-Sagrim, margen de la carretera, N 36º59' E 031º12'22.03.2010, Mejías, Navarro, Talavera & Terrab (Mejías - TK6/10-1, TK6/10-6; SEV)
TURKEY: Samsun province, Terme-Çraşamba, margen de carretera, N 41º13'06.1" E 036º54'26.0", 14.05.2011, Mejías & Sánchez (Mejías - TK223/10; SEV 284718)
TURKEY: Samsun province, Terme-Çraşamba, margen de carretera, N 41º13'06.1" E 036º54'26.0", 14.05.2011, Mejías & Sánchez (Mejías - TK224/10; SEV)
